# A semi-automated quality assurance tool for cardiovascular magnetic resonance imaging: application to outlier detection, artificial intelligence evaluation and trainee feedback

**DOI:** 10.1186/s12911-025-03271-6

**Published:** 2025-12-02

**Authors:** Thomas Hadler, Leonhard Grassow, Johanna Kuhnt, Richard Hickstein, Hadil Saad, Maximilian Fenski, Jan Gröschel, Ralf-Felix Trauzeddel, Edyta Blaszczyk, Clemens Ammann, Darian Viezzer, Anja Hennemuth, Steffen Lange, Jeanette Schulz-Menger

**Affiliations:** 1 Charité – Universitätsmedizin Berlin, corporate member of Freie Universität Berlin and Humboldt-Universität zu Berlin, ECRC Experimental and Clinical Research Center, Berlin, Germany; 2 Working Group on Cardiovascular Magnetic Resonance, Experimental and Clinical Research Center, A Joint Cooperation Between the Charité – Universitätsmedizin Berlin and the Max-Delbrück-Center for Molecular Medicine, Berlin, Germany; 3 DZHK (German Centre for Cardiovascular Research), Partner Site Berlin, Berlin, Germany; 4 Deutsches Herzzentrum der Charité-Medical Heart Center of Charité and German Heart Institute Berlin, Klinik für Kardiologie, Angiologie und Intensivmedizin, Berlin, Germany; 5 Department of Anesthesiology and Operative Intensive Care Medicine, Charité Campus Benjamin Franklin, Hindenburgdamm 30, 12203 Berlin, Germany; 6 Deutsches Herzzentrum der Charité (DHZC), Institute of Computer-Assisted Cardiovascular Medicine, Augustenburger Platz 1, Berlin, Germany; 7 Fraunhofer MEVIS, Bremen, Germany; 8 Department of Computer Sciences, Hochschule Darmstadt - University of Applied Sciences, Darmstadt, Germany; 9 Department of Cardiology and Nephrology, Helios Hospital Berlin-Buch, Berlin, Germany

**Keywords:** Cardiovascular magnetic resonance, Quality assurance, Statistical analysis, Artificial intelligence, Education, Software, Quantitative parameters

## Abstract

**Background:**

Cardiovascular magnetic resonance (CMR) offers state-of-the-art volume, function, fibrosis and oedema imaging. Quality assurance (QA) tasks, such as quantitative parameter reproducibility assessments, the evaluation of AI methods, and the assessment of trainees have become essential to CMR. However, the explainability of how qualitative differences impact quantitative differences remains underexplored. Our aim is to demonstrate a semi-automated QA tool, Lazy Luna’s (LL) applicability to typical CMR QA application cases.

**Methods:**

A software feature error-tracing is designed that allows for quickly pinpointing qualitative reasons for quantitative differences and outliers. Three QA application cases were designed. First, LL was applied to perform **outlier detection** for inter- and intraobserver analyses to detect failure cases and provide qualitative explanations. Outlier detection was performed on several typical images types. Second, LL supported an **Artificial intelligence (AI) evaluation**, in which an AI method was compared to a CMR-expert of 144 patients. LL assessed the acceptability of AI biases for left and right ventricular (LV, RV) end-systolic, –diastolic, and stroke volumes (ESV, EDV, SV), ejection fractions (EF) and the myocardial mass (LVM). Annotations were examined to explain the qualitative differences that resulted in good and poor parameters. The AI investigation was recorded as a video. Third, LL was used to provide a **Trainee Feedback** to a CMR beginner. The trainee was compared to an expert on several imaging techniques to investigate outliers.

**Results:**

For the outlier detection, LL detected segmentation differences that caused parameter differences on multiple sequences. For the AI evaluation calculated clinical parameter biases to be: LVESV:-3.1 ml, LVEDV:2.1 ml, LVSV:6.5 ml, LVEF:3.0 ml, RVESV:0.3 ml, RVEDV:-3.8 ml, RVSV:-4.2 ml, RVEF:-1.4 ml, LVM:-2 g. Inspecting the causes for outlier differences revealed that juxtaposed basal slice failures caused unacceptable LVSV deviations between AI and expert. For the trainee assessment, LL showed that trainee parameters exceeded tolerance ranges. The segmentations could be improved to better mirror expert segmentations and close the parameter gaps.

**Conclusion:**

Lazy Luna, as a semi-automated quality assurance tool, is applicable to several quality assurance application cases in CMR.

**Supplementary information:**

The online version contains supplementary material available at 10.1186/s12911-025-03271-6.

## Background

Scientists and clinicians employ quantitative cardiovascular magnetic resonance (CMR) imaging as the gold standard for cardiac function evaluation and myocardial tissue characterisation [1]. As these quantitative methods mature in CMR, the need for quality assurance (QA) increases in importance. QA aims at optimizing the reliability and accuracy in quantitative CMR by increasing the reproducibility of assessments and standardizing segmentation techniques. Measures, such as the introduction of guidelines, the instruction of trainees as well as the clinical integration of artificial intelligences (AI) are successfully applied to this end [2, 3].

QA in post-processing has been pursued rigorously from several vantage points. Post-processing guidelines have the intention of reducing interobserver and inter-site biases by streamlining the approach to CMR image analysis [1]. Instruction of trainees on the other hand aims at steering CMR trainees towards guideline-consistent segmentations, while gaining familiarity with post-processing software tools and increasing parameter reproducibility [2, 4]. As AIs enter research and clinical routine they achieve clinical parameter assessments close to experts [5, 6]. They promise an increase in CMR efficiency by functioning day and night, scaling indefinitely, and remaining uninfluenced by concentration deterioration [7]. However, AIs continue to make anatomically fragmented segmentations [6, 8], and while they generalise over scanners and sites, reliability across pathologies remains unclear [9, 10]. While AI developers typically focus on quantitative comparisons of annotations, and calculations of clinical parameter differences [5, 6, 11], clinicians may focus on guideline conformance and agreeability with cardiac geometry [1, 12, 13].

**Fig. 1:**
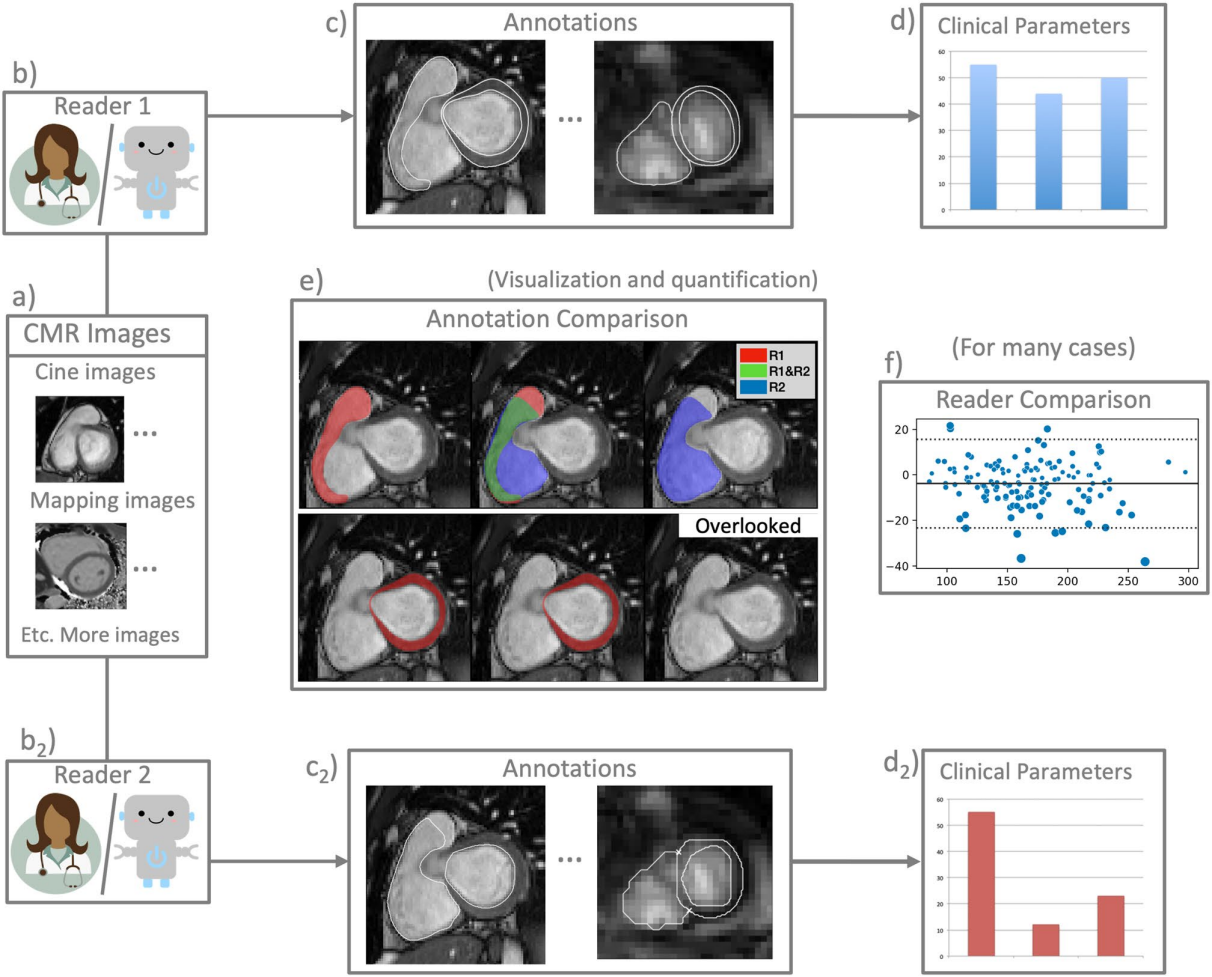
Overview of Lazy Luna (best viewed on monitor). Lazy Luna allows for a multilevel reader comparison. A case consists of CMR images (**a**) of several sequence types, which are annotated by two readers (e.g. either human experts or artificial intelligence methods) (**b,b**_**2**_). Images and annotations (**c,c**_**2**_) allow for the calculation of clinical parameters (**d,d**_**2**_). Lazy Luna offers visualizations and quantifications of annotation comparisons for all segmentations (**e**). Visualizations are color-coded with the first reader in red, the second in blue, and agreement between readers in green (**e**). Quantifications are calculated as segmentation metrics (e.g.: dice similarity coefficient and Hausdorff distance). Statistics can be performed when many cases are annotated by two readers, such as the bland altman plot for clinical parameter differences (**f**)

Although QA has received a respectable amount of attention, CMR remains riddled with significant challenges. On the level of individual images, highly variable annotation decisions for basal and apical slices cause large volumetric differences for AIs and human readers alike [14–17]. Tighter or looser myocardial segmentations may impact the exclusion of partial volume effects and consequently parametric mapping values [18]. Although average clinical parameter differences are close to zero for interobserver assessments the variance between readers remains high – even for expert readers – and problematic for clinical practice [14, 19, 20]. Regardless of the task being the instruction of trainees, the evaluation of AI proficiency, or the assessment of parameter reproducibility: the underlying task is the comparison of two different readers. This task must be augmented with additional information such as acceptable tolerance ranges for deviations of parameters and expert knowledge of acceptable annotation behaviour. In order to evaluate and compare readers on multiple levels of analysis the software tool Lazy Luna (LL) was developed (Fig. 1) [21]. LL allows a user to automatically generate several statistical and per case comparisons (e.g. reader biases between parameter assessments) and investigate causes for differences manually. In another study LLs software architecture was designed to be flexible to adapt to new imaging techniques and comparison methods [22]. LL can compare readers on short- and long axis (SAX, LAX), T1 and T2 parametric mapping, as well as Late Gadolinium Enhancement images, while offering clinically meaningful comparisons, such as American Heart Association (AHA) models of reader differences and segmentation difference visualizations. LL offers all evaluations typical of an AI method assessment while simultaneously giving the user the possibility to investigate qualitative reasons for quantitative differences.

While Lazy Luna’s technical features have been previously introduced, its real-world utility across clinical QA tasks remains untested. No prior work has examined how a single tool can systematically support reader comparison, bias detection, and error tracing across multiple CMR imaging contexts.

The aim of this paper is to assess Lazy Luna as a semi-automated quality assurance tool, and demonstrate its applicability to typical CMR QA application cases: an outlier investigation of clinical parameter assessments on multiple imaging sequences, an AI evaluation with segmentation failure inspection, and producing feedback for a trainee session.

## Methods

The first part of methods “Software Features of Lazy Luna” will focus on software features that facilitate quality assurance tasks. These include the subsections “Tolerance Ranges”, which are statistical tests for reader comparison, “Semi-automatic Reports of Reader Comparisons”, which produce readable documents, “Integrated Error Tracing”, a method for investigating how annotation differences cause reader differences, and a “Summary of Lazy Luna’s capabilities” with information of LLs design from previous papers. The second part of methods “Software Capabilities and Application Tests” focusses on defining LLs hypothesized capabilities and testing them on three QA scenarios.

### Software features of Lazy Luna

#### Tolerance ranges

**Fig. 2:**
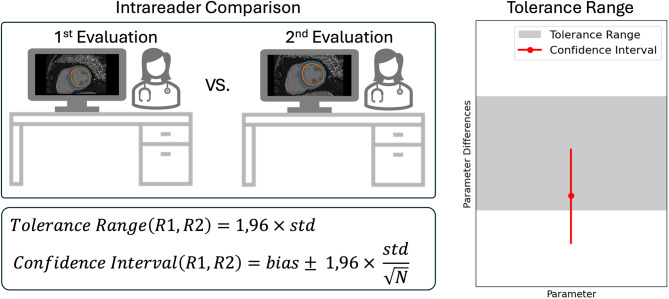
Tolerance ranges and reader bias acceptability. Tolerance ranges are defined for clinical parameters to establish limits for acceptable bias. One expert reader annotates at least 30 cases twice. The distribution of intrareader differences is used to derive a 95% tolerance interval (as shown by the formula). A new reader is evaluated on a separate validation dataset. Their bias is considered acceptable if their 95% confidence interval lies entirely within the predefined tolerance range. The plot on the right illustrates a case where the confidence interval of the new reader’s bias leaves the tolerance range

Tolerance ranges are used to evaluate whether a new reader’s bias falls within an acceptable interval, based on intrareader variability observed in expert annotations (Fig. 2). Tolerance ranges for CMR parameters were introduced in Zange et al. [23] and used in several studies since [11, 17, 24] to define the acceptability of reader biases.

Tolerance ranges complement descriptive statistics (e.g. averages or variance of parameter differences) by providing decision criteria for acceptability of reader differences. Tolerance ranges were integrated into Lazy Luna to assess the acceptability of biases between readers for all volume and function parameters for SAX cine and atrial assessments, as well as global T1 and T2 mapping values. They are used in the QA application cases to decide the acceptability of reader differences. Tolerance ranges are further used to define “Outliers”, which are clinical parameter difference between two readers that exceed the tolerance range.

#### Semi-automatic reports of reader comparisons

LL was extended to provide reader comparison reports in portable document format (PDF) with python bindings of pyfpdf [25]. LL offers two output types; the first is called “Simple Comparison”, the second “Extensive Comparison”. The Simple Comparison provides basic statistical analyses of both readers, as well as explanations that the investigator added while tracing the statistical analyses to their causal origins. The extensive comparison provides more in-depth statistical analyses (e.g. Additional File 1). Reports are produced for all QA application cases and are available in the appendix.

#### Integrated error tracing

**Fig. 3:**
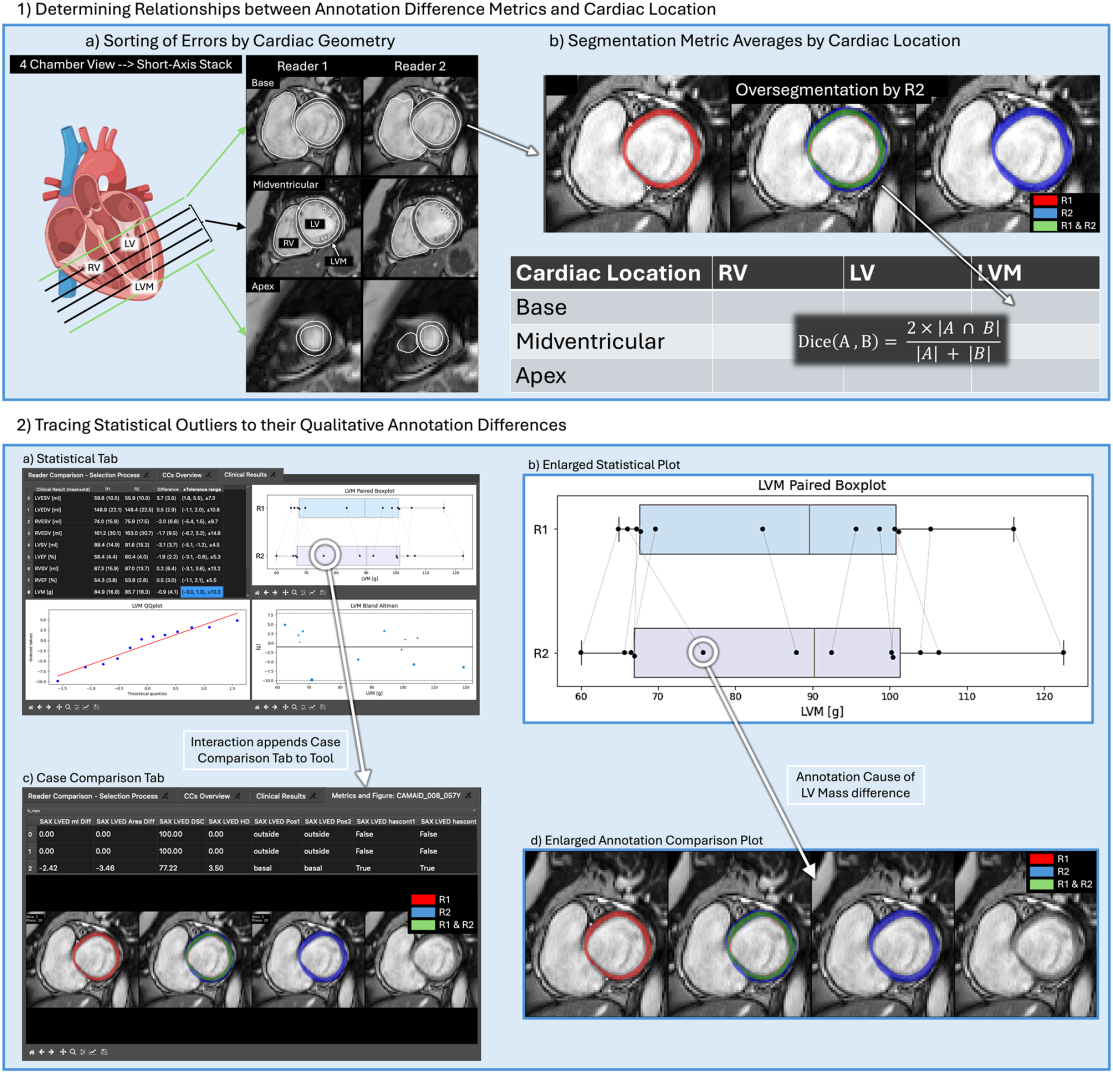
Lazy Luna’s integrated error tracing. Subfigure **1a**) depicts how LL calculates cardiac positions of a stack of SAX cine images. Basal and apical slices can be defined as the first slice segmented by the first reader, the second reader, or according to the four chamber view’s orientation. In **1b**) the oversegmentation by R2 is quantified by annotation metrics (e.g. Dice metric, ml difference), which partakes in the average metric value of the table’s cell for LVM and Base. The information of 1) is output as a spreadsheet. In subfigure **2**) it is demonstrated how the LL UI allows for tracing errors from the clinical parameters to causal annotation differences. In a statistical tab (**2a**), a table for reader averages and three plots are presented to the user (top right: paired boxplot, bottom left: QQ plot, bottom right, Bland-Altman plot). The paired boxplot is enlarged in **2b**). The same cases are evaluated by R1 and R2 and connected by a grey line. Small deviation lead to vertical lines, larger deviations to more horizontal lines. A more horizontal outlier is encircled. After the user clicks on the point a case comparison tab is added to the LL UI (**2c**), in which annotations by both readers are compared. On the top a table of annotation metrics is presented to the user, below a figure for overlapping contours, which is enlarged in **2d**). This allows the user to detect qualitative reasons for clinical parameter deviations. Legend: LL: Lazy Luna, UI: user interface, R1: reader 1, R2, reader 2, RV: right ventricle, LV: left ventricle, LVM: left ventricular myocardium, dice: dice similarity coefficient, HD: Hausdorff distances, SAX: short-axis, LVES: LV end-systole, LVED: LV end-diastole, QQ: quantile-quantile

Error tracing refers to software features that connect several levels of post-processing to each other (e.g. annotation metric values and clinical parameter differences). This encompasses several otherwise unrelated features, such as the calculations of spreadsheets that show the differential volumetric impact of certain cardiac regions (Fig. 3.1, Table 3), or software features that allow the user to interactively investigate qualitative reasons for statistical reader biases or clinical parameter differences (Fig. 3.2). Statistical plots pertaining to reader differences allow the user to examine individual points in the plot (with case name labels) by presenting plots of annotation differences (Fig. 3). This procedure was integrated into LL to store information on error causes as reports (e.g. Additional File 1). Error tracing is employed in the first QA application case to reveal how impactful annotation differences caused large clinical parameter differences. In the second QA application case, error tracing was used to collect representative segmentation failures by an AI method. The third QA used error tracing to illustrate improvement possibilities that with a software feature called error tracing positively influence clinical parameter assessments.

#### Summary of Lazy Luna’s capabilities

For SAX cine, atria assessment, T1, T2 mapping and LGE, LL offers: calculations of annotation (e.g. segmentation) metrics, clinical parameters and their differences, statistical summaries of reader differences (i.e. bias, standard deviation of differences, etc.). LL assesses the comparability of two readers via tolerance ranges. LL offers visualizations of differences, including annotation differences, statistical plots (e.g. paired boxplots, Bland-Altman, quantile-quantile plots), and error tracing, i.e. the tracing of clinical parameter differences to annotation differences. By generating reports of reader comparisons, including statistical information, qualitative differences and qualitative explanations of quantitative differences (via error tracing), LL exports summaries of reader comparisons.

### Software capabilities and application tests

#### Software capabilities

As software with the features listed in “Software features of Lazy Luna”, we hypothesize that LL is capable of supporting several QA tasks in CMR:

1. LL is capable of supporting the identification of quantitative outliers for several CMR imaging modalities and allows for identifying their causes.
2. LL is capable of supporting the assessment of AI methods by identifying intolerable parameter biases, locating frequent error sources and identifying segmentation failures that produced the intolerable biases.
3. LL is capable of supporting the instruction of trainees by identifying intolerable parameter differences and offering qualitative explanations of deviations.

#### Quality assurance application tests

In the following we formalize three typical QA application cases in CMR to test LLs software capabilities. The cohorts are described in Table 1.

**Table 1:** Cohort characteristics

QA Task	Image Types	# Cases	Population	Acquisition	Age	Sex (M/F)
**Reader Comparison**	SAX T1	12	Healthy volunteers	1.5T	26 ± 5	5/7
	SAX T2	12	Healthy volunteers	1.5T	26 ± 5	5/7
	SAX LGE	8	Patients with chronic myocardial infarction or myocarditis	1.5T LGE PSIR	57 ± 10	6/2
	LAX Cine (2CV, 4CV)	30	Clinical routine	1.5T LAX cine (2CV, 4CV)	61 ± 14	20/10
**AI Evaluation**	SAX Cine	144	Mixed cohort with healthy volunteers and patients with: IBD, muscular dystrophy, HCM, atrial fibrillation, psoriasis	1.5T/3T SAX cine	51 ± 18	80/64
**Trainee Evaluation**	SAX Cine, LAX Cine (2CV, 4CV), SAX T1 & T2	10	Mixed cohort, healthy volunteers, HCM, DCM, diffuse fibrosis	1.5T/3T multi-sequence protocol	55 ± 11	6/4

Legend: QA: quality assurance, AI: artificial intelligence, SAX: short-axis, LAX: long-axis, LGE: late gadolinium enhancement, PSIR: phase-sensitive inversion recovery, IBD: inflammatory bowel disease, HCM: hypertrophic cardiomyopathy, DCM: dilated cardiomyopathy

#### Outlier detection

##### Task and reader scenario

We investigated LL's capability to perform inter- and intraobserver comparisons and identify outliers on multiple image types, including SAX LGE (8 cases), SAX T1 (12 cases), SAX T2 (12 cases) and LAX cine (30 cases). The LGE studies were annotated by two expert readers and described in Fenski et al. [26]. For LGE LL calculated the left-ventricular volume (LVV), the left-ventricular myocardial mass (LVM), the scar mass (SCARM) and the scar fraction (SCARF). For mapping (T1 and T2) the average voxel intensity within the myocardial segmentations is calculated (GLOBAL_T1, GLOBAL_T2). For LAX cine images in 2- and 4-chamber view (2CV, 4CV), LL calculated end-systolic and -diastolic (ES, ED) atrial areas (2CVLAESArea, 2CVLAEDArea, 4CVLAESArea, 4CVLAEDArea, 4CVRAESArea, 4CVRAEDArea). To assess segmentation reproducibility Dice similarity coefficients (Dice) and Hausdorff distances (HD) were calculated.

##### Outputs

- Quantification and visualization of reader biases on clinical parameters
- Quantification and visualization of segmentation reproducibility
- Outlier detection (identifying cases that exceed tolerance ranges)
- Causal origins of clinical parameter differences of outlier cases (annotation differences with large impacts)
- Report of “Simple Comparisons” summarizing the above as PDFs

#### AI evaluation

##### Task and reader scenario

LL was used to inspect a commercial AI product on 144 cases on SAX cine cases (Table 1). Cases were annotated by an expert with 5+ years of CMR experience, and confirmed/corrected by a supervisor with 20+ years of CMR experience. We investigated LL for its capability to provide clinical parameter biases, quantitative measures of annotation differences, outlier detection and implausible annotation choices and behavior. Evaluated clinical parameters include the left and right ventricular (LV, RV) end-systolic and -diastolic (ES, ED) volumes, LV and RV stroke volume and ejection fraction (SV, EF), as well as the left myocardial mass in ED and papillary muscle masses (PAPMU) in ES and ED. To assess segmentation reproducibility, Dice and HD values were calculated for LV and RV endocardial as well as myocardial (MYO) segmentations.

##### Outputs

- Quantification and visualization of reader biases on clinical parameters
- Outlier detection (identifying cases that exceed tolerance ranges)
- Statistical analysis of segmentation failures by cardiac geometry (LV/RV, basal/midventricular/apical) of clinical parameter differences of outlier cases (annotation differences with large impacts)
- Illustration of implausible annotation choices
- Report of “Extensive Comparisons” summarizing the above
- Recording of AI evaluation video (Additional File 2)

#### Trainee feedback

##### Task and reader scenario

An instruction session was designed by having a trainee annotate a fixed set of 10 cases, which were selected for closely mirroring clinical reality (Table 1). Cases were annotated by an expert reader with more than 5+ years of CMR experience, and consensus was reached with a reader with 20+ years of CMR experience. LL calculated reader biases, error tracing allowed for investigation of annotation reasons for the biases. Assessed clinical parameters mirror the above for the same sequences.

##### Outputs

- Trainee biases exceeding tolerance ranges
- Annotation differences, causing these biases
- Report of “Simple Comparisons” summarizing the above

#### Key findings

The findings across the QA application cases are summarized in a table.

## Results

The software was implemented with the features described in Software features of Lazy Luna. Results focusses on three QA application cases.

### Outlier detection

**Fig. 4:**
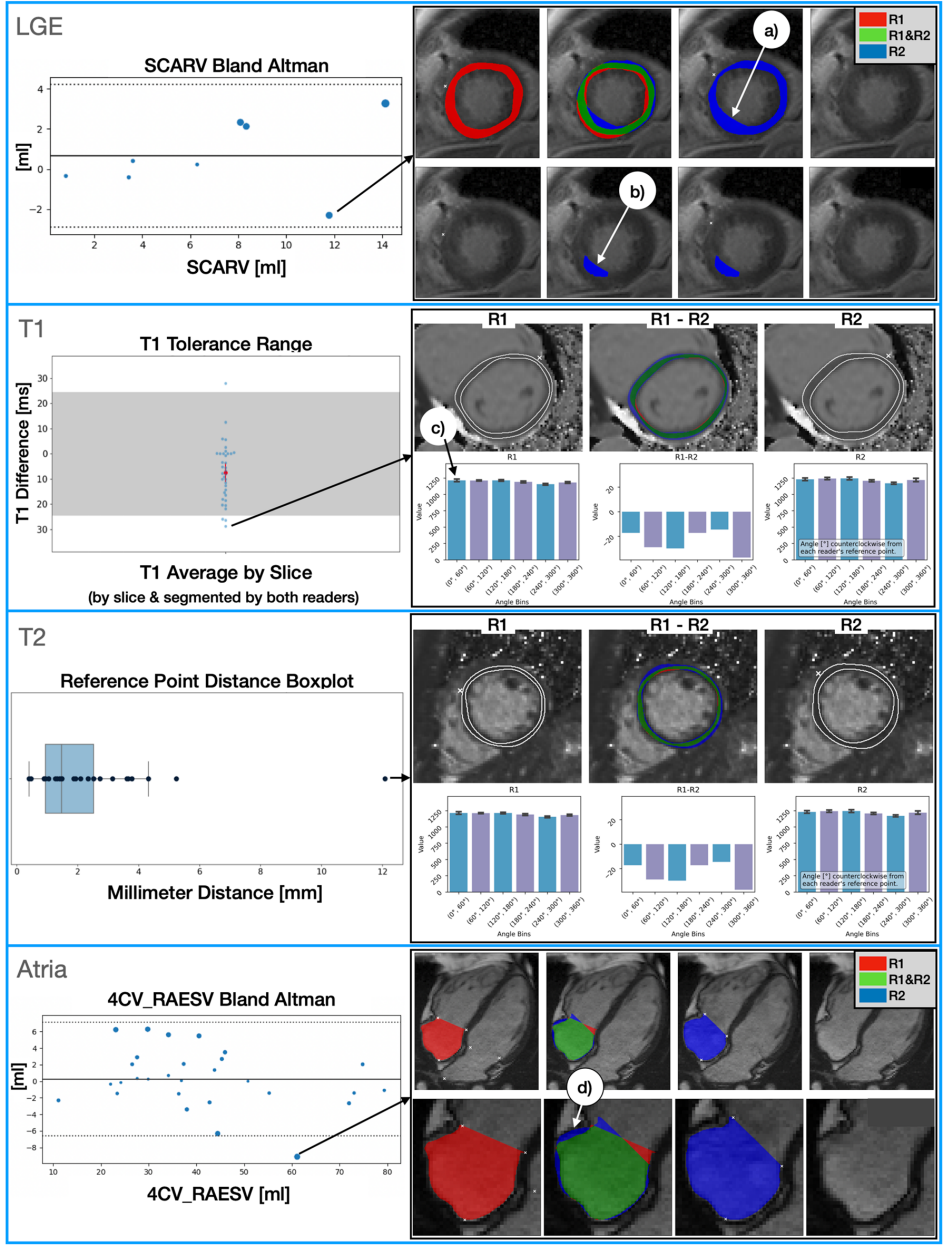
Outlier detection and error tracing (best viewed on monitor). Error tracing is illustrated for four imaging sequences in their respective panels (red = first reader (R1), blue = second reader (R2), green = agreement). The first panel (LGE) presents the second largest outlier for assessed scar volume in a bland altman plot. This difference was caused by different segmentation decisions for the LVM, with R1 considering the hyperintense voxels to be blood, and R2 considering them to depict scar tissue in the myocardium (**a**). Consequently, R1 assessed the focal bright spot as blood pool, R2 as scar tissue (**b**). The second panel (T1) presents an outlier of a single SAX T1 slice, outside of the tolerance range (grey bar). R2 produced a less conservative myocardial segmentation, which in turn integrated lighter voxels into the calculation. Below, three histograms for six segments are shown; from left to right: R1, average differences between R1 and R2, and R2. The reader-specific histograms show segmental averages as height and standard deviations as error bars (**c**) to provide an estimate of each segment value’s stability. The third panel (T2) shows a reference point distance selection of 12 mm. A wider myocardium segmentation of R2 produced segment value differences. The last panel (atria) shows an investigation of the largest right atrial volume difference between both readers in a bland altman plot. Segmentation differences in the RA cavity caused the difference (**d**). Considering the small segmentation differences (Dice = 93%), the large volume difference originates from the ellipsoidal calculation of volume. Legend: R1: first reader, R2: second reader, RA: right atrium, LGE: late gadolinium enhancement, SAX: short-axis, mm: millimeter, ml: milliliter, LVM: left ventricular myocardium, SCARV: scar volume, 4CV: 4 chamber view, RAESV: right atrial end-systolic volume, dice: dice similarity coefficient

Average clinical parameter differences and standard deviations as well as average segmentation metrics are shown in Table 2. Representative outliers were investigated from statistical plots and are presented in Fig. 4.

**Table 2:** Clinical parameters and segmentation metrics for outlier detection scenario

Parameter [unit]	Difference (mean ± std)	Contour Type	Dice [%]	HD [mm]
**LGE**				
**LV Volume [ml]**	−4.6±7.4			
**Myocardial Mass [g]**	−5.1±3.7	**Myo**	79	2.5
**Scar Mass [g]**	0.7±1.8	**Scar**	69	13
**Scar Fraction [%]**	1.3±1.7			
**T1**				
**Global T1 [ms]**	−7.5±7.6	**Myo**	81	1
**T2**				
**Global T2 [ms]**	−0.4±0.3	**Myo**	79	1
**Atria 4CV**				
**RAES Area [cm**^**2**^]	0±0.8	**RA (4CV)**	96	3
**RAED Area [cm**^**2**^]	0.1±0.7			
**RAESV [ml]**	0.3±3.4			
**RAEDV [ml]**	1±4.3			
**LAES Area [cm**^**2**^]	0±0.9	**LA (4CV)**	95	3
**LAED Area [cm**^**2**^]	−0.1±0.9			
**LAESV [ml]**	0.4±3.5			
**LAEDV [ml]**	−0.8±5.4			
**Atria 2CV**				
**LAES Area [cm**^**2**^]	0.3±0.7	**LA (2CV)**	96	4
**LAED Area [cm**^**2**^]	0±0.7			
**LAESV [ml]**	0.7±3.1			
**LAEDV [ml]**	−0.1±4.1			

Caption: The first column divides the table into five sections for different imaging sequences: LGE, T1, T2, 4CV Atria and 2CV Atria, as well as clinical parameters and units of measurement. The second column presents the reader differences as averages ± standard deviations. The third column lists the contour types, which affect the respective parameters. Columns four and five present the contour type’s average Dice similarity coefficients and Hausdorff distance

Legend: LV: left ventricle, RA: right atrium, LA: left atrium, ES: end-systole, ED: end-diastole, ESV: ES volume, EDV: ED volume, Myo: myocardium, Dice: Dice similarity coefficient, HD: Hausdorff distance

#### LGE

An interobserver comparison of LGE clinical parameters was computed with LL (Table 2, Fig. 4). The second reader estimated larger LV volumes, smaller myocardial- and scar masses as well as smaller scar fractions (Table 2). Segmentation inspection revealed that LV volume differences originated from slight oversegmentations of the LV cavity (Additional File 3). These oversegmentations reached into the myocardium, which decreased the myocardial and scar segmentations. Overall, the reproducibility of myocardial segmentations was higher than that of scar segmentations (Table 2).

Outliers: Case inspection revealed that determining the myocardial segmentation interfered with scar segmentation near the blood pool, which was interpreted as scar or blood pool, depending on the reader (Fig. 4 - LGE).

#### T1 and T2 mapping

Global T1 differences showed the second reader estimating higher T1 values than the first (Table 2). However, the reader bias (−7.5 ms) remained within the tolerance range of 24.5 ms with a high average segmentation similarity. Global T2 differences were small; both the reader bias and the standard deviation were below 1 ms with (Table 2). Differences between segmentation were small, but voxel values on the segmentation edges varied strongly, which led to single outliers.

Outliers: While investigating slices with T1 deviations outside of the tolerance range (Additional File 4), we found segmentations with inadequate margins to the myocardial boundary. Fat and blood pool intensities increased the AHA segment value differences in Fig. 4 - T1. For T2 we investigated a case in which the reference point distance between both readers was large. This affected the AHA segment value differences (Additional File 5).

#### Atria

Atrial volume, area and segmentation differences were small, regardless of atrium or view ( < 0.3 cm^2^, Table 2, Fig. 4).

Outliers: Although area differences were small, volume difference could be large (Fig. 4). One outlier case involved a missing segmentation (Additional File 6).

### AI evaluation

#### Clinical parameters

For the 144 cases Clinical value comparisons (mean difference ± standard deviation) and their acceptability are presented in Table 3 (also available in Additional File 1 and as video analysis in Additional File 2).

**Table 3:** Clinical parameter average differences

Clinical Parameter	Mean Difference ± Std	Acceptable
**LVESV [ml]**	−3.1 ± 6.6	(−4.2, −2.0), ±7.3
**LVEDV [ml]**	2.1 ± 7.0	(1.0, 3.2), ±10.8
**LVSV [ml]**	6.5 ± 8.3	(5.2, 7.9), ±4.5
**LVEF [%]**	3.0 ± 4.0	(2.4, 3.7), ±5.3
**RVESV [ml]**	0.3 ± 8.5	(−1.0, 1.7), ±9.7
**RVEDV [ml]**	−3.8 ± 9.8	(−5.4, −2.2), ±14.6
**RVSV [ml]**	−4.2 ± 10.3	(−5.9, −2.5), ±13.2
**RVEF [%]**	−1.4 ± 4.6	(−2.1, −0.6), ±5.5
**LVM [g]**	−2.0 ± 11.4	(−3.9, −0.2), ±13.3
**PAPMU ES [g]**	−0.2 ± 2.5	(−0.6, 0.2)
**PAPMU ED [g]**	1.2 ± 2.2	(0.9, 1.6)

Caption: In the first column the table presents the clinical parameter name. The second column presents the mean ± standard deviation of the Expert - AI parameter assessment difference. The third column references the acceptability of the artificial intelligence bias. This is calculated by calculating the 95% confidence interval, shown in parentheses and evaluating whether it is within the tolerance range, shown as ±tolerance range if available.

Legend: LV: left ventricle, ESV: end-systolic volume, EDV: end-diastolic volume, EF: ejection fraction, RV: right ventricle, LVM: left ventricular myocardium, PAPMU: papillary muscle mass, AI: artificial intelligence

**Fig. 5:**
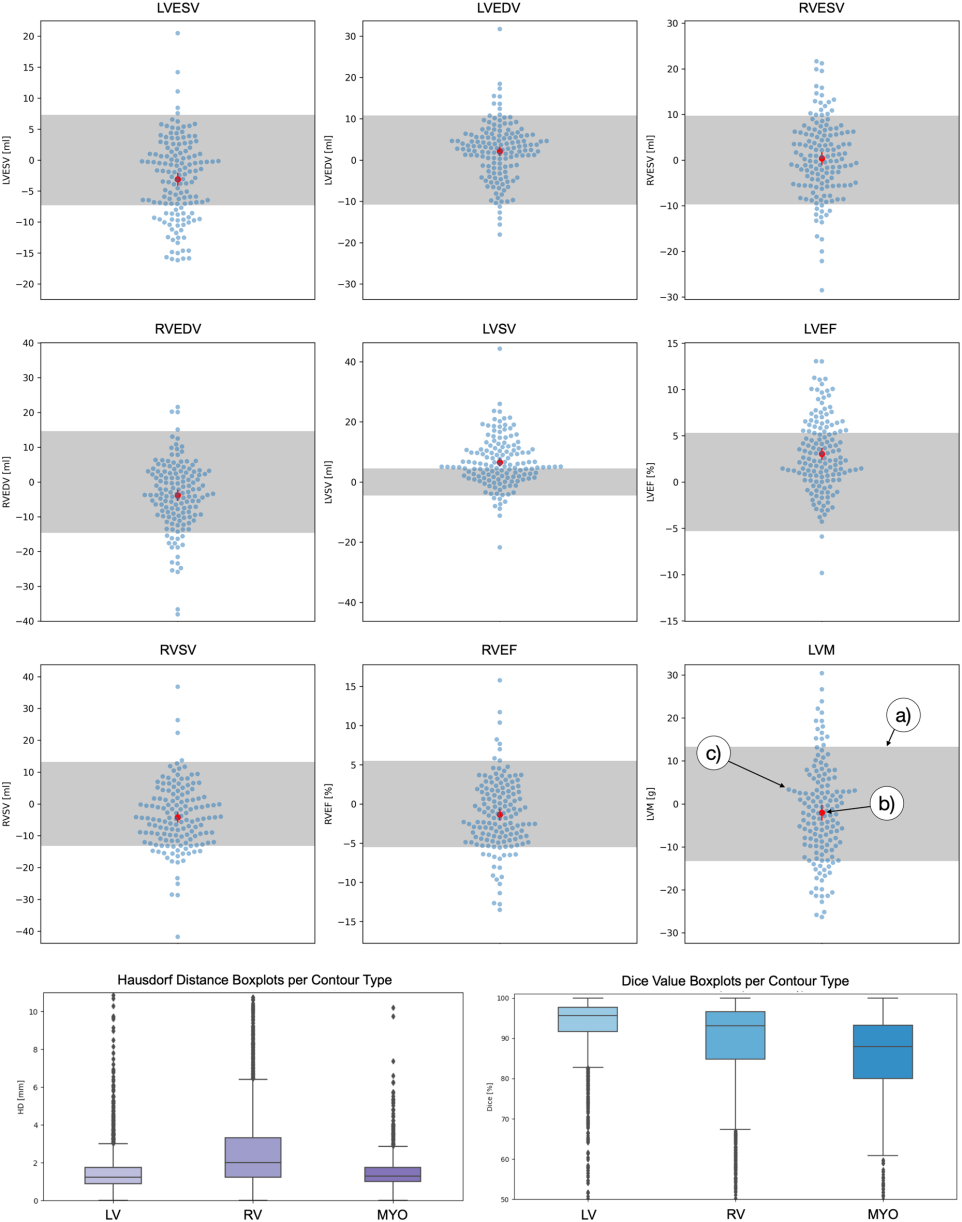
Clinical parameter and segmentation metric statistics for expert vs. AI comparison. The first three rows show tolerance range plots for clinical parameter differences between the expert readers and the AI method. The parameter’s tolerance range is plotted as a grey bar (**a**); the confidence interval for the two readers is a red line passing through the average difference (**b**), a red circle in the confidence interval’s middle. All case differences are plotted as light blue dots on top as swarm-plots such that dots with similar y-values do not overlap and hide each other (**c**). In row four HD boxplots are plotted subdivided by segmentation type on the left, and corresponding dice boxplots on the right. Boxplots show the central 50% of the values within the box, the median as a horizontal line within the box, whiskers delineate where 98% of the values lie. Outliers are plotted as points beyond the whiskers. Legend: LV: left ventricle, RV: right ventricle, ESV: end-systolic volume, EDV: end-diastolic volume, SV: stroke volume, EF: ejection fraction, LVM: left ventricular myocardial mass, MYO: myocardium, HD: Hausdorff distance, dice: dice similarity coefficient

AI biases were evaluated against confidence intervals and tolerance ranges (Table 3, Fig. 5). For SAX cine images the calculated LV stroke volume confidence interval (confidence interval: (5.2 ml, 7.9 ml)) lies outside of the tolerance range (±4.5 ml). While neither the LVESV nor the LVEDV exceed the tolerance ranges, their biases point in different directions, which may cross the SV tolerance range in summation (Fig. 5).

#### Segmentation reproducibility and geometry

Segmentation agreement was high overall, though larger deviations were observed in the RV (Fig. 5). A further decomposition of these segmentation differences to their cardiac positions was performed in Table 4. The table uses two Dice metric definitions, one for all slices annotated by at least one reader (called all slices), and one for slices that were segmented by both readers. Overall, midventricular segmentations had high dice values around 90%, low HD values with small impacts on volumetric differences. However, segmentation quality decreased in basal and apical slices and segmentation deviations were generally higher in RV and MYO than in the LV. The myocardial dice metric for the basal slices improved drastically (from 20% to 86%) when only slices were considered that were segmented by both readers.

**Table 4:** Metrics by cardiac location and contour type

Metric	LV	MYO	RV
**Basal Slices**			
Dice (all slices) [%]	90	20	62
Dice (slices segmented by both) [%]	94	88	73
HD [mm]	5	3	16
Abs. ml diff. (per slice) [ml]	1.3	5.5	2.5
**Midventricular Slices**			
Dice (all slices) [%]	94	86	92
Dice (slices segmented by both) [%]	94	88	92
HD [mm]	3	3	6
Abs. ml diff. (per slice) [ml]	0.6	1.2	0.9
**Apical Slices**			
Dice (all slices) [%]	70	63	66
Dice (slices segmented by both) [%]	80	69	74
HD [mm]	3	4	6
Abs. ml diff. (per slice) [ml]	0.4	0.9	0.6

Caption: The columns are metric name, left ventricular endocardial segmentation, myocardial segmentation, right ventricular endocardial segmentation. The table’s rows are divided into three sections referring to the basal slices (the most upper slice as defined by the expert and all slices above), midventricular slices (all slices between base and apex) and apical slices (the lowest slice as defined by the expert and all slices below). For each section and each contour type, the dice values for all slices annotated by at least one reader, the dice values for slices annotated by both readers, the Hausdorff distance and the absolute milliliter difference per slice are presented

Legend: LV: left ventricular endocardial segmentation, MYO: myocardial segmentation, RV: right ventricular endocardial segmentation, Dice: Dice similarity coefficient, HD: Hausdorff distance, Abs. ml diff: absolute milliliter difference

#### Error cases and visual examples

**Fig. 6:**
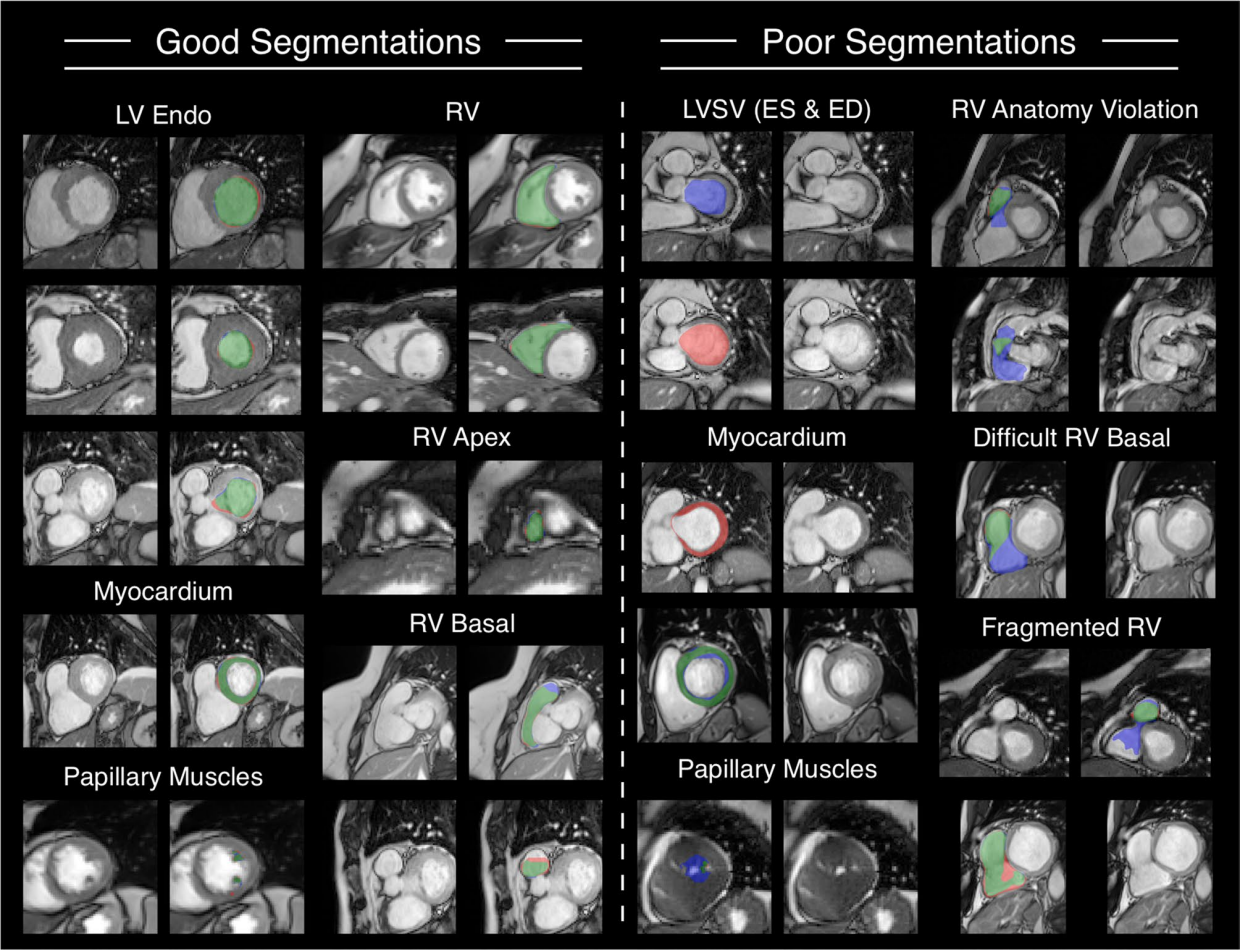
Good and bad segmentation examples (best viewed on monitor). Red: only expert, blue: only AI, Green: agreement. Examples of good segmentations are on the left (per subfigure: base image left, segmentation comparison right). Examples of poor AI segmentations are on the right half, demonstrating multiple causes for segmentation mistakes, from difficult slices, due to blurriness, mistakes and anatomical constraints, which are more apparent in the moving cine than the singular image. Legend: AI: artificial intelligence, LV: left ventricle, RV: right ventricle, Endo: endocardium, ES: end-systole, ED: end-diastole, SV: stroke volume

The Dice deviation for myocardial segmentations in Table 4 indicates that basal slices were often overlooked or unjustifiably segmented (Fig. 6 – Poor Segmentations – Myocardium). Nonetheless, myocardial mass differences remained within the tolerance range (Fig. 5); overlooked myocardial segmentations in basal slices were volumetrically compensated for by midventricular oversegmentations into the LV blood pool (Fig. 6 – Poor Segmentations – Myocardium). LV endocardial segmentations had high dice metric averages, and low average milliliter impacts regardless of cardiac location (Table 4, Fig. 6 – Good Segmentations – LV Endo). However, large LVSV differences between expert and AI resulted from juxtaposed LV endocardial basal slice decisions; for one such case both annotations are shown for both the ES and ED phase (Fig. 6 – Poor Segmentations – LVSV (ES & ED). Further examples are provided in the appendix (see Additional File 1). On pages 14 and 15, two additional cases of ES and ED basal slice decision failures are shown: one involving a difficult ES decision due to present but insufficient myocardium, and the other showing a missed thin basal myocardium in ED. A similar issue is illustrated on pages 16 and 17, where a motion artefact further reduced image quality and contributed to the misinterpretation. The RV was more problematic in the basal slice revealing an annotation decision, which was more difficult than for the LV endocardial segmentation; high basal HDs reflected the problem of deciding what image areas belonged to the RV (Fig. 6 – Poor Segmentations – Difficult RV Basal, Fragmented RV). Occasionally, RV segmentations spilled over plausible anatomical boundaries (Fig. 6 – Poor Segmentations – RV Anatomy Violation, Difficult RV Basal, Fragmented RV).

### Trainee feedback

**Fig. 7:**
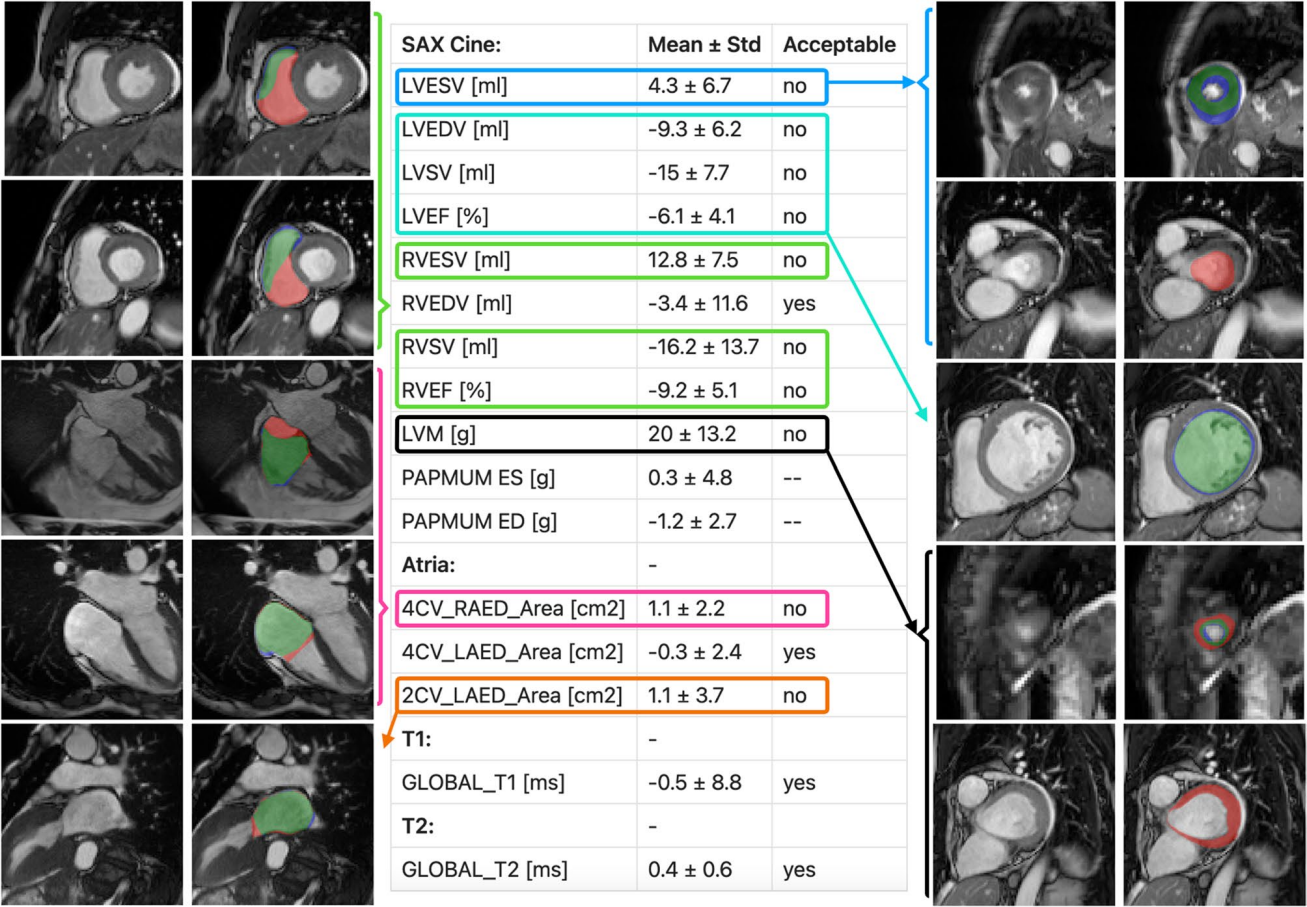
Trainee assessment - mean differences and example segmentation comparisons. The figure presents a table surrounded by segmentation examples for CMR images. For segmentation examples the reader disagreement was color-coded as: red: expert reader, blue: trainee, green: agreement. The table consists of three columns, clinical parameter names, mean difference ± standard deviation of the clinical parameter and whether expert and trainee have equivalent means (whether the trainee is inside a predefined tolerance range). Sequence types (SAX cine, LAX cine, T1 and T2) divide the rows into sections. For all clinical parameters in which the expert and the trainee were not equivalent, examples that caused the statistical differences are presented. Legend: LV: left ventricle, RV: right ventricle, ESV: end-systolic volume, EDV: end-diastolic volume, SV: stroke volume, EF: ejection fraction, LVM: left ventricular myocardium, PAPMU: papillary muscle mass, LA: left atrium, RA: right atrium, 2CV: two chamber view, 4CV: four chamber view

The differences between the expert reader and the trainee were assessed as the clinical parameter value assessed by the expert minus the value assessed by the trainee (Fig. 7). For SAX Cine the trainee underestimated LVESV; papillary muscles were included in the myocardium in ES for thick myocardia and basal slices were overlooked in two cases (Fig. 7 - dark blue). The LVEDV, LVSV and LVEF were overestimated; the trainee significantly oversegmented the endocardium into the myocardium in most slices, which added up to the volume disparities (Fig. 7 - light blue). Volume and percentage differences exceeding the tolerance ranges for the trainee were the RVESV, RVSV and RVEF; basal slices were misinterpreted as being divided into ventricle and atrium, leading the trainee to exclude the supposed atrial volume (Fig. 7 - green). The LVM was underestimated because of thin myocardial segmentations in most cases (including the two largest outliers), and by overlooked basal slice myocardia in two cases (Fig. 7 - black). The trainee underestimated the RAED area in the 4CV; this was due to disagreement on the delineation of the valves as well as a misinterpretation of an intruding aorta, which was excluded by the trainee (Fig. 7 - pink). The LA was underestimated in 2CV because of valvular delineation disagreements (Fig. 7 - orange). Atria evaluation excluded end-systolic clinical parameters, since the trainee exclusively segmented the ED phase. For mapping (T1 as well as T2) the trainee was within the tolerance ranges. A report of the trainee analyses was exported (Additional File 7).

### Key findings

The findings of the three QA scenarios are summarized in Table 5.

**Table 5:** Key findings across quality assurance scenarios

QA Scenario	Image Types & Sequences	Key LL Features Utilized	Key Findings
1. Outlier Detection	LGE (SAX), T1/T2 maps (SAX), Cine (2CV, 4CV LAX)	Tolerance range-based outlier detection; Dice/HD segmentation metrics; error tracing; Simple PDF reports	LGE biases driven by blood pool vs scar misinterpretation; T1 outliers linked to border voxels; large volume outliers in atria despite minor area differences due to ellipsoidal estimation; error tracing exposed regional segmentation causes.
2. AI Evaluation	Cine (SAX)	Clinical bias evaluation with confidence intervals vs. tolerance ranges; segmentation metrics (Dice/HD by slice); anatomical plausibility checks; Extensive PDF report; structured video analysis	LVSV bias exceeded tolerance range despite volume biases being within limits; segmentation failures more frequent in basal/apical slices, especially for RV; error tracing revealed fragmented or anatomically implausible contours in failure cases; papillary muscle mass inconsistently handled.
3. Trainee Feedback	Cine (SAX, LAX), T1/T2 maps	Bias detection via tolerance ranges; error tracing for cause explanation; instructional examples; Simple PDF reports	Trainee oversegmented LV in ED and underestimated ES due to misinterpretation of myocardium; RV basal slices poorly handled due to atrial/ventricular boundary confusion; accurate mapping values; atrial segmentation discrepancies driven by valve and vessel confusion.

Legend: AI: Artificial intelligence, LGE: late gadolinium enhancement, SAX: short-axis, LAX: long-axis, HD: Hausdorff distance, Dice: Dice similarity coefficient, ES: end-systole, ED: end-diastole

## Discussion

This paper’s main result is that a wide range of CMR quality assurance needs can be met by the semi-automated QA tool, Lazy Luna (available via https://github.com/thadler/LazyLuna). We used this tool to support outlier detection, AI evaluation and a trainee performance assessment. Beyond quantifying and visualizing annotation differences, calculating clinical parameter differences and revealing reader biases, Lazy Luna offered qualitative explanations for reader differences with a software feature called error tracing. In the “Outlier Detection Application Case” LL allowed for identifying clinical parameter differences and presenting explanatory segmentation differences, giving credence to software capability 1. The “AI Evaluation” with LL illuminated typical segmentation choice differences, their cardiac locations and volumetric effects. Tolerance ranges allowed for determining that the left ventricular volumes themselves were acceptable, however deviated in juxtaposed directions for ES and ED thus making the stroke volume inacceptable. LL calculated the frequency of segmentation difficulties and their impacts, and allowed for the illustration of typical errors. These results support the functionality of software capability 2. The “Trainee Feedback” application case identified clinical parameters, which were outside of acceptable tolerance ranges and visualized segmentation differences, supporting the functionality of software capability 3. Lazy Luna is currently the only available software tool that offers multi-level reader comparison between readers for CMR modalities. Recently, building and extending upon Lazy Luna, Lumos was implemented [27]. Lumos formalizes a multi-reader comparison approach, in which multiple different thresholding methods for LGE were compared to each other.

### Segmentation difficulties and standardization options

For the application cases, error tracing exposed vastly different segmentation choices and behavior having disparate impacts on quantitative parameter differences between readers. Some segmentation differences implied conflicting interpretations of cardiac structures within the images. Among the LGE outliers, there were intermingling segmentation difficulties in the tradeoff between more or less conservative myocardial segmentations and scar extent. In short-axis stacks, large clinical parameter differences typically originated from difficult basal slice decisions. On the one hand, these decision differences were often produced on images with partial volume effects, which presented different cardiac anatomy due to the image’s slice thickness. On the other hand, other differences frequently implied contouring technique differences, such as selecting more or less papillary muscles or segmenting the myocardium slimly or thickly to avoid fat and blood pool voxels from entering clinical parameter calculations. The utility of error tracing is enhanced with dedicated tables in which statistics on annotation metrics and their impacts on clinical parameters are shown. For the AI evaluation application case this is shown in Table 3, in which Dice and HD are first differentiated by contour type and cardiac location (basal, midventricular and apical) and then presented in context of their volume differences. This revealed that differences group within the basal slices, which was further investigated with the help of error tracing and qualitative examples. In practice error tracing is used to find meaningful annotation failures and differences in the vast array of all annotations from the assessed clinical parameter differences, when investigators are interested in finding their causal origin. However, a thorough inspection into the efficacy of this method is warranted.

The statistical and visual tool used for error-tracing in LL are tolerance ranges, which decide the acceptability of reader deviations by measuring whether their bias is securely captured by intrareader variability. While this approach identifies statistically relevant differences, they do not inherently reflect clinical impact on a per-patient basis. For patients with parameters near clinical thresholds, small deviations can affect decisions, for instance, an LV EF near the 35% cut-off for ICD eligibility. Inversely, large deviations may be of negligible clinical consequence when they occur in ranges far from clinical decision thresholds. Therefore, while tolerance ranges offer a quantitative baseline for quality assurance by limiting deviations to intrareader variability, they must be interpreted within the clinical context. Future studies could assess whether deviations identified by LL correspond to actual changes in clinical decision-making, further bridging the gap between statistical quality control and clinical significance.

Depending on the nature of consistent difficulties, the differences may imply natural limits to the effectiveness of standardization or they may imply that current standardization lacks rigorousness. Whereas images that are difficult to visually interpret may need to be addressed earlier in the CMR imaging pipeline, contouring techniques may be synchronized for more reproducible post-processing. The relevance and impact of demographic factors and diseases should also be considered in QA studies. For example, patients with hypertrophic cardiomyopathy typically have thicker myocardia, which may disproportionately impact parameter differences even when contour differences are comparable. The annotation of papillary muscles in LV quantification has been shown to significantly impact volumetric and functional parameters, particularly in patients with hypertrophic cardiomyopathy [28, 29]. Consistency in PAPMU handling is essential, as differences in inclusion or exclusion can produce systematic biases even when segmentations are otherwise clean.

### Integrated quality assurance in post-processing

Comparing readers to each other can support important QA tasks. However, as differences are investigated and clarified, the differences themselves become an exploitable source of data. Gathering parameter and annotation differences as well as understanding reasons for annotation differences between experts and beginners or AIs may provide the foundation for integrating new QA procedures into the post-processing pipeline.

In recent years several methods were developed for segmentation quality estimation, such as Monte-Carlo Dropout, reverse classification accuracy, linear combinations of ensemble Dice estimations and Bayesian networks [30–33]. At the same time integrating cardiac geometry constraints on models has been explored as shape-priors for the loss function during training time, as shape-constraints on viable geometries, as post-hoc segmentation replacements with segmentations of similar hearts (similarity determined via embeddings) as well as quantitative plausibility checks on volumes over all phases [34–37]. Whether for AIs or human readers, consistency checks and improvement suggestions have the potential to increase the cardiac plausibility of segmentations, and catch errors on the fly in clinical routine. LL does not perform these QA tasks itself, but it provides an effective platform to benchmark such methods. A regulatory proposal suggested the monitoring of AIs as an integral aspect of AI medical device deployment [38]. The future of CMR may see benchmarking the QA-elements of AIs as a central data science challenge.

### Clean and heterogeneous data as essential ingredients to CMR quality assurance

In the last decade CMR has identified heterogeneous data as crucial to address multiple confounders, such as: different diseases, vendors, field strengths, imaging and reconstruction techniques as well as different sites and readers. For clinicians, heterogeneity of diseases is foundational, as it is the starting point of disease diagnosis and the origin of healthy reference values in CMR [39]. In recent years, interest in data heterogeneity has been reinvigorated because confounders interfere with the communicability of normal values. Such difficulties have incentivized investigations into deviations between sites [20, 40] and workarounds like the Z-score normalization of parameters across sites [41]. For AI-developers dataset size and heterogeneity has increased in importance with the advent of convolutional neural networks [3]. As AIs need to perform excellent automatic segmentations that generalize over vendors, sites and diseases, challenges and datasets have evolved from concerning the midventricular LV slices (LVQuant19), over full bi-ventricular quantification (ACDC) to multi-vendor, multi-site and multi-disease bi-ventricular quantification challenges (M&M, UKB CMR) to evermore mirror clinical reality [6, 42–44]. This progression of CMR segmentation challenges towards real-world data is good machine learning practice [45] and is in accordance with the intentions of recent regulatory action plans [46]. Nonetheless, closing the gap on clinical reality may remain difficult; a recent study even raised the question of unknown confounders causing mid- to long-term parameter differences [47].

Building on the importance of image heterogeneity, inter-vendor domain shifts have become a key challenge in translating AI segmentation methods into diverse clinical environments. Beyond vendor differences, variations in patient anatomy, image quality, and acquisition conditions can also act as domain shifts. In our evaluation, several cases of large LVSV errors highlighted the underlying limitations of current training strategies. For example, as shown in Additional File 1 (p. 14–15), oversegmentation in ES and a missed basal slice in ED appear to result from different causes: a challenging ES anatomy with minimal myocardium, and an unusually thin ED basal slice. In another case (p. 16–17), the pattern is reversed, but the likely cause is severe motion artefact, which degraded image quality. Other examples (p. 18–19) show good basal segmentation, but small inaccuracies at the endocardial boundary accumulated across multiple slices, suggesting challenges in detecting subtle boundary gradients.

These observations suggest that AI performance could be improved by incorporating targeted training augmentation, with special emphasis on difficult basal anatomy, low-contrast boundaries, and artefact-prone images. Data curation strategies that ensure representation of rare but clinically relevant case diversity – including motion-affected scans and thin myocardial walls – may help reduce such errors in the future. Full et al. showed that even relatively small incorporations of a new domain’s data may suffice to ensure an AI method’s generalizability [48]. Other approaches have focussed on mimicking potential domain shifts and artefacts by applying extensive image augmentations during AI training [49, 50]. These augmentation approaches have proven effective at addressing the domain shift issues in a wide array of medical imaging applications, including biventricular segmentation in CMR [51]. While augmentation strategies address domain shift, curating training datasets that capture anatomical variability – such as difficult basal slices, thin myocardial walls, and motion-degraded images – remains essential for improving segmentation robustness.

Provided a sufficiently heterogeneous dataset, excellent annotations remain as a key component to effective QA. This is true for training AIs to perform expert annotations, but is especially true when evaluating readers (humans and AIs) against expert annotations on a benchmark-dataset. Relevant annotation differences between expert readers remain omnipresent in all datasets [20] and are a problem as an interobserver bias inside the training and benchmarking datasets for AIs but also in illustrative and evaluation datasets for teaching purposes [52].

In recent AI training challenges, generating excellent annotations has been formalized as multi-actor procedures. For the so-called ACDC and M&M challenge [6, 43], “clinical segmentations were corrected by two in-house annotators that had to agree on the final result” [53]. Especially for large datasets with a multitude of sequence types, vendors, sites and pathologies, further automation of maximizing segmentation quality may provide benefits that reduce the time required to clean data. New software tools could provide semi-automated procedures that suggest probable mistakes to a supervising reader.

### Outlook on teaching in CMR

The Trainee Feedback application case revealed the trainee’s difficulty with RV segmentation choices leading to serious volumetric consequences. Whereas the RV tends to be the most difficult cardiac structure in the surrounding literature, the average difference can trend around zero when contouring technique is standardized. The trainee’s differences were primarily due to difficulties distinguishing basal from midventricular slices, which resulted in inappropriate RV contours (Fig. 7). Additional examples are shown in Additional File 7, including the exclusion of RV papillary muscles from the endocardial contour (p. 11), and a misinterpretation of the atrium as the RV cavity in a basal slice (p. 15). This type of targeted feedback helps trainees align image interpretation with group consensus and promotes adherence to established guidelines. This application case demonstrated LLs ability to identify inacceptable trainee biases (compared to an expert) and present segmentation differences, which led to the biases. As CMR availability grows exponentially in recommendations and availability around the globe, the clarification and teaching of segmentation techniques and post-processing guidelines grows more difficult and expensive in turn [2, 54]. Educating trainees in CMR and surrounding fields builds on curriculums and face-to-face teaching with immediate feedback [2]. In principle, large parts of trainee feedback can be automated or at least semi-automated. LL offers a semi-automated comparison between a trainee and an expert, calculating statistical biases on clinical parameters automatically while allowing an investigator to quickly find qualitative explanations for these biases. An extension of LL is conceivable in which this middleman investigator is replaced. If the individual segmentation differences were classified as belonging to a “typical kind of mistake”, teaching videos could be pre-recorded and presented to the trainee so that the trainee can improve independent from a teacher. As the trainee application case only consisted of 10 cases and one trainee, in the future, a thorough investigation into trainee improvements should be performed.

More broadly, LL could be integrated into clinical practice as a lightweight QA tool for onboarding new staff, conducting peer review of segmentations, or periodically auditing reader agreement—especially in multi-reader or AI-assisted environments. Because LL provides intuitive visual and quantitative feedback on where differences occur, it has potential to support not just education but also standardization efforts and continuous quality improvement in both research and clinical settings.

### Limitations

LL requires streamlined data for all investigations in order to make automated outputs easily understandable. In order to use LL as a semi-automated QA tool a dataset with high-quality annotations is necessary, which may be difficult to acquire. While LL currently operates retrospectively and relies on pre-existing segmentations, future integration with segmentation tools could enable real-time feedback, enhancing its utility for training and clinical workflows.

Qualitative descriptors such as ‘fragmentation’ were used to highlight anatomically implausible segmentations. However, these were not defined by formal metrics, and no quantitative classification was applied. Developing robust metrics to capture such errors remains an area for future work.

Several of the quality assurance scenarios (e.g. LGE: *N* = 8, Trainee Feedback: *N* = 10) involve small sample sizes, which limit statistical power and the ability to detect meaningful effects. These findings should be interpreted cautiously, as they may not generalize to broader populations. They are best considered exploratory, highlighting the utility of Lazy Luna, rather than providing definitive conclusions. Larger studies are needed to validate and extend these results.

## Conclusion

Lazy Luna supported three typical quality assurance tasks in CMR by offering a platform for effective reader comparison. Illuminating statistical parameter differences with their qualitative reasons provides a sound foundation for improvement opportunities. Future AI systems will integrate sophisticated QA techniques with anatomical guarantees in addition to excellent clinical parameters.

## Electronic supplementary material

Below is the link to the electronic supplementary material.

Supplementary Material 1

Supplementary Material 2

Supplementary Material 3

Supplementary Material 4

Supplementary Material 5

Supplementary Material 6

Supplementary Material 7

## Data Availability

The datasets analysed during the current study are not publicly available due to patient German data privacy laws. They are available from the corresponding author on reasonable request after communication with the legal department based on the EU law and the rules of the Berlin data officer. The datasets generated during this study are included in this published article and its supplementary information files.

## Abbreviations

AI: Artificial intelligence
CMR: Cardiovascular magnetic resonance
CNN: Convolutional neural network
CV: Chamber view
Dice: Dice similarity coefficient
ED: End-diastole
EDV: End-diastolic volume
EF: Ejection Fraction
ES: End-systole
ESV: End-systolic volume
HD: Hausdorff distance
LA: Left atrium
LAX: Long-axis
LL: Lazy Luna
LV: Left ventricle
LVM: Left ventricular myocardium
LVV: Left ventricular volume
MYO: Myocardium
PAPMU: Papillary muscles
PDF: Portable document format
QA: Quality assurance
RA: Right atrium
RV: Right ventricle
SAX: Short-axis
SCARM: Scar mass
SCARF: Scar fraction
SV: Stroke volume

## Declarations

### Software section

Project name: Lazy Luna.

Project home page: https://github.com/thadler/LazyLuna.

Archived version: None (available in Github repository).

Operating systems: platform independent. Tested on 64-Bit systems of macOS Mojave 10.14.6, Windows 10 Home and Ubuntu > 20.04.

Other requirements: Python > 3.8, Pydicom > 2.2.0, Numpy > 1.20.1, Shapely > 1.8.0, Rasterio > 1.2.10, Pandas > 1.2.4, PyQt6, Matplotlib > 3.3.4, Seaborn > 0.11.1.

Licence: MIT.

Any restrictions to use by non-academics: none.
