# Supplementary material for: A semi-automated quality assurance tool for cardiovascular magnetic resonance imaging: application to outlier detection, artificial intelligence evaluation and trainee feedback
Source: BMC Med Inform Decis Mak. 2025 Dec 2;25:437. doi: 10.1186/s12911-025-03271-6 (PMC12670863; doi:10.1186/s12911-025-03271-6)
Supplement: Supplementary file 1 — Supplementary Material 1 [file 12911_2025_3271_MOESM1_ESM.pdf]

Clinical Parameter Means and Tolerance Ranges

Clinical Result (mean±std)	Gold	Vendor_AI	Difference	±Tolerance range
LVESV [ml]	57.0 (19.1)	60.1 (19.0)	-3.1 (6.6)	(-4.2, -2.0), ±7.3
LVEDV [ml]	151.8 (34.9)	149.7 (34.4)	2.1 (7.0)	(1.0, 3.2), ±10.8
RVESV [ml]	77.7 (24.4)	77.4 (24.3)	0.3 (8.5)	(-1.0, 1.7), ±9.7
RVEDV [ml]	164.9 (41.4)	168.8 (42.9)	-3.8 (9.8)	(-5.4, -2.2), ±14.6
LVSV [ml]	95.1 (20.6)	88.6 (20.1)	6.5 (8.3)	(5.2, 7.9), ±4.5
LVEF [%]	61.3 (6.3)	58.2 (6.1)	3.0 (4.0)	(2.4, 3.7), ±5.3
RVSV [ml]	87.2 (22.0)	91.4 (22.5)	-4.2 (10.3)	(-5.9, -2.5), ±13.2
RVEF [%]	53.2 (6.5)	54.6 (6.0)	-1.4 (4.6)	(-2.1, -0.6), ±5.5
LVM [g]	93.6 (32.6)	95.6 (28.5)	-2.0 (11.4)	(-3.9, -0.2), ±13.3
RVM [g]	0.0 (0.0)	0.0 (0.0)	0.0 (0.0)	(0.0, 0.0), ±nan
LVESPAPMUM [g]	4.4 (1.7)	4.6 (2.8)	-0.2 (2.5)	(-0.6, 0.2), ±nan
LVEDPAPMUM [g]	4.8 (2.3)	3.6 (1.9)	1.2 (2.2)	(0.9, 1.6), ±nan
LVESP [#]	11.2 (1.7)	11.2 (1.8)	0.7 (0.9)	(0.6, 0.9), ±nan
RVESP [#]	11.6 (1.7)	12.0 (2.0)	0.9 (1.0)	(0.8, 1.1), ±nan
LVEDP [#]	27.0 (7.7)	20.7 (12.7)	0.5 (0.7)	(0.4, 0.6), ±nan
RVEDP [#]	27.0 (7.7)	23.8 (10.8)	0.7 (1.0)	(0.5, 0.8), ±nan
NrSlices [#]	16.7 (1.4)	16.7 (1.4)	0.0 (0.0)	(0.0, 0.0), ±nan

Table. 1 This table shows the clinical parameter names in the first column. The other columns show statistics concerning the parameters. The first and second readers' means (stds) are shown in the second and third column, respectively. The mean and std of the differences between both readers is presented in the fourth column. The mean difference of both readers ± 95% confidence intervals are shown in parentheses with ±tolerance ranges thereafter. This provides information on whether the 95% estimate of the mean difference between both readers is within an acceptable limit.

Tolerance range paper: Zange L, Muehlberg F, Blaszczyk E, Schwenke S, Traber J, Funk S, et al. Quantification in cardiovascular magnetic resonance: agreement of software from three different vendors on assessment of left ventricular function, 2D flow and parametric mapping. J Cardiovasc Magn Reson. 2019 Dec;21(1):12.

Clinical Results Differences

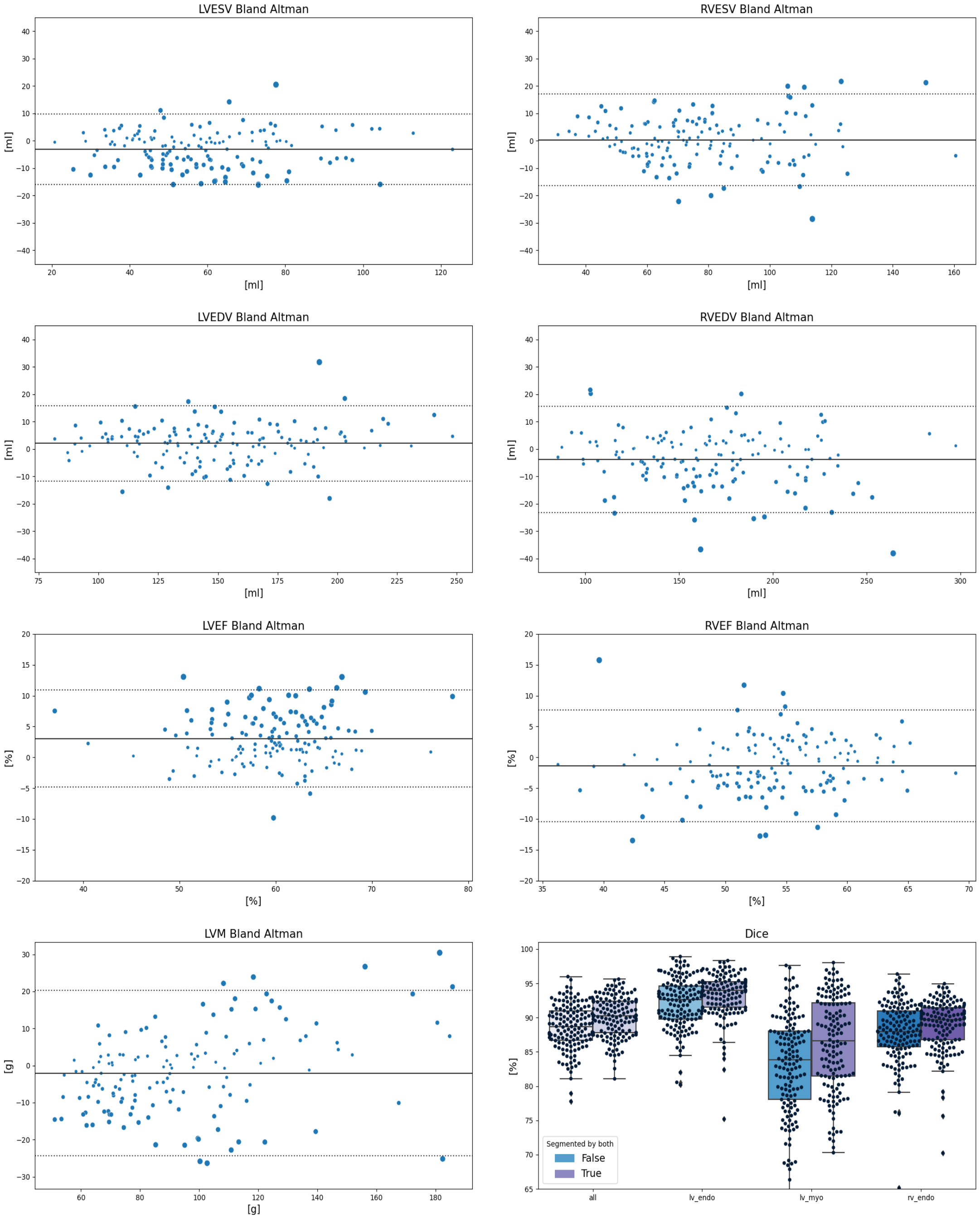

Fig. 1 Clinical Parameter Bland-Altmans: Bland-Altman plots show clinical parameter averages and differences as points for all cases. Point size represents difference, the solid line marks the mean difference between readers, the dashed lines mark the mean differences ±1.96 standard deviations. The last plot offers two Dice boxplots per contour type, one for all images, another restricted to images segmented by both readers. Legend: GUI: Graphical user interface, RV: Right ventricle, LV: Left ventricle, ESV: End-systolic volume, EDV: End-diastolic volume, EF: Ejection fraction, LVM: Left ventricular mass, Dice: Dice similarity coefficient

Confidence Intervals

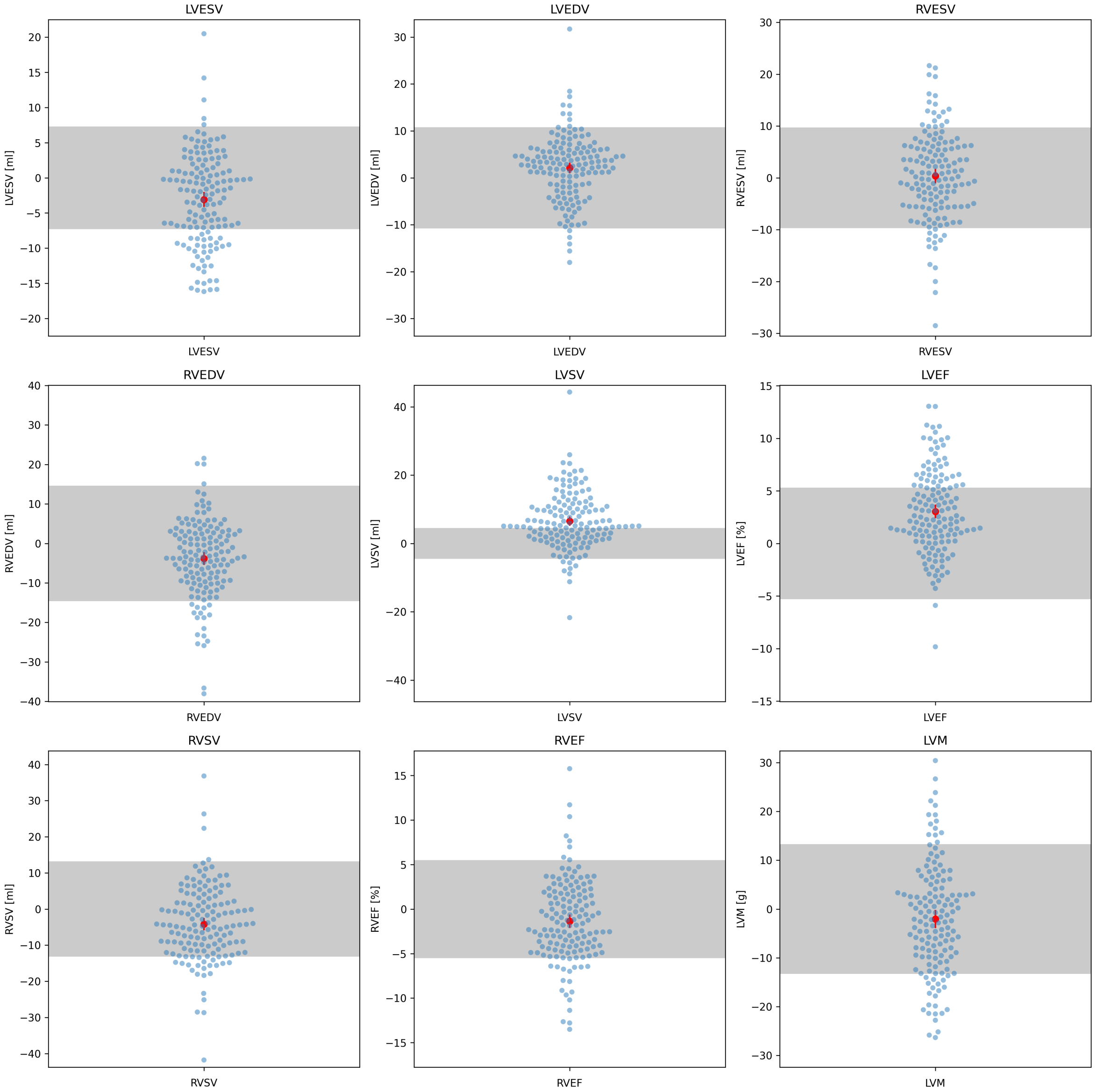

Fig. 2 Tolerance Ranges and Confidence Intervals: Each subfigure references a clinical parameter. Tolerance intervals are shown as gray bars and represent ±1.96 standard deviation of an expert intrareader deviation as derived in another publication (see below). The 95% confidence intervals of the mean value is represented as an error bar in red. Individual clinical parameter differences per case are plotted in blue. Legend: LV: Left ventricle, RV: Right ventricle, ESV: end-systolic volume, EDV: end-diastolic volume, EF: ejection fraction, LVM: Left ventricular myocardium.

Tolerance range paper: Zange L, Muehlberg F, Blaszczyk E, Schwenke S, Traber J, Funk S, et al. Quantification in cardiovascular magnetic resonance: agreement of software from three different vendors on assessment of left ventricular function, 2D flow and parametric mapping. J Cardiovasc Magn Reson. 2019 Dec;21(1):12.

Metrics by Cardiac Location and Contour Type

Metric	LV Endocardial Contour	LV Myocardial Contour	RV Endocardial Contour
**Basal Slices**			
Dice (all slices) [%]	89.58	19.56	62.40
Dice (slices contoured by both) [%]	94.14	87.63	72.97
HD [mm]	4.77	2.95	16.22
Abs. ml diff. (per slice) [ml]	1.33	5.48	2.50
**Midventricular Slices**			
Dice (all slices) [%]	94.21	85.87	91.77
Dice (slices contoured by both) [%]	94.21	87.60	91.88
HD [mm]	2.67	2.79	5.52
Abs. ml diff. (per slice) [ml]	0.57	1.21	0.91
**Apical Slices**			
Dice (all slices) [%]	69.59	62.93	66.07
Dice (slices contoured by both) [%]	80.36	68.84	74.35
HD [mm]	3.09	4.12	5.81
Abs. ml diff. (per slice) [ml]	0.38	0.89	0.59

Table. 1 Clinical Parameters and Metrics Table: The columnms are metric name, left ventricular endocardial contour, left ventricular myocardial contour, right ventricular endocardial contour. The table is divided into basal midventricular and apical slices. Basal slices are the most upper slice, apical the lowest slice (as defined by the first reader). Legend: LV left ventricular, RV: Right ventricular, Dice: Dice similarity coefficient, HD: Hausdorff distance, Abs. ml diff.: Absolute millilitre difference

Clinical Parameters and Metrics Table

Name	Mean	Std
LVEF [%]	3.04	4.00
LVEDV [ml]	2.10	7.01
LVESV [ml]	-3.08	6.59
Dice (all slices) [%]	92.09	3.63
Dice (slices contoured by both) [%]	93.26	3.21
HD [mm]	2.02	0.70
LVM [g]	-2.04	11.40
Dice (all slices) [%]	83.13	7.11
Dice (slices contoured by both) [%]	86.31	6.86
HD [mm]	2.17	0.78
RVEF [%]	-1.37	4.63
RVEDV [ml]	-3.83	9.88
RVESV [ml]	0.35	8.57
Dice (all slices) [%]	87.87	4.33
Dice (slices contoured by both) [%]	88.90	3.70
HD [mm]	4.51	1.42
Dice (all slices, all contours) [%]	88.61	3.28
Dice (slices contoured by both, all contours) [%]	90.09	2.92
HD (all contours) [mm]	3.04	0.83

Table. 2 Clinical Parameters and Metrics Table: The columns are Name (either clinical parameter or metric), Mean and Standard deviation (for difference if clinical parameter, or mean value for metric). Legend: LV: Left ventricle, RV: Right ventricle, EF: Ejection fraction, EDV: end-diastolic volume, ESV: end-systolic volume, Dice: Dice similarity coefficient, HD: Hausdorff distance

Qualitative Figures added during Manual Inspection

The following PDF pages reference figures, which were manually selected by the investigor and added to this report manually. Every figure has a title and comments that the investigator typed for elaboration.

Title: AI slightly overestimates LVESV LVESV_bland_altman

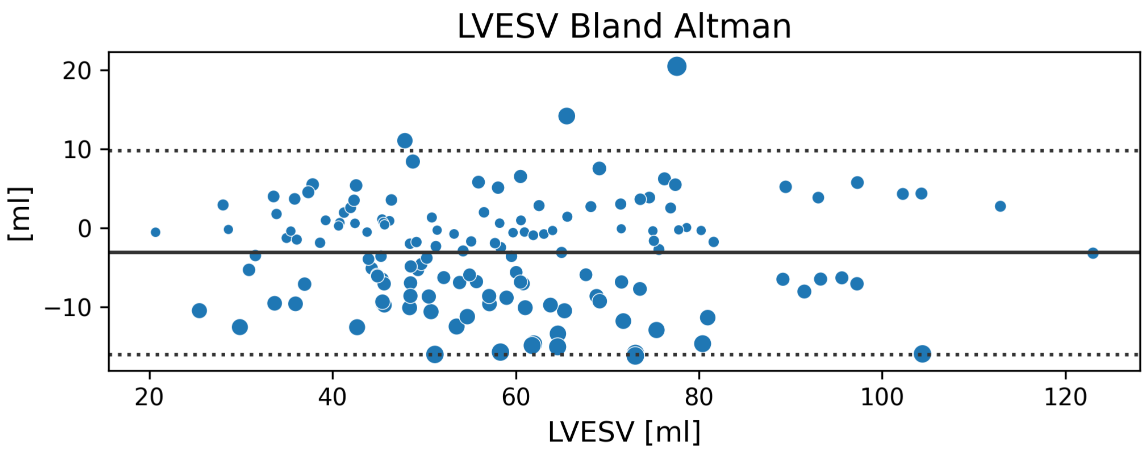

Perhaps an oversegmentation of LV endocardial contours. Investigating representative cases close to mean difference.

Title: Small systematic oversegmentation MyoMet-CMR-003_ category: SAX LVES, slice: 6 annotation comparison

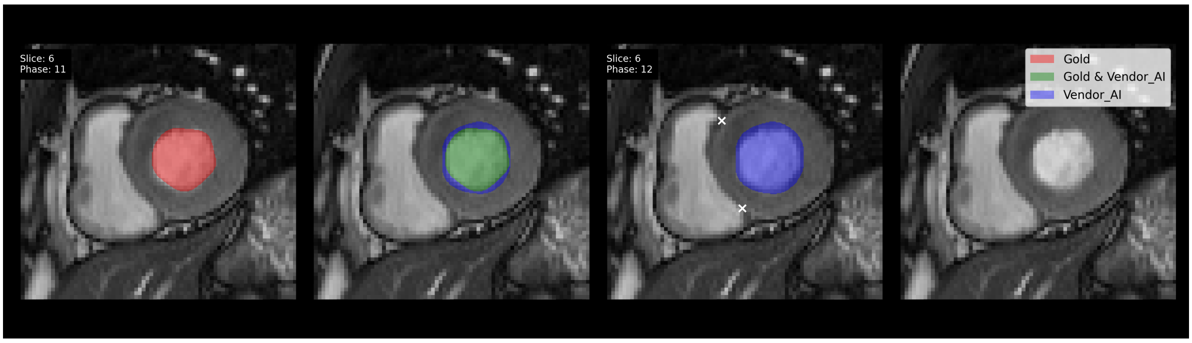

Approx. -1ml per slice. They add up.

Title: Small overestimation by AI RVEDV_bland_altman

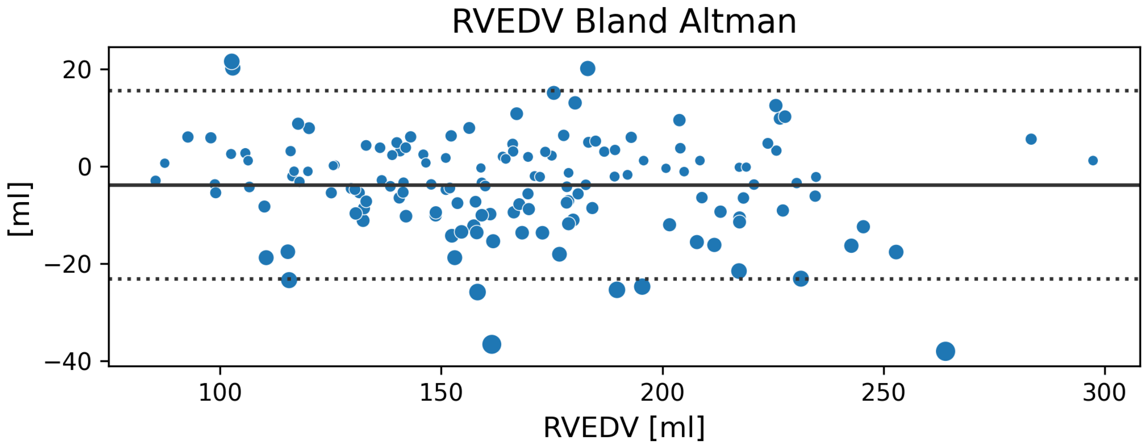

Investigating representative case for explanation. After that one outlier (approx. -40ml)

Title: Misinterpreted basal slice PSORCOR-051 category: SAX RVED, slice: 2 annotation comparison

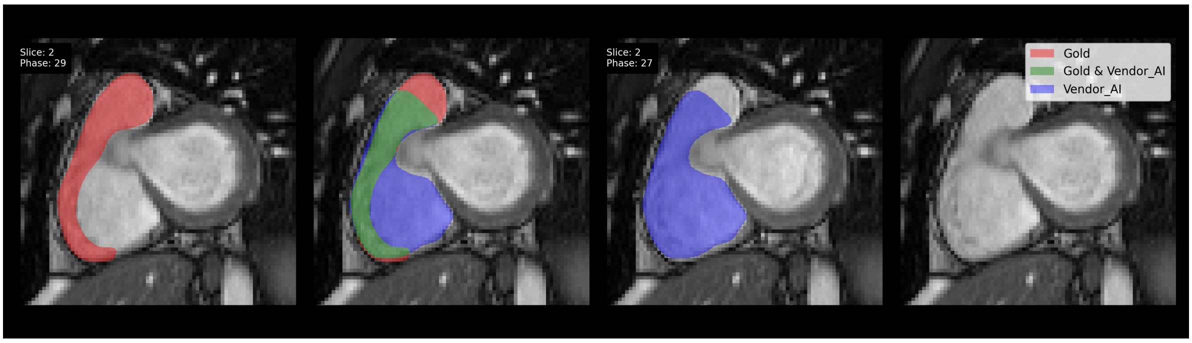

Approx. -9ml difference, which is compensated by midventricular undersegmentation. Next image...

Title: Slight undersegmentation of Midventricular slices PSORCOR-051 category: SAX RVED, slice: 6 annotation comparison

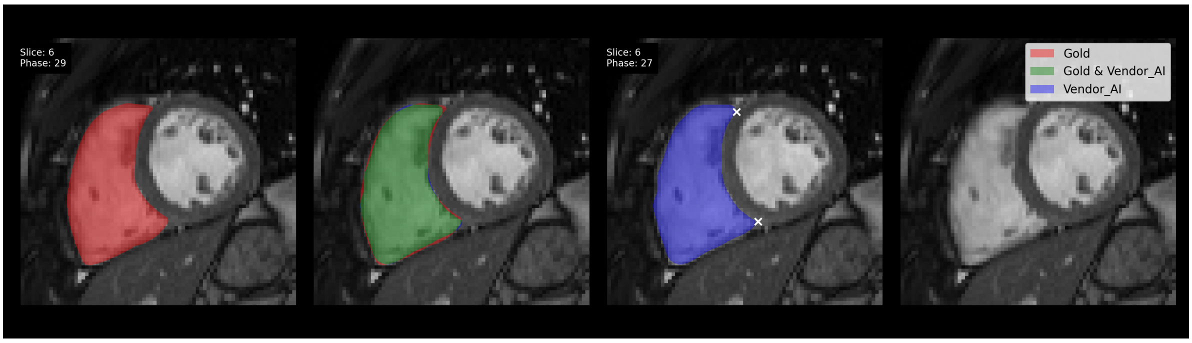

Add up to compensate basal difference, approx. 0.5ml per slice.

Title: RV outlier PSORCOR-049 category: SAX RVED, slice: 2 annotation comparison

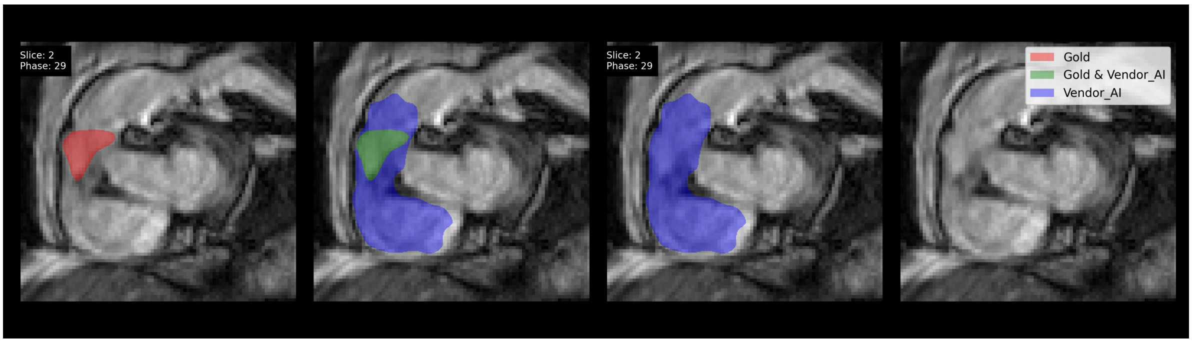

Difficult/blurry image, approx. -20ml.

Title: LVSV outside tolerance range LVSV_bland_altman

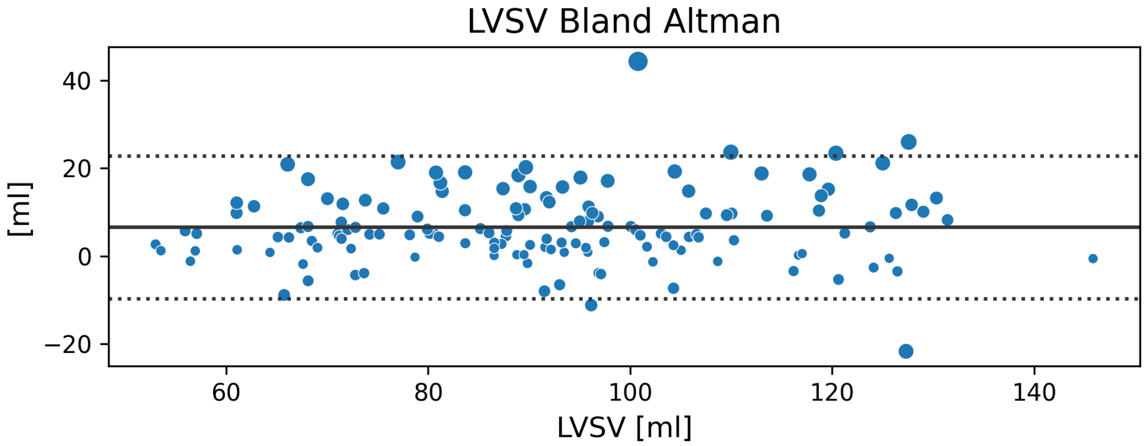

Investigating outliers (at 40ml and -20ml).

Title: Basal missegmented in ES PSORCOR-025 category: SAX LVES, slice: 3 annotation comparison

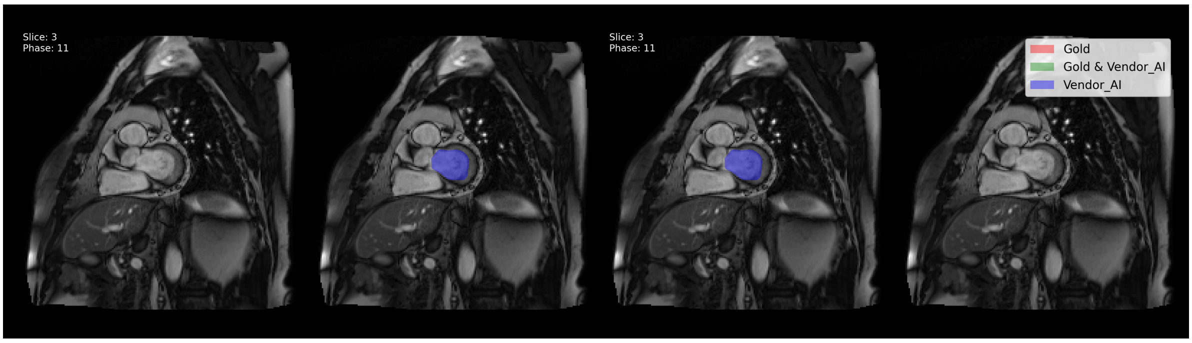

The large LVSV difference is caused by juxtaposed basal failures. In ES one slice too early and in ED ... (next image)

Title: Overlooked LVED PSORCOR-025 category: SAX LVED, slice: 2 annotation comparison

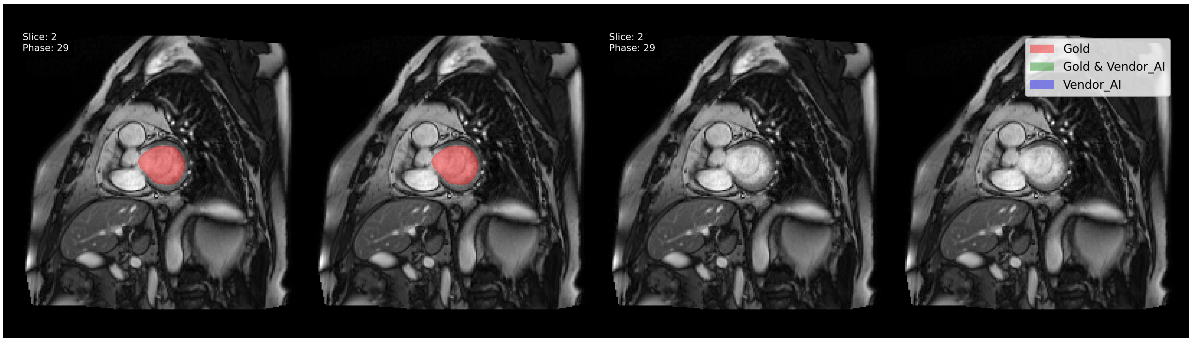

These juxtaposed slice identification failures cause the SV outlier.

Title: Other LVSV outlier MyoMet-CMR-014 category: SAX LVES, slice: 3 annotation comparison

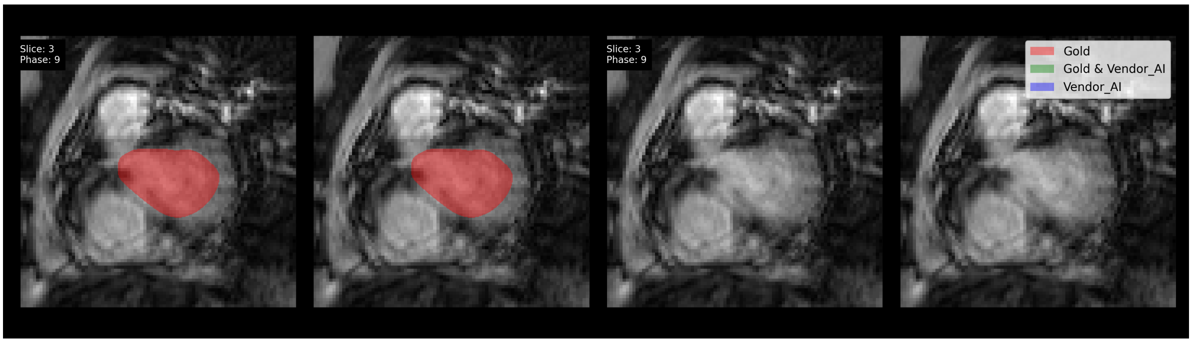

Overlooked in ES and ...

Title: LVSV outlier MyoMet-CMR-014 category: SAX LVED, slice: 1 annotation comparison

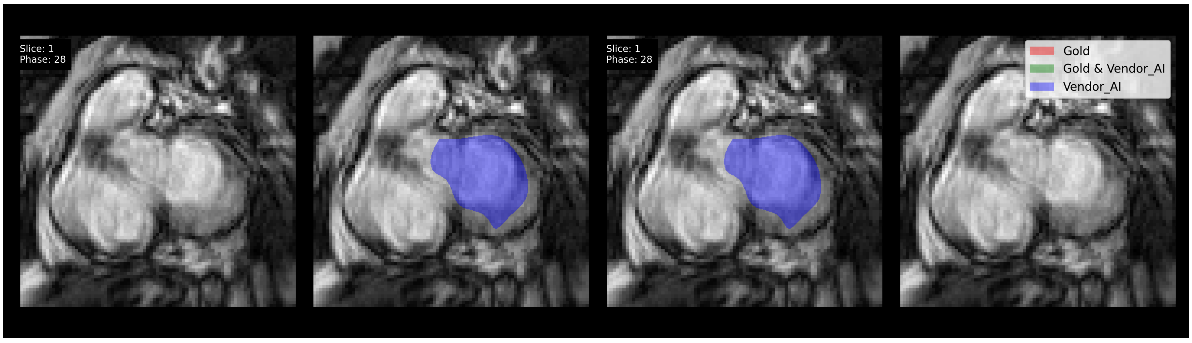

This time one slice too early in ED. Again juxtaposed failures add up to another outlier.

Title: LVSV difference PSORCOR-017 category: SAX LVES, slice: 7 annotation comparison

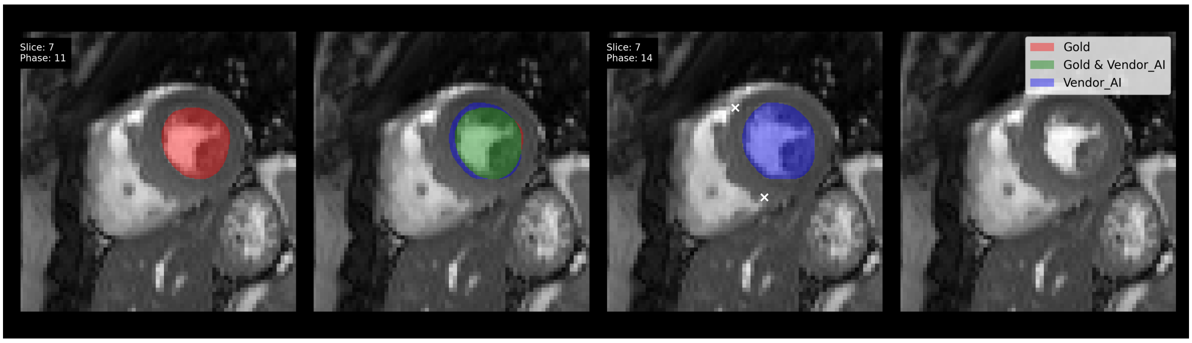

Oversegmentation of LV endocardium by AI in ES.

Title: LVSV difference PSORCOR-017 category: SAX LVED, slice: 9 annotation comparison

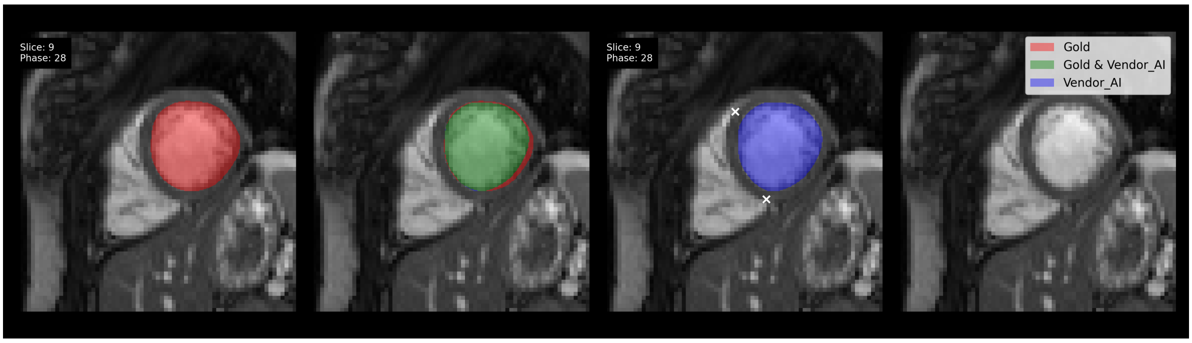

which is reinforced by a systematic undersegmentation in ED.

Title: Excellent LV Myomass LVM_bland_altman

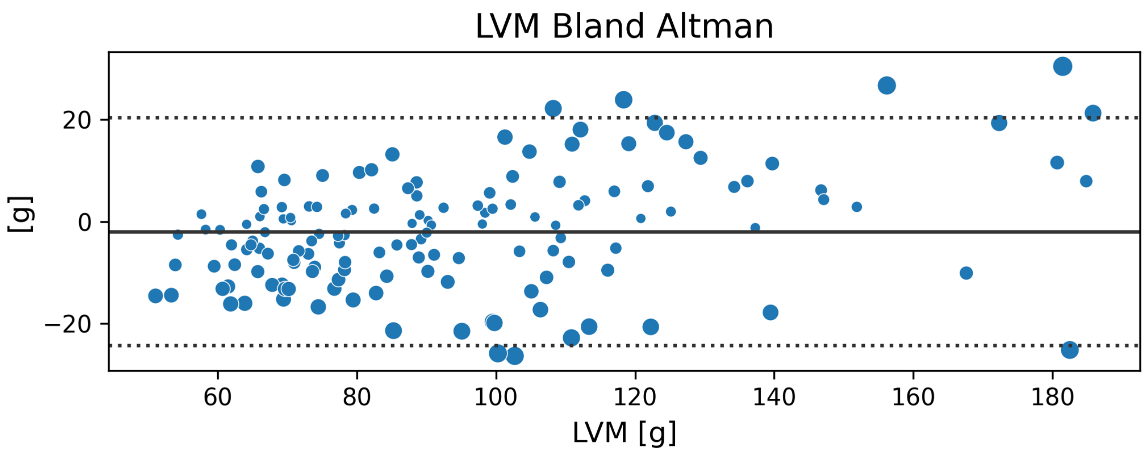

Investigating outliers.

Title: Overlooked basal slice MyoMet-CMR-065_034Y category: SAX LVED, slice: 2 annotation comparison

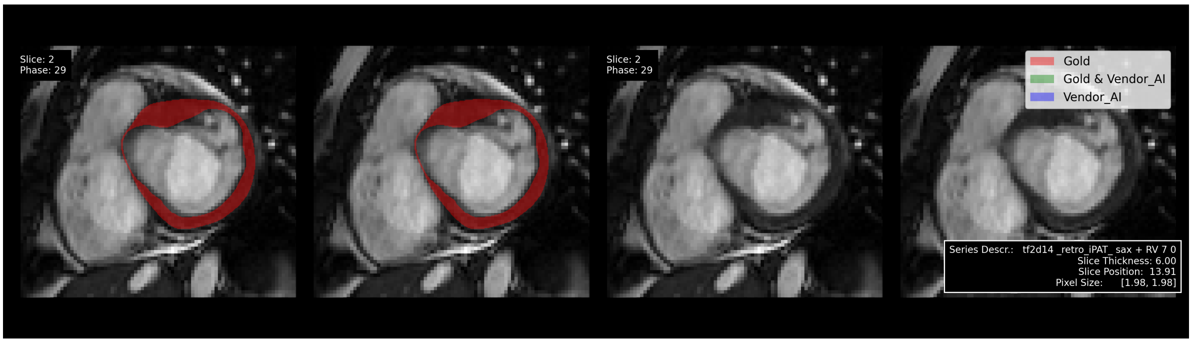

Possibly due to the thin mycardium?

Title: Slight undersegmentations MyoMet-CMR-065_034Y category: SAX LVED, slice: 9 annotation comparison

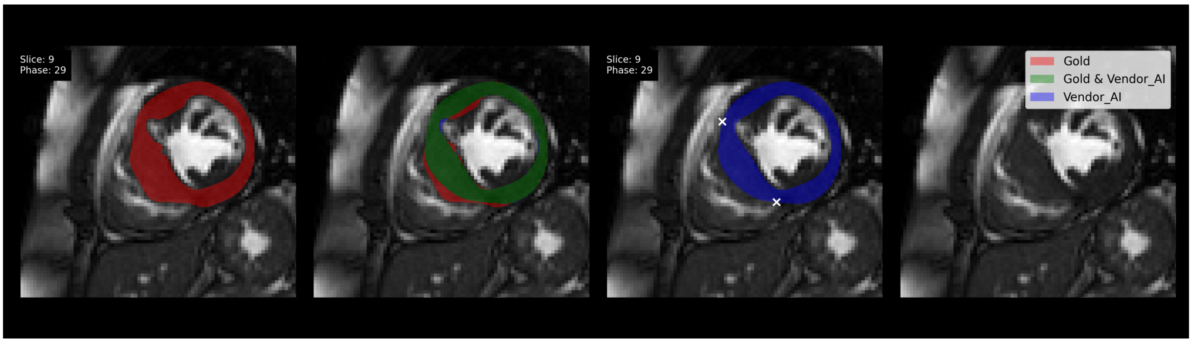

that add up. Difficult case as well.

Title: Two overlooked basal slices annotation comparison

MyoMet-CMR-047_032Y category: SAX LVED, slice: 3

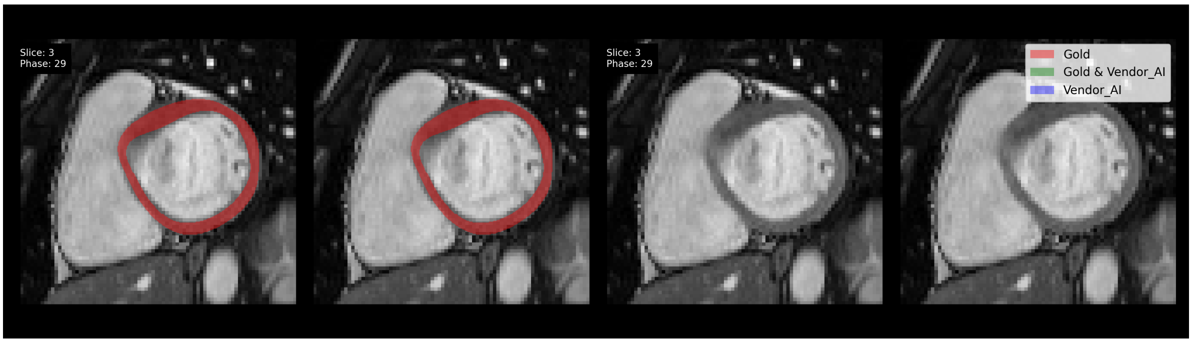

They add up to approx. 25ml.

Title: Overall good LVEDPAPMUM_bland_altman

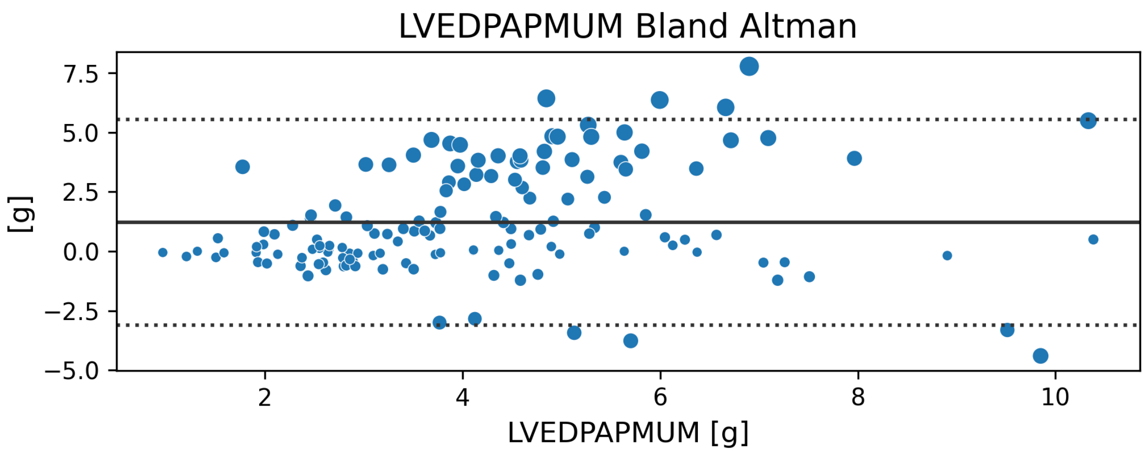

Overall good, larger deviations for more average amount of papillary muscles.

Title: Good estimations by AI PSORCOR-026 category: SAX LVED, slice: 8 annotation comparison

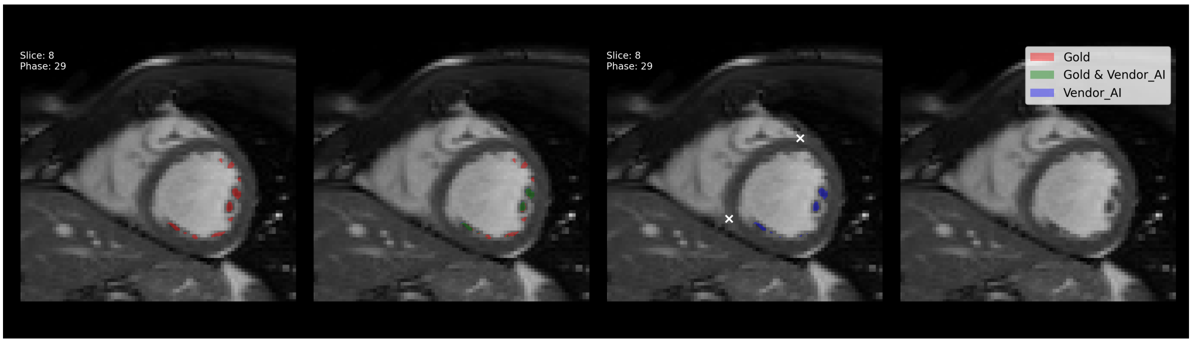

Expert opted for more small muscles.

Title: Overall good. PSORCOR-043 category: SAX LVES, slice: 7 annotation comparison

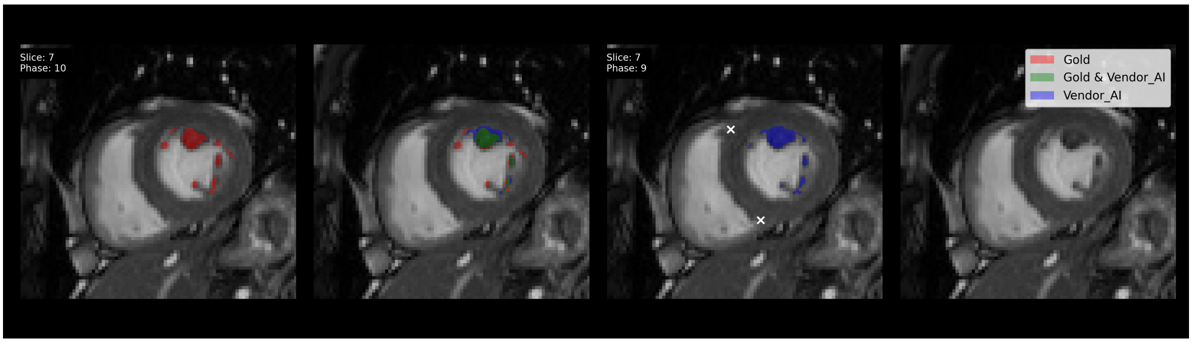

- Comments -
