# Supplementary material for: A semi-automated quality assurance tool for cardiovascular magnetic resonance imaging: application to outlier detection, artificial intelligence evaluation and trainee feedback
Source: BMC Med Inform Decis Mak. 2025 Dec 2;25:437. doi: 10.1186/s12911-025-03271-6 (PMC12670863; doi:10.1186/s12911-025-03271-6)
Supplement: Supplementary file 3 — Supplementary Material 3 [file 12911_2025_3271_MOESM3_ESM.pdf]

Clinical Parameter Means and Tolerance Ranges

Clinical Result (mean±std)	R1	R3	Difference	±Tolerance range
LVV [ml]	132.8 (41.6)	137.4 (46.2)	-4.6 (7.4)	(-9.7, 0.6), ±nan
LVMV [ml]	75.2 (29.7)	80.1 (28.4)	-4.9 (3.5)	(-7.3, -2.4), ±nan
LVM [g]	78.9 (31.2)	84.1 (29.8)	-5.1 (3.7)	(-7.7, -2.6), ±nan
SCARV [ml]	7.4 (4.5)	6.7 (4.0)	0.7 (1.7)	(-0.5, 1.8), ±nan
SCARM [g]	7.8 (4.8)	7.1 (4.2)	0.7 (1.8)	(-0.5, 1.9), ±nan
SCARF [%]	9.2 (3.7)	7.9 (3.1)	1.3 (1.7)	(0.1, 2.5), ±nan
EXCLVOL [ml]	0.0 (0.0)	0.0 (0.0)	0.0 (0.0)	(0.0, 0.0), ±nan
EXCLMASS [g]	0.0 (0.0)	0.0 (0.0)	0.0 (0.0)	(0.0, 0.0), ±nan
NOREFLOWVOL [ml]	0.0 (0.0)	0.0 (0.0)	0.0 (0.0)	(0.0, 0.0), ±nan
NOREFLOWF [%]	0.0 (0.0)	0.0 (0.0)	0.0 (0.0)	(0.0, 0.0), ±nan

Table. 1 This table shows the clinical parameter names in the first column. The other columns show statistics concerning the parameters. The first and second readers' means (stds) are shown in the second and third column, respectively. The mean and std of the differences between both readers is presented in the fourth column. The mean difference of both readers ± 95% confidence intervals are shown in parentheses with ±tolerance ranges thereafter. This provides information on whether the 95% estimate of the mean difference between both readers is within an acceptable limit.

Tolerance range paper: Zange L, Muehlberg F, Blaszczyk E, Schwenke S, Traber J, Funk S, et al. Quantification in cardiovascular magnetic resonance: agreement of software from three different vendors on assessment of left ventricular function, 2D flow and parametric mapping. J Cardiovasc Magn Reson. 2019 Dec;21(1):12.

Overview Assessment

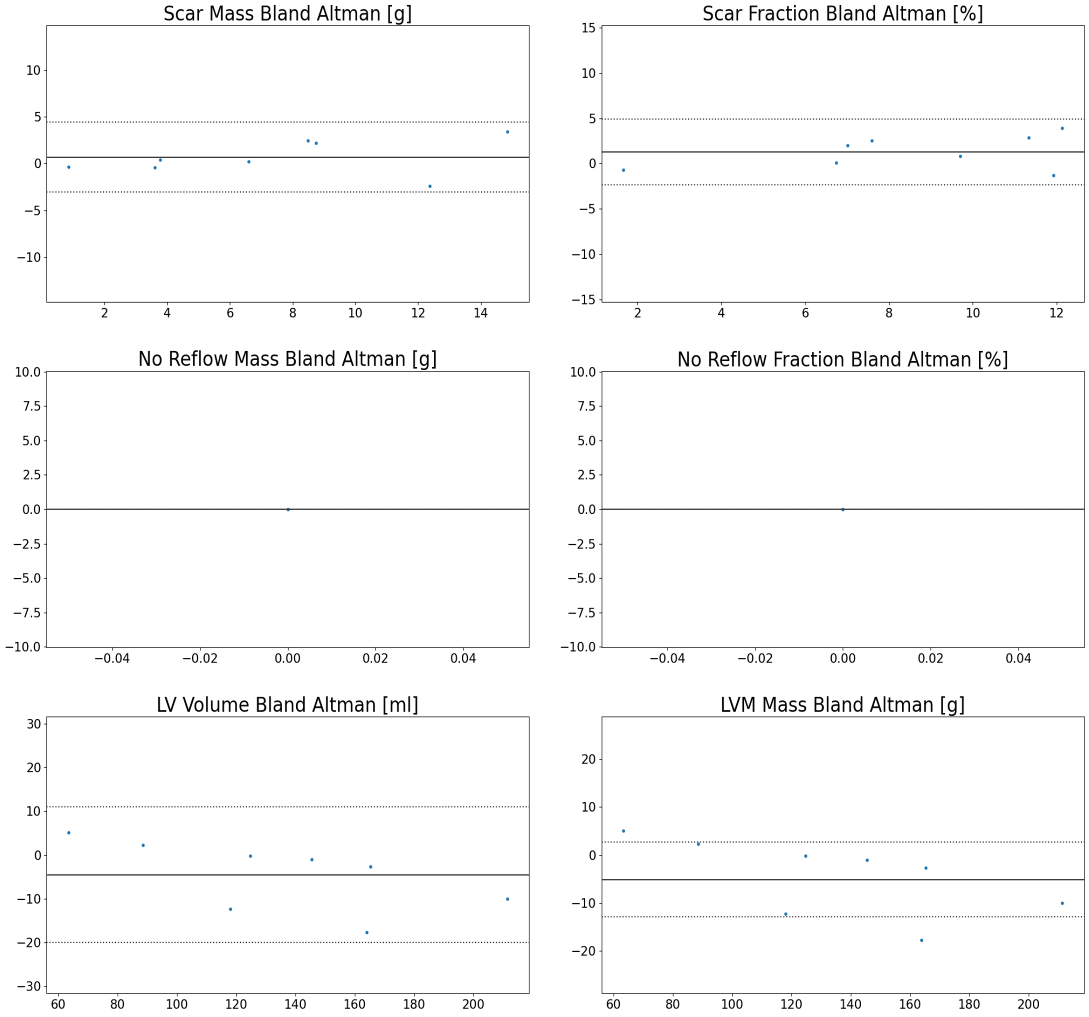

Fig. 1 Overview of Clinical Parameters: Upper left a Bland-Altman for the scar mass, upper right for the scar fraction defined as the volume of scar tissue divided by the volume of the left ventricular myocardium, middle left the mass of no reflow tissue, middle right the fraction of no reflow tissue defined as the volume of no reflow tissue divided by the volume of the left ventricular myocardium, the bottom left shows the left ventricular volume, and the left ventricle's myocardial mass on the botton right. Legend: Dice: Dice similarity coefficient, HD: Hausdorff distance

Overview Assessment

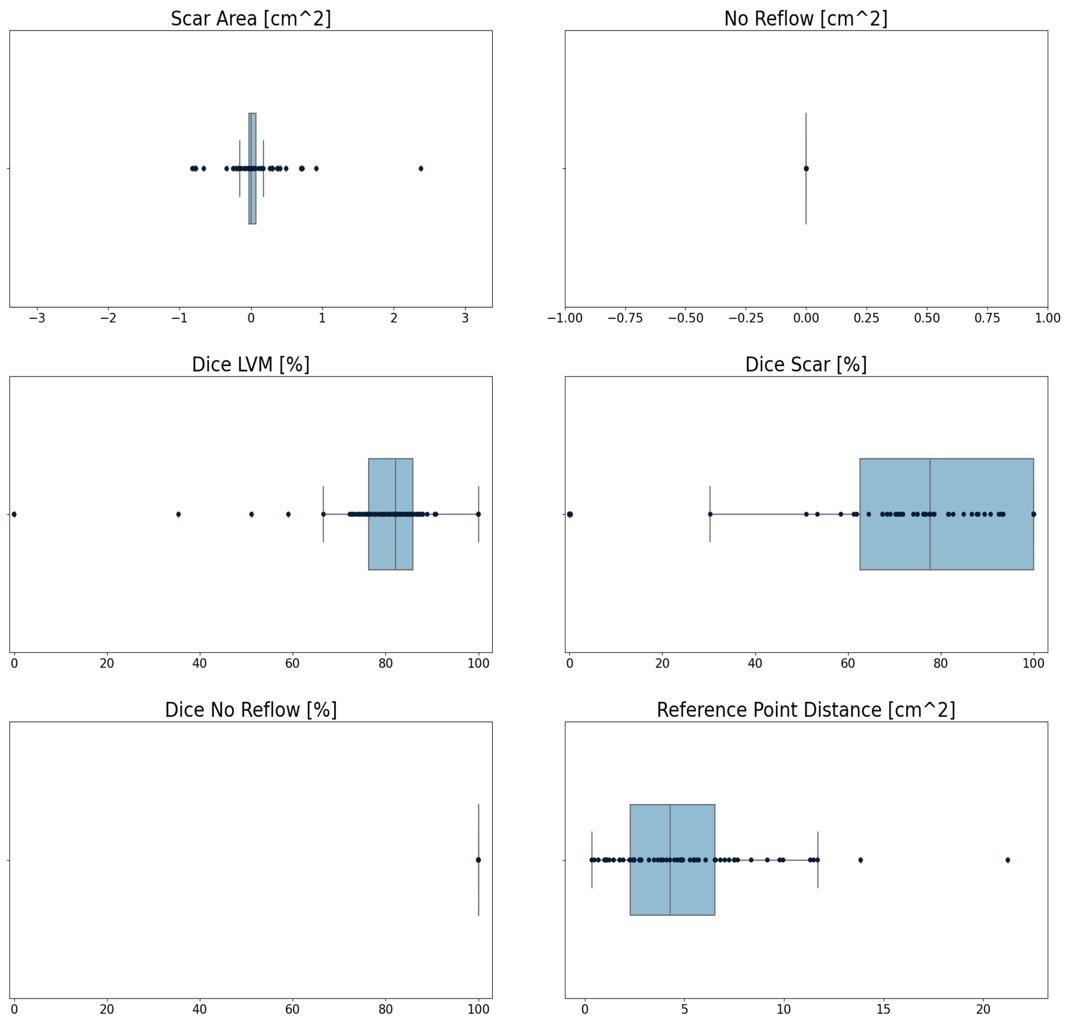

Fig. 2 Slice-based Overview of LGE values and Metrics as Scatter- ontop of Boxplots: Top left shows the scar area differences of all slices, top right the noreflow area differences, middle left the Dice of the LVM, middle right, the Dice of the scar contours, bottom left the Dice of no reflow contours, and the bottom right the distance of the reference points selected by the readers for individual slices. Legend: Dice: Dice similarity coefficient, LVM: Left ventricular myocardium

Qualitative Figures added during Manual Inspection

The following PDF pages reference figures, which were manually selected by the investigor and added to this report manually. Every figure has a title and comments that the investigator typed for elaboration.

Title: Slight underestimtation by second reader SCARM_bland_altman

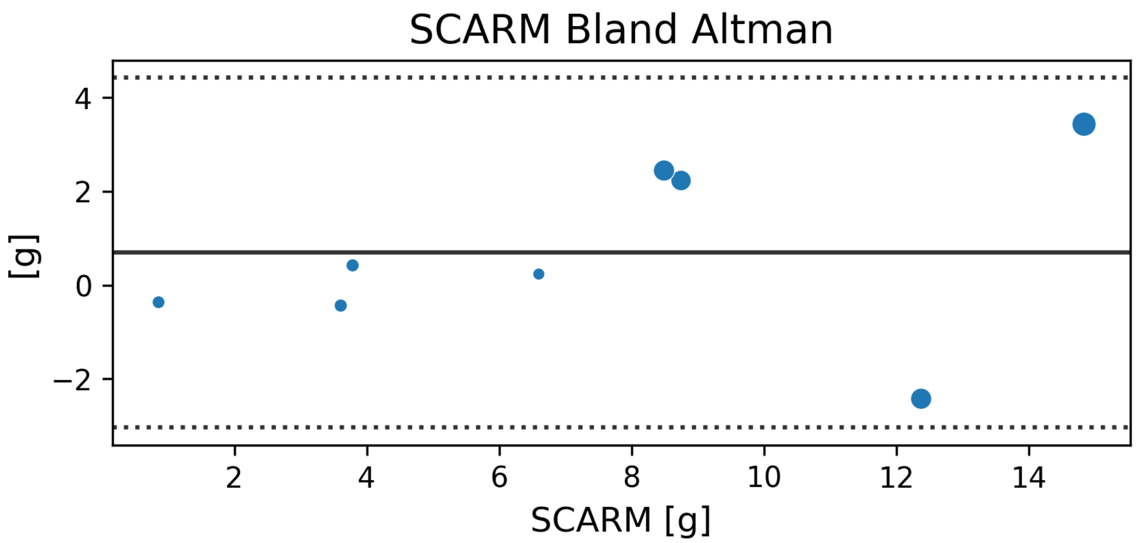

Investigating outliers.

Title: Myocardial differences 3DCS_LV_023_079Y(1) category: SAX LGE, slice: 4 annotation comparison

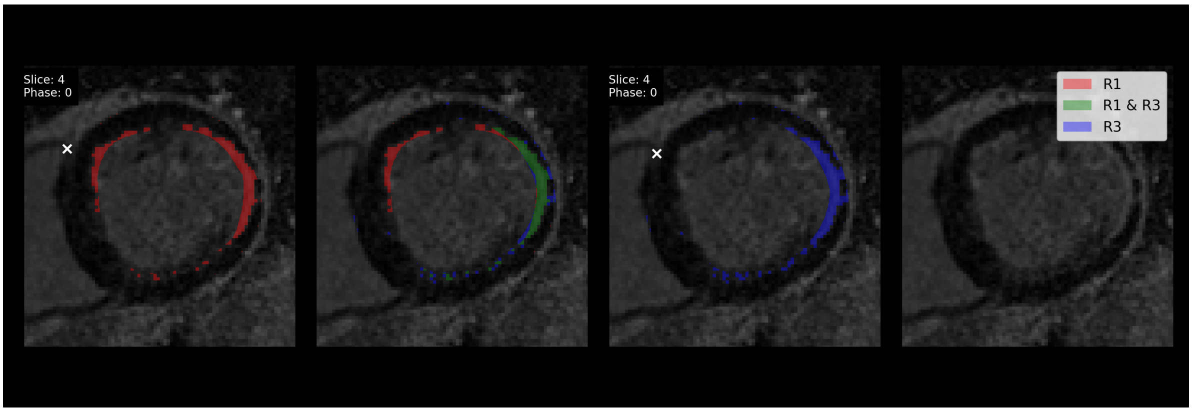

The second reader slightly oversegments the endocardial contour. This influences the assessed scar tissue. See next image.

Title: Interdependence Myocardium & Scar 3DCS_LV_023_079Y(1) category: SAX LGE, slice: 4 annotation comparison

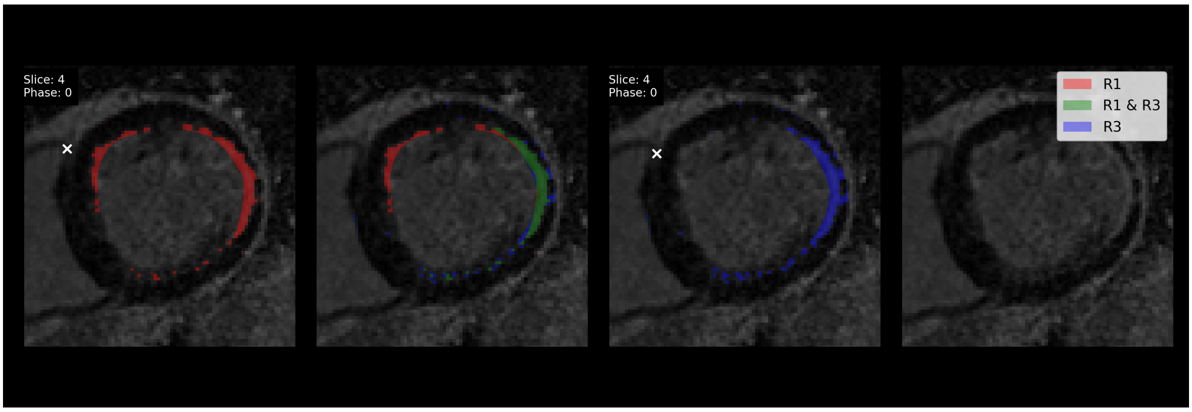

The first reader calculates more scar tissue due to more myocardium.

Title: Same phenomenon here. 3DCS_LV_023_079Y(1) category: SAX LGE, slice: 3 annotation comparison

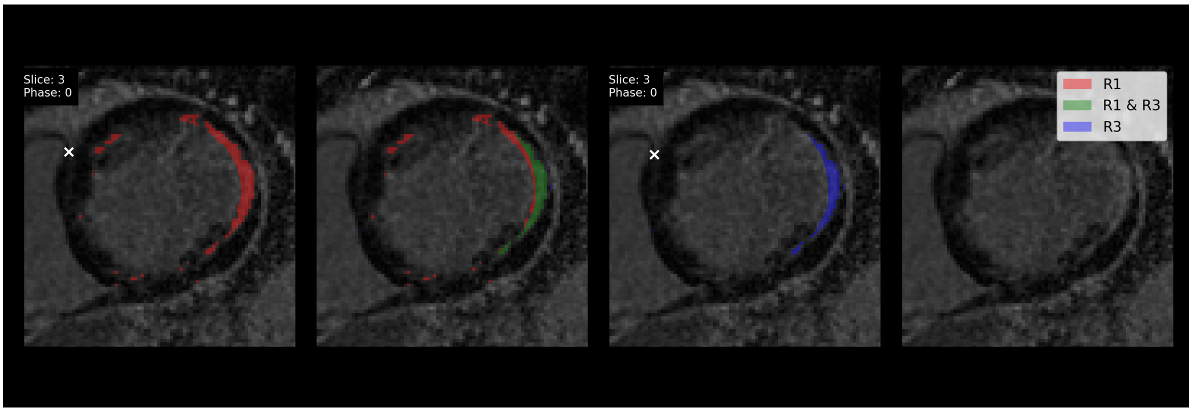

- Comments -
