# Supplementary material for: A semi-automated quality assurance tool for cardiovascular magnetic resonance imaging: application to outlier detection, artificial intelligence evaluation and trainee feedback
Source: BMC Med Inform Decis Mak. 2025 Dec 2;25:437. doi: 10.1186/s12911-025-03271-6 (PMC12670863; doi:10.1186/s12911-025-03271-6)
Supplement: Supplementary file 4 — Supplementary Material 4 [file 12911_2025_3271_MOESM4_ESM.pdf]

Clinical Parameter Means and Tolerance Ranges

Clinical Result (mean±std)	R1	R2	Diff(R1, R2)	(Mean Diff±CI), ±Tol range
GLOBAL_T1 [ms]	1138.1 (99.8)	1145.6 (101.3)	-7.5 (7.6)	(-11.8, -3.2), ±24.5

Table. 1 This table shows the clinical parameter names in the first column. The other columns show statistics concerning the parameters. The first and second readers' means (stds) are shown in the second and third column, respectively. The mean and std of the differences between both readers is presented in the fourth column. The mean difference of both readers ± 95% confidence intervals are shown in parentheses with ±tolerance ranges thereafter. This provides information on whether the 95% estimate of the mean difference between both readers is within an acceptable limit.

Overview Assessment

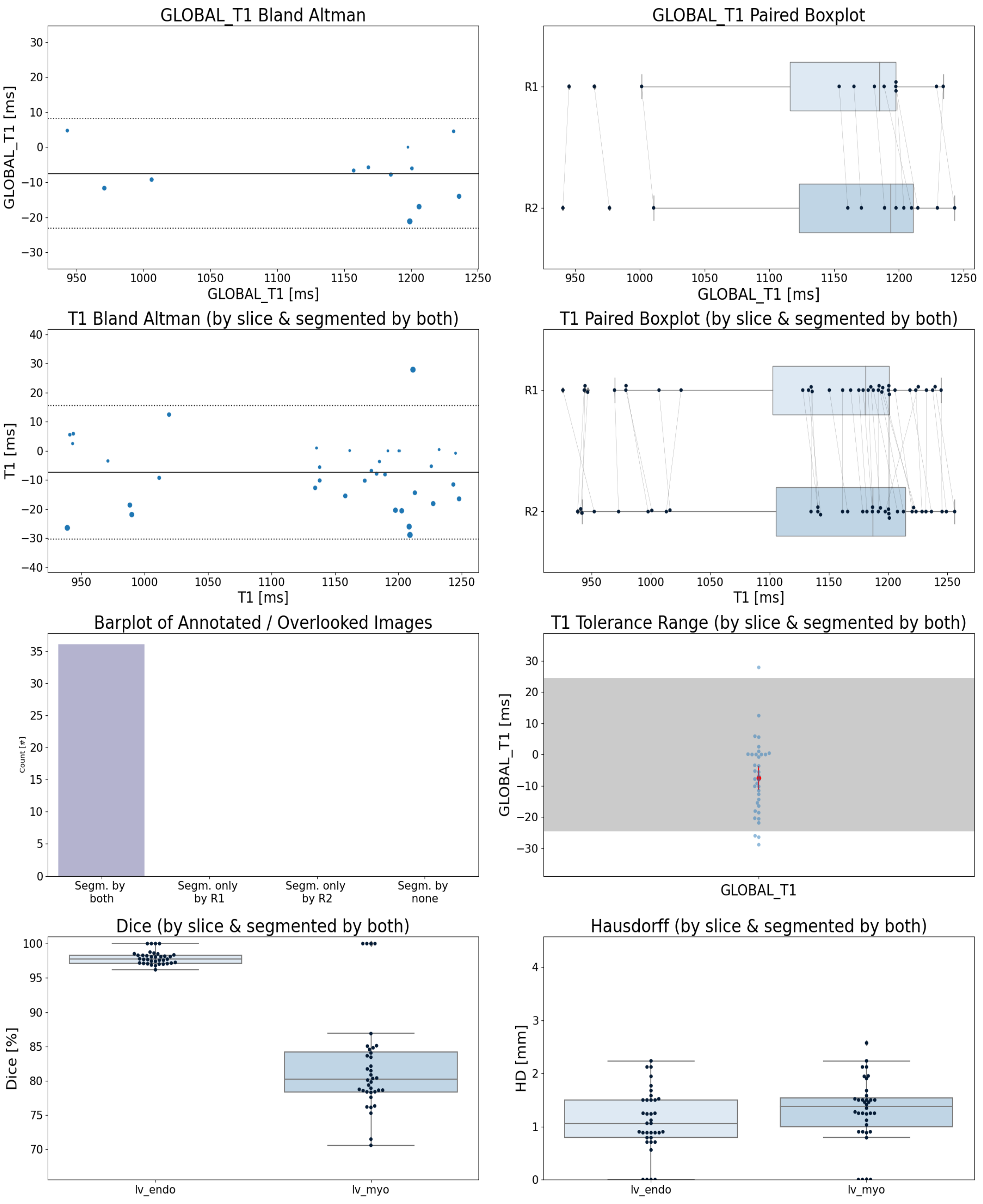

Fig. 1 Overview of Mapping values and Contour Metrics: Bland-Altman plots for Mapping values as points for all cases / all slices (first /second row). Point size shows magnitude of difference, the solid line marks mean difference between readers, the dashed lines mark mean differences ±1.96 standard deviations. Paired Boxplots show Mapping values as assessed by the first reader (on top) and the second reader below for all cases / all slices (first / second row). Lines connect same cases / slices to one another. Row four contains a histogram and a tolerance range plot. The histogram shows nr of slices contoured by both readers / only first reader / only second reader or not by either. The tolerance range is shown for all slices segmented by both readers (excluding "overlooked" slices). The gray bars represent ±tolerance range. The ±95% confidence interval is plotted as an errorbar in red around the average difference. The case value differences are plotted in blue. In the fourth row (left) dice values are plotted per contour type. On the right Hausdorff distance values are plotted per contour type. Legend: Dice: Dice similarity coefficient, HD: Hausdorff distance

Reference Point Differences

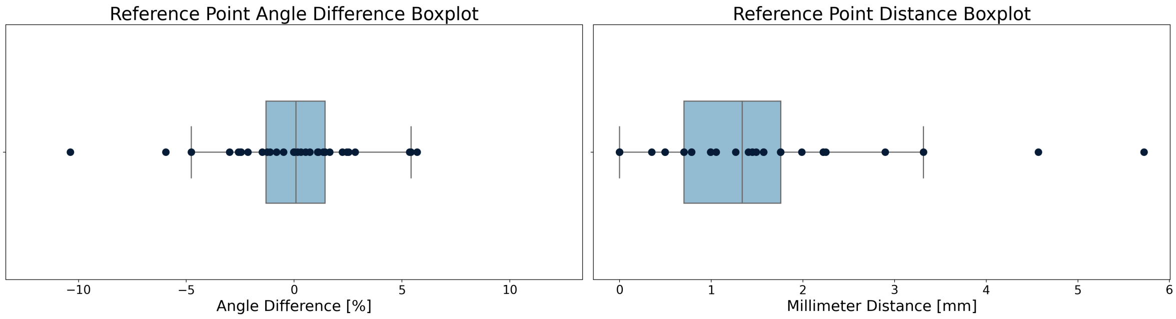

Fig. 2 Reference Point Difference Plots: On the left the angle differences between readers per slice are plotted as a scatter plot on top of a boxplot. The angles are defined as the angle between the line between the endocardial median point and the reference point. On the right the distances between reference points selected by both readers are plotted as a scatter plot on top of a boxplot. Legend: mm: Millimeter

Avg Differences AHA Model

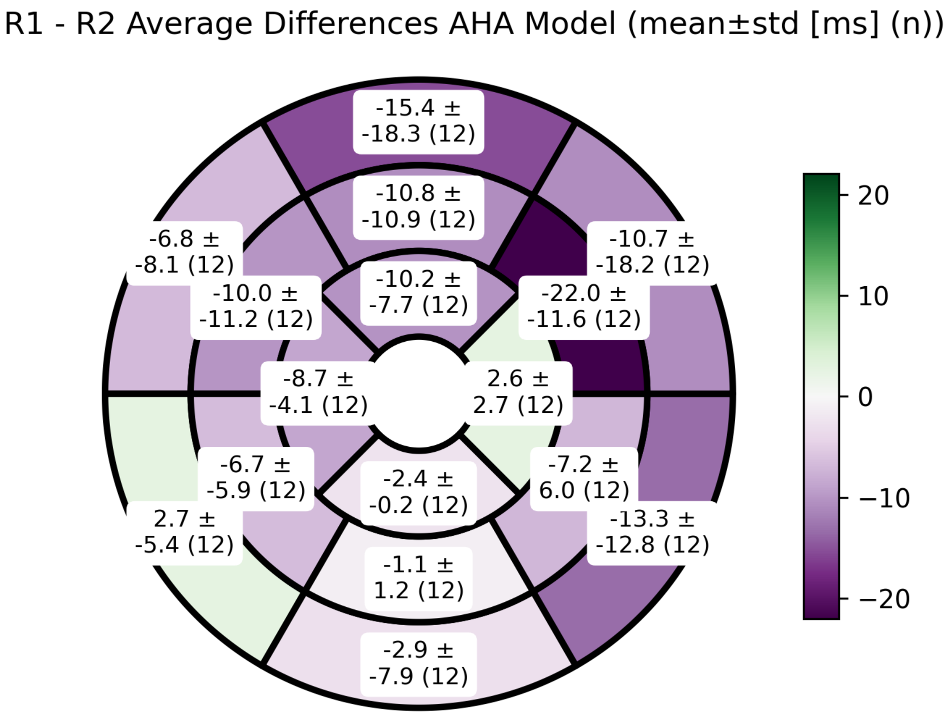

Fig. 5 Average Differences AHA Model: The AHA model is plotted for 16 segments reflecting the basal (6 outer segments), midventricular (6 middle segments) and apical (4 inner segments). Each segment contains a label with the mean±standard deviation (n). The mean and standard deviation pertain to the pixel value differences per segmet between the two readers. In parentheses the number of cases that provided values to this segment by both readers is shown. Legend: AHA: American Heart Association

Qualitative Figures added during Manual Inspection

The following PDF pages reference figures, which were manually selected by the investigor and added to this report manually. Every figure has a title and comments that the investigator typed for elaboration.

Title: Overall good T1 global values (in tolerance range) GLOBAL_T1_bland_altman

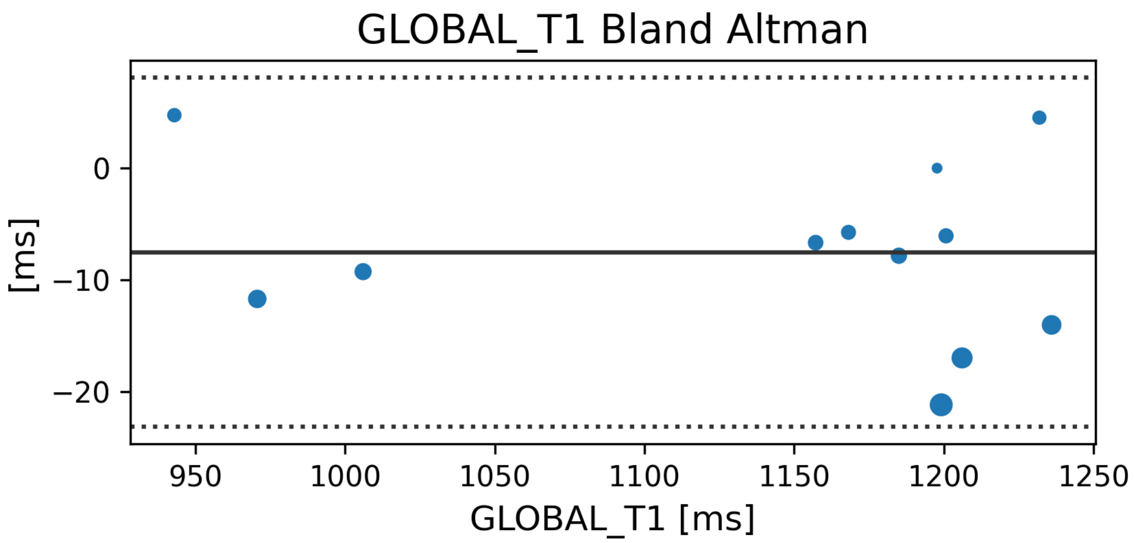

Global T1 is mean intensity of myocardial pixels). Single outliers to be investigated.

Title: Wider contours by R2 lead to large outlier Case: DZHK_TV_001_CCM_DZHK_TV_001_CCM_M, slice: 0

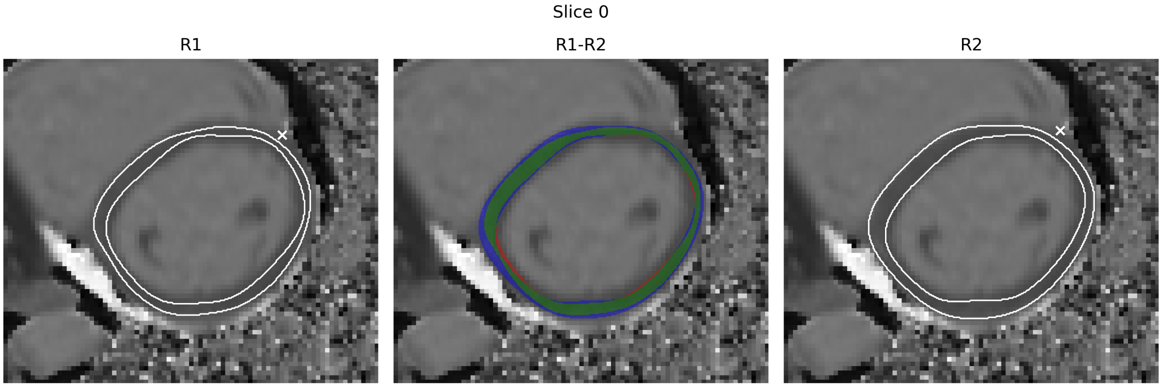

See the differences per segment in next image.

Title: Large differences Case: DZHK_TV_001_CCM_DZHK_TV_001_CCM_M, slice: 0

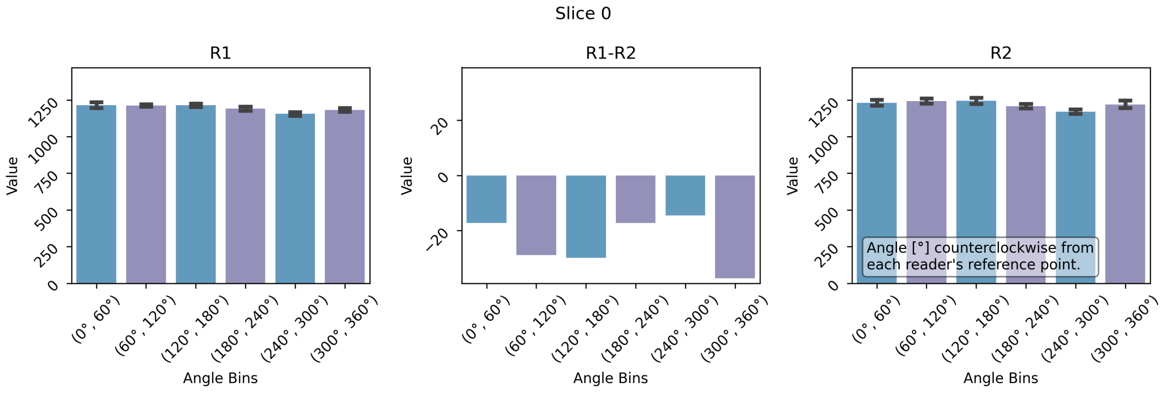

in each segment. (refers to above image)

Title: Myocardial disagreement... Case: DZHK_TV_005_Helios_DZHK_TV_005_Helios_F, slice: 1

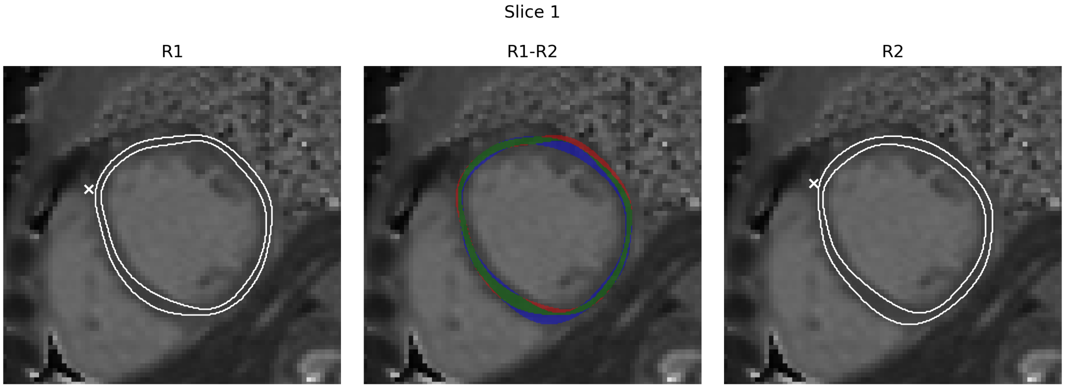

causes a large segmental outlier. Next image

Title: Large outlier (-89ms) Case: DZHK_TV_005_Helios_DZHK_TV_005_Helios_F, slice: 1

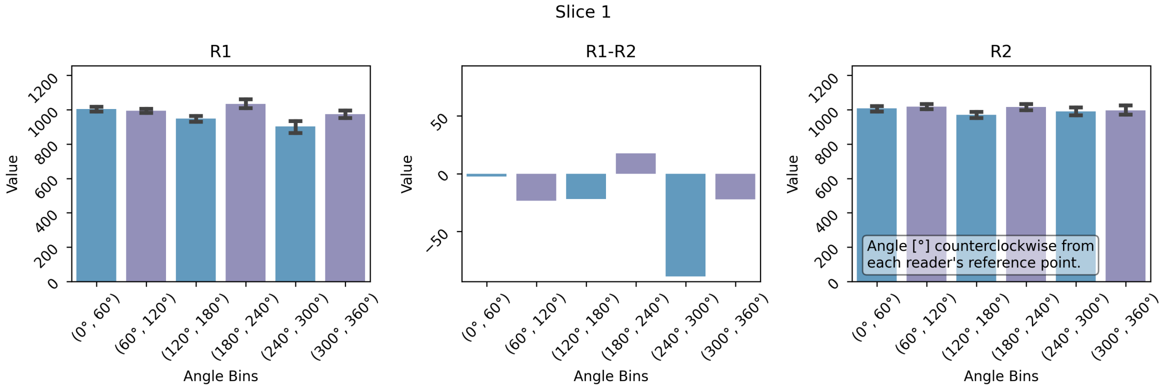

refers to above image.
