# Supplementary material for: A semi-automated quality assurance tool for cardiovascular magnetic resonance imaging: application to outlier detection, artificial intelligence evaluation and trainee feedback
Source: BMC Med Inform Decis Mak. 2025 Dec 2;25:437. doi: 10.1186/s12911-025-03271-6 (PMC12670863; doi:10.1186/s12911-025-03271-6)
Supplement: Supplementary file 6 — Supplementary Material 6 [file 12911_2025_3271_MOESM6_ESM.pdf]

Clinical Parameter Means and Tolerance Ranges

Clinical Result (mean±std)	Reader1_1	Reader1_2	Diff(Reader1_1, Reader1_2)	(Mean Diff±CI), ±Tol range
4CV_RAESAREA [cm^2]	14.0 (3.8)	14.0 (4.1)	0.0 (0.8)	(-0.2, 0.3), ±nan
4CV_RAEDAREA [cm^2]	22.6 (5.2)	22.4 (5.0)	0.1 (0.7)	(-0.1, 0.4), ±1.0
4CV_RAESV [ml]	41.2 (16.7)	40.9 (17.7)	0.3 (3.4)	(-1.0, 1.5), ±nan
4CV_RAEDV [ml]	76.7 (27.2)	75.7 (25.5)	1.0 (4.3)	(-0.5, 2.6), ±nan
4CV_LAESAREA [cm^2]	13.4 (4.4)	13.3 (4.3)	0.0 (0.9)	(-0.3, 0.4), ±nan
4CV_LAEDAREA [cm^2]	23.5 (4.6)	23.6 (4.8)	-0.1 (0.9)	(-0.5, 0.2), ±2.1
4CV_LAESV [ml]	31.8 (18.2)	31.4 (17.7)	0.4 (3.5)	(-0.9, 1.6), ±nan
4CV_LAEDV [ml]	78.0 (26.8)	78.8 (28.1)	-0.8 (5.4)	(-2.8, 1.2), ±nan
2CV_LAESAREA [cm^2]	12.2 (5.5)	11.9 (5.6)	0.3 (0.7)	(0.0, 0.5), ±nan
2CV_LAEDAREA [cm^2]	20.7 (5.5)	20.8 (5.6)	-0.0 (0.7)	(-0.3, 0.2), ±2.0
2CV_LAESV [ml]	32.1 (24.4)	31.4 (25.2)	0.7 (3.1)	(-0.4, 1.8), ±nan
2CV_LAEDV [ml]	67.8 (28.3)	67.9 (29.3)	-0.1 (4.1)	(-1.6, 1.4), ±nan
BIPLANE_LAESV [ml]	34.6 (19.9)	34.1 (19.7)	0.5 (2.7)	(-0.5, 1.5), ±nan
BIPLANE_LAEDV [ml]	76.1 (24.0)	76.5 (25.4)	-0.4 (3.1)	(-1.5, 0.7), ±nan
LAESP_4CV [#]	4.3 (9.9)	2.3 (7.3)	0.2 (0.4)	(0.1, 0.4), ±nan
LAEDP_4CV [#]	13.5 (1.6)	13.6 (1.7)	0.2 (0.4)	(0.1, 0.4), ±nan
LAESP_2CV [#]	4.3 (9.9)	2.3 (7.3)	0.2 (0.4)	(0.1, 0.4), ±nan
LAEDP_2CV [#]	13.5 (1.6)	13.6 (1.7)	0.2 (0.4)	(0.1, 0.4), ±nan
RAESP_4CV [#]	7.3 (12.3)	5.2 (10.8)	0.4 (0.6)	(0.2, 0.7), ±nan
RAEDP_4CV [#]	13.2 (1.8)	13.2 (1.7)	0.3 (0.5)	(0.1, 0.5), ±nan

Table. 1 This table shows the clinical parameter names in the first column. The other columns show statistics concerning the parameters. The first and second readers' means (stds) are shown in the second and third column, respectively. The mean and std of the differences between both readers is presented in the fourth column. The mean difference of both readers ± 95% confidence intervals are shown in parentheses with ±tolerance ranges thereafter. This provides information on whether the 95% estimate of the mean difference between both readers is within an acceptable limit.

Atrial Area Differences

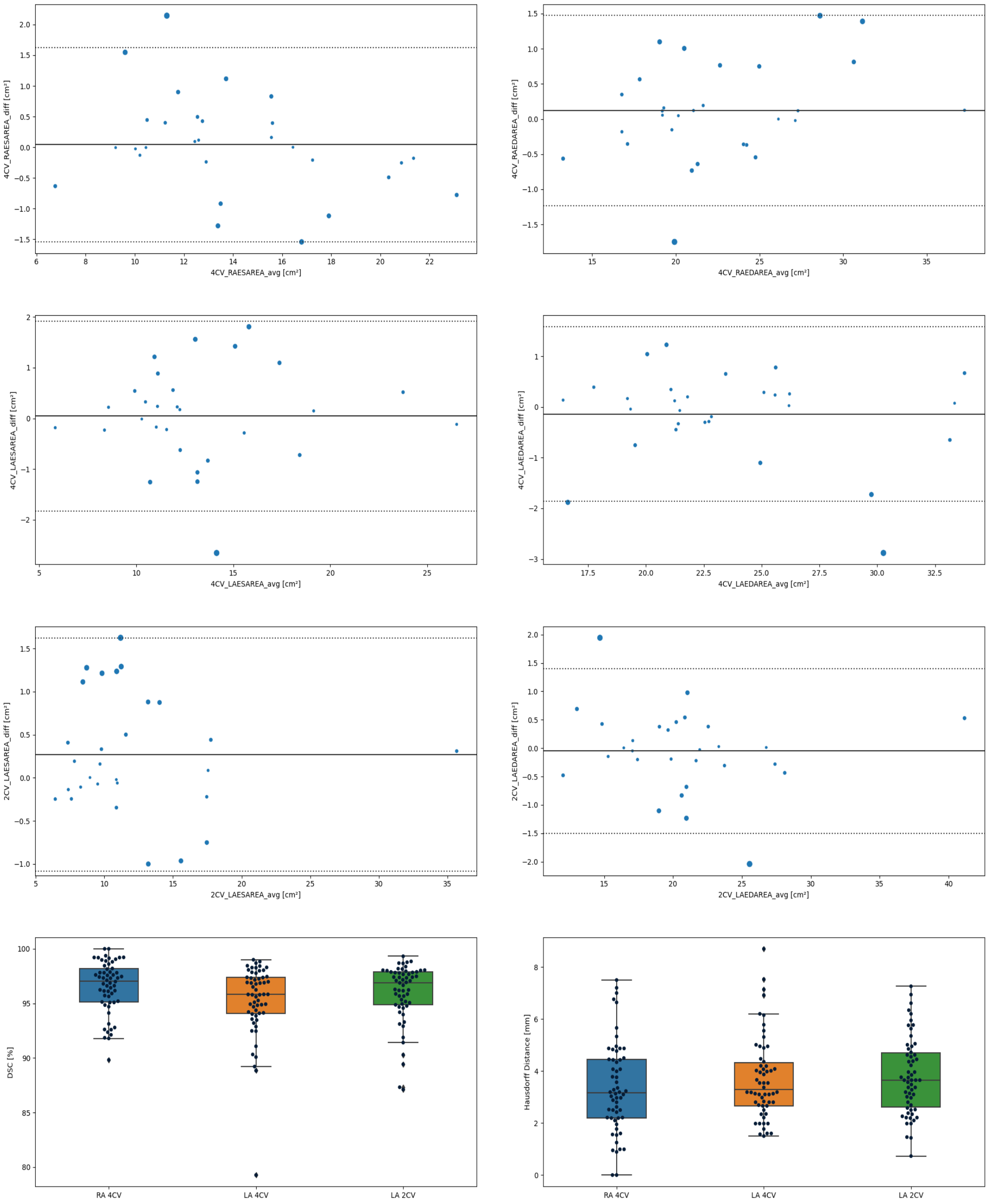

Fig. 1 Area Differences for LA & RA: The first row presents Bland-Altman plots for the 4CV RA areas in ES and ED. The second row shows BA plots for the 4CV LA areas. The third row shows 2CV LA areas. The last row contains Dice value boxplots per contour on the left and Hausdorff distance boxplots on the right. Legend: ES: End-systole, ED: End-diastole, CV: Chamber View, LA: Left Atrium, RA: Right Atrium, Dice: Dice similarity coefficient

Confidence Intervals and Tolerance Ranges

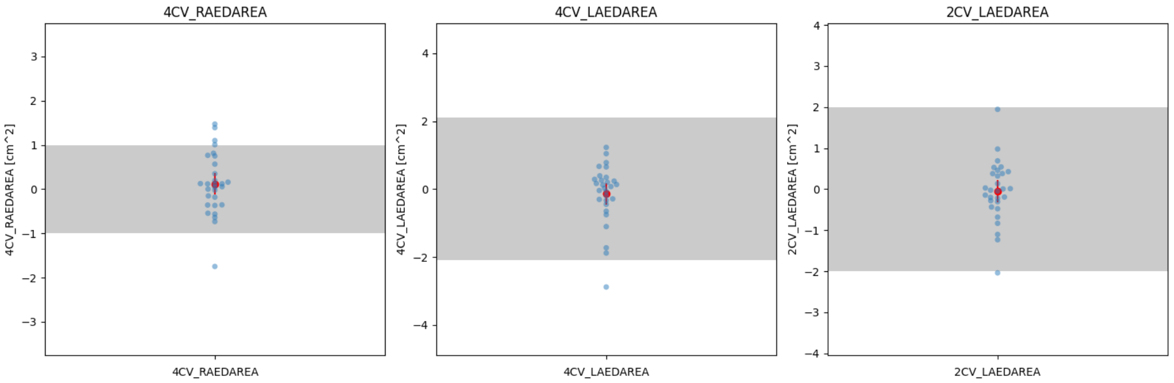

Fig. 2 Confidence Intervals and Tolerance Ranges of Atria Areas: Each subfigure references an atrial area, from left to right, 4CV RA, 4CV LA, 2CV LA. Tolerance intervals are shown as gray bars and represent ±1.96 standard deviation of an expert intrareader deviation. The 95% confidence intervals of the mean area difference is represented as an error bar in red. Individual area differences per contour are plotted in blue. Legend: CV: Chamber view, RA: Right Atrium, LA: Left Atrium

Qualitative Figures added during Manual Inspection

The following PDF pages reference figures, which were manually selected by the investigor and added to this report manually. Every figure has a title and comments that the investigator typed for elaboration.

Title: Unreasonably Large Outlier... 4CV_RAEDAREA_bland_altman

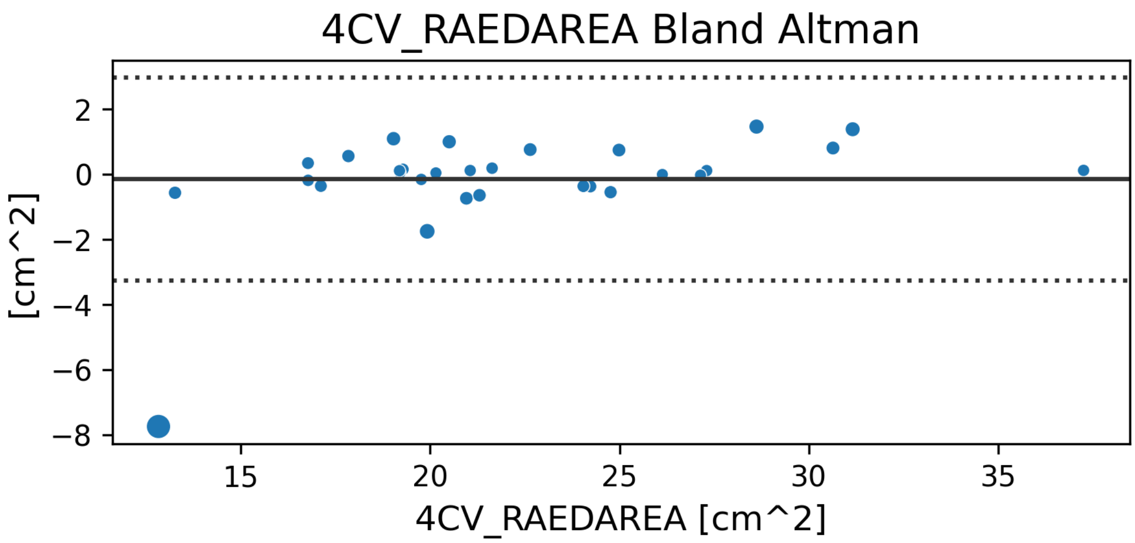

Checking for overlooked contours in RA ED...

Title: Paired Boxplot WITHOUT the outlier 4CV_RAEDAREA_paired_boxplot

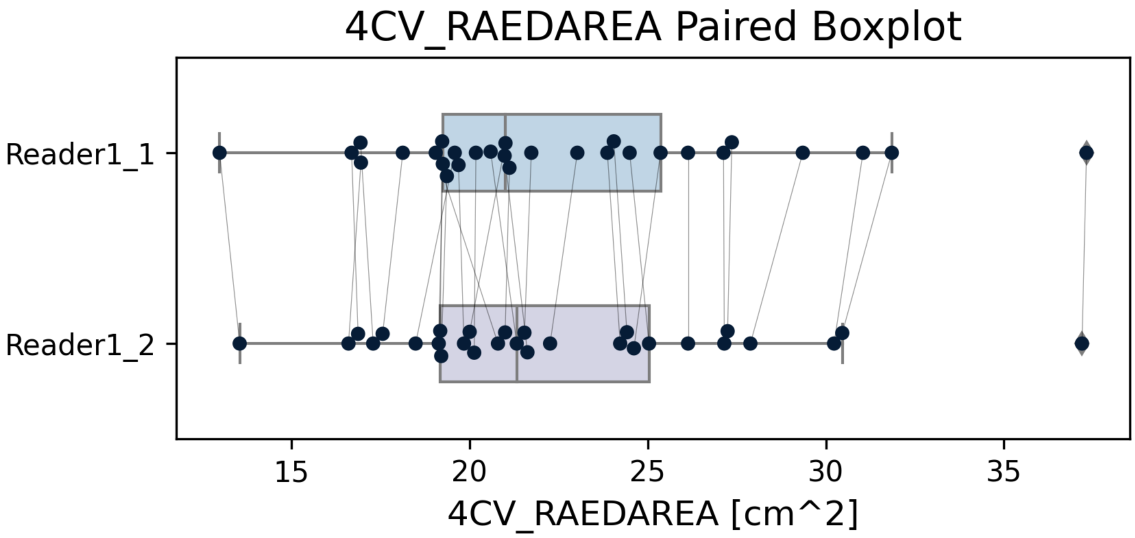

Reason for exclusion was indeed overlooked atria in RA ED by Reader_1_2

Title: Otherwise, well within tolerance range. 4CV_LAEDAREA_bland_altman

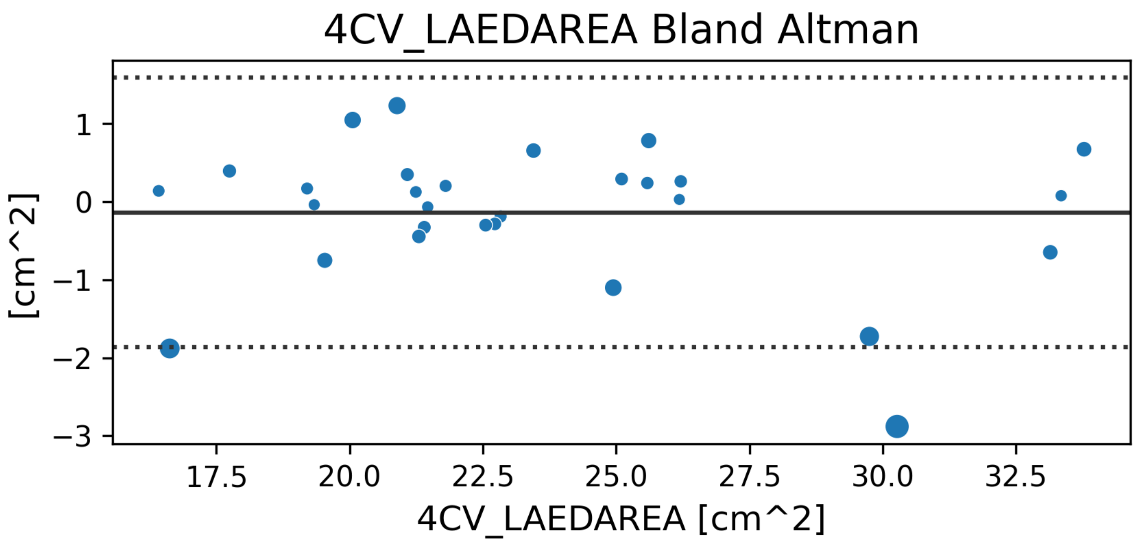

Inspecting largest outlier

Title: Large atrium Atria_HCM_24_ category: LAX 4CV LAED, slice: 0 annotation comparison

Category: LAX 4CV LAED, slice: 0

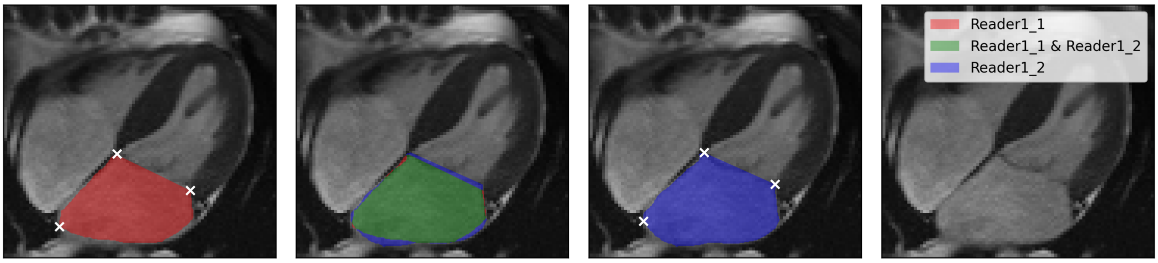

Otherwise very similar contours (DSC>93%)

Title: LA 2CV also excellent 2CV_LAEDAREA_bland_altman

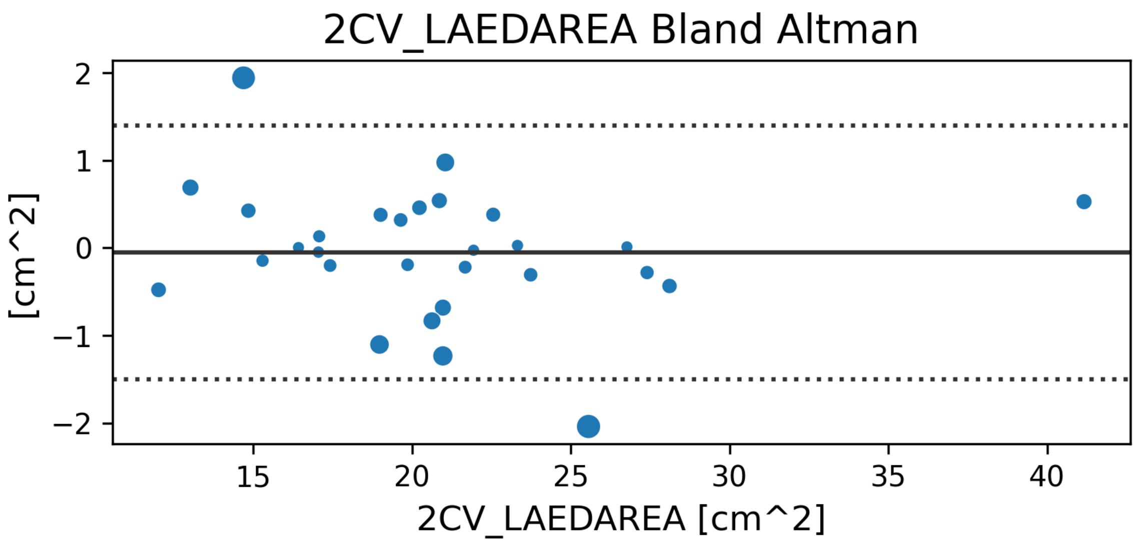

Checking two largest outliers... (top & bottom)

Title: Larger difference, but Atria_VOL_18_ category: LAX 2CV LAED, slice: 0 annotation comparison

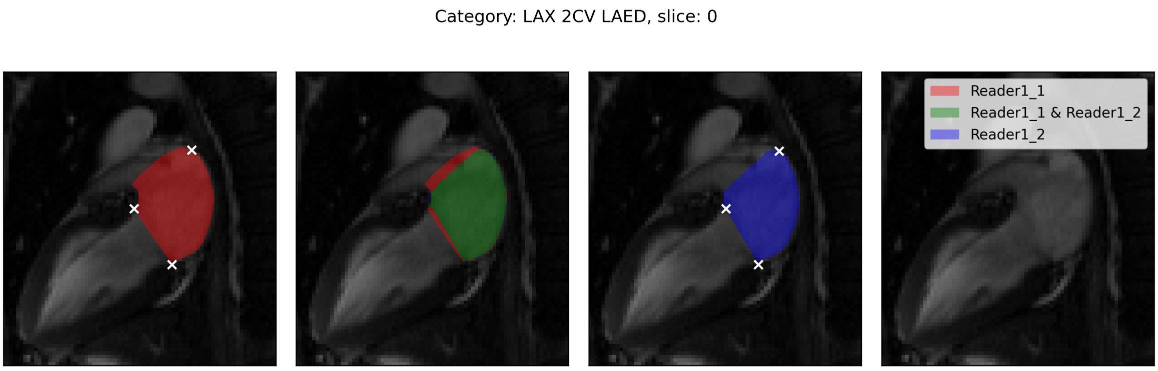

still highly similar contours (DSC approx 92%)

Title: Large atrium Atria_MD_9_ category: LAX 2CV LAED, slice: 0 annotation comparison

Category: LAX 2CV LAED, slice: 0

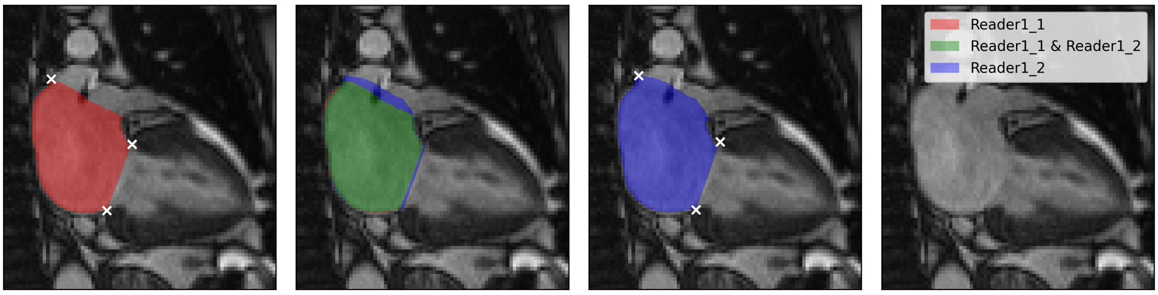

Otherwise very similar contours...
