# Supplementary material for: A semi-automated quality assurance tool for cardiovascular magnetic resonance imaging: application to outlier detection, artificial intelligence evaluation and trainee feedback
Source: BMC Med Inform Decis Mak. 2025 Dec 2;25:437. doi: 10.1186/s12911-025-03271-6 (PMC12670863; doi:10.1186/s12911-025-03271-6)

Clinical Parameter Means and Tolerance Ranges

Clinical Result (mean±std)	NI	R.H.13.02.2023	Difference	±Tolerance range
LVESV [ml]	65.7 (52.0)	61.4 (47.8)	4.3 (6.7)	(0.2, 8.4), ±7.3
LVEDV [ml]	146.1 (54.7)	155.3 (58.3)	-9.3 (6.2)	(-13.1, -5.4), ±10.8
RVESV [ml]	73.8 (23.5)	61.0 (21.5)	12.8 (7.5)	(8.1, 17.4), ±9.7
RVEDV [ml]	147.3 (42.6)	150.8 (48.8)	-3.4 (11.6)	(-10.7, 3.8), ±14.6
LVSV [ml]	76.6 (20.3)	91.6 (20.4)	-15.0 (7.7)	(-19.8, -10.2), ±4.5
LVEF [%]	54.2 (12.9)	60.3 (12.3)	-6.1 (4.1)	(-8.7, -3.6), ±5.3
RVSV [ml]	73.6 (20.9)	89.8 (32.8)	-16.2 (13.7)	(-24.7, -7.7), ±13.2
RVEF [%]	50.2 (4.8)	59.4 (8.7)	-9.2 (5.1)	(-12.4, -6.1), ±5.5
LVM [g]	107.1 (29.3)	87.1 (27.8)	20.0 (13.2)	(11.9, 28.2), ±13.3
RVM [g]	0.0 (0.0)	0.0 (0.0)	0.0 (0.0)	(0.0, 0.0), ±nan
LVESPAPMUM [g]	10.2 (5.1)	9.9 (8.7)	0.3 (4.8)	(-2.7, 3.3), ±nan
LVEDPAPMUM [g]	6.3 (2.2)	7.5 (4.4)	-1.2 (2.7)	(-2.9, 0.4), ±nan
LVESP [#]	10.7 (1.3)	11.2 (1.3)	0.9 (0.5)	(0.6, 1.2), ±nan
RVESP [#]	11.8 (2.0)	11.2 (1.3)	1.0 (1.5)	(0.1, 1.9), ±nan
LVEDP [#]	26.0 (8.7)	23.2 (11.6)	0.2 (0.4)	(-0.0, 0.4), ±nan
RVEDP [#]	24.6 (8.0)	23.2 (11.6)	2.0 (1.3)	(1.2, 2.8), ±nan
NrSlices [#]	16.5 (1.1)	16.5 (1.1)	0.0 (0.0)	(0.0, 0.0), ±nan

Table. 1 This table shows the clinical parameter names in the first column. The other columns show statistics concerning the parameters. The first and second readers' means (stds) are shown in the second and third column, respectively. The mean and std of the differences between both readers is presented in the fourth column. The mean difference of both readers ± 95% confidence intervals are shown in parentheses with ±tolerance ranges thereafter. This provides information on whether the 95% estimate of the mean difference between both readers is within an acceptable limit.

Tolerance range paper: Zange L, Muehlberg F, Blaszczyk E, Schwenke S, Traber J, Funk S, et al. Quantification in cardiovascular magnetic resonance: agreement of software from three different vendors on assessment of left ventricular function, 2D flow and parametric mapping. J Cardiovasc Magn Reson. 2019 Dec;21(1):12.

Clinical Results Differences

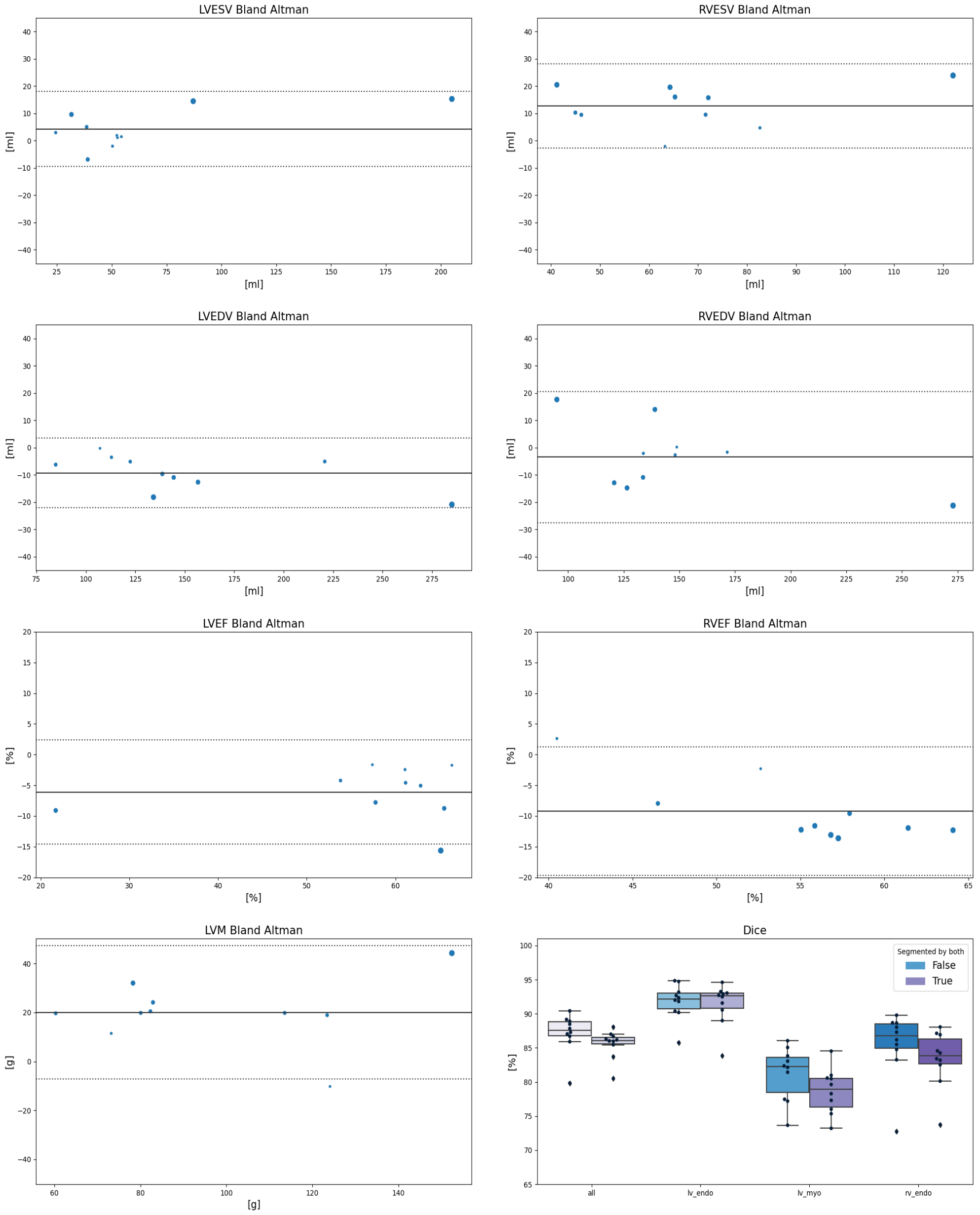

Fig. 1 Clinical Parameter Bland-Altmans: Bland-Altman plots show clinical parameter averages and differences as points for all cases. Point size represents difference, the solid line marks the mean difference between readers, the dashed lines mark the mean differences ±1.96 standard deviations. The last plot offers two Dice boxplots per contour type, one for all images, another restricted to images segmented by both readers. Legend: GUI: Graphical user interface, RV: Right ventricle, LV: Left ventricle, ESV: End-systolic volume, EDV: End-diastolic volume, EF: Ejection fraction, LVM: Left ventricular mass, Dice: Dice similarity coefficient

Confidence Intervals

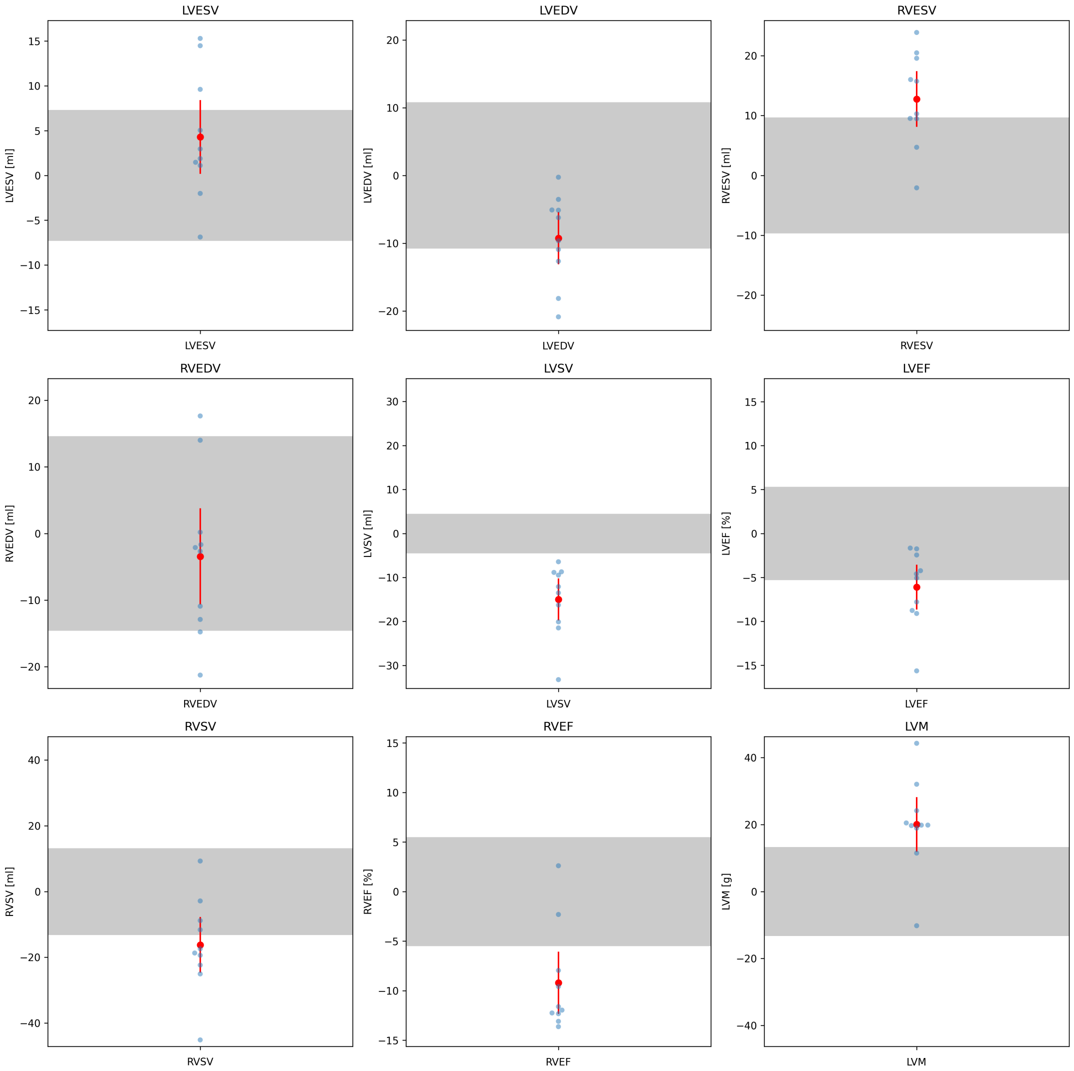

Fig. 2 Tolerance Ranges and Confidence Intervals: Each subfigure references a clinical parameter. Tolerance intervals are shown as gray bars and represent ±1.96 standard deviation of an expert intrareader deviation as derived in another publication (see below). The 95% confidence intervals of the mean value is represented as an error bar in red. Individual clinical parameter differences per case are plotted in blue. Legend: LV: Left ventricle, RV: Right ventricle, ESV: end-systolic volume, EDV: end-diastolic volume, EF: ejection fraction, LVM: Left ventricular myocardium.

Tolerance range paper: Zange L, Muehlberg F, Blaszczyk E, Schwenke S, Traber J, Funk S, et al. Quantification in cardiovascular magnetic resonance: agreement of software from three different vendors on assessment of left ventricular function, 2D flow and parametric mapping. J Cardiovasc Magn Reson. 2019 Dec;21(1):12.

Qualitative Figures added during Manual Inspection

The following PDF pages reference figures, which were manually selected by the investigor and added to this report manually. Every figure has a title and comments that the investigator typed for elaboration.

Title: Slightly underestimated LVESV_bland_altman

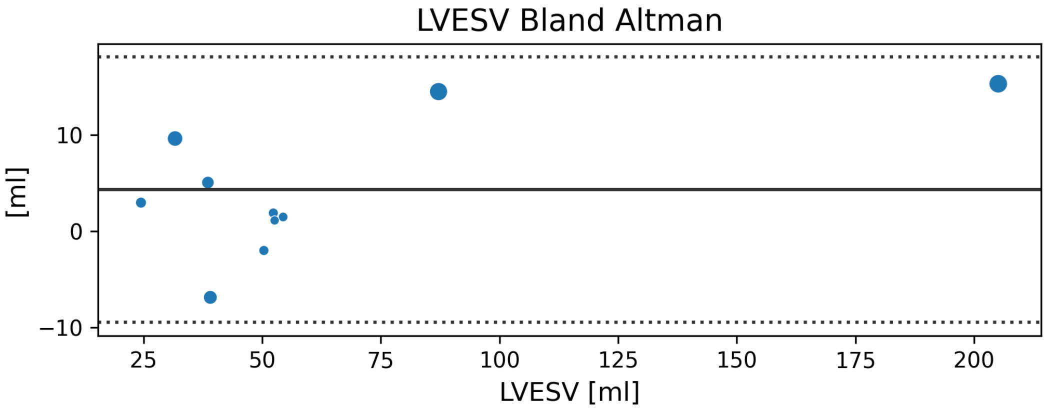

Outside tolerance range, invstigating outliers.

Title: Outlier - overlooked basal Trainee-Case-1_ category: SAX LVES, slice: 3 annotation comparison

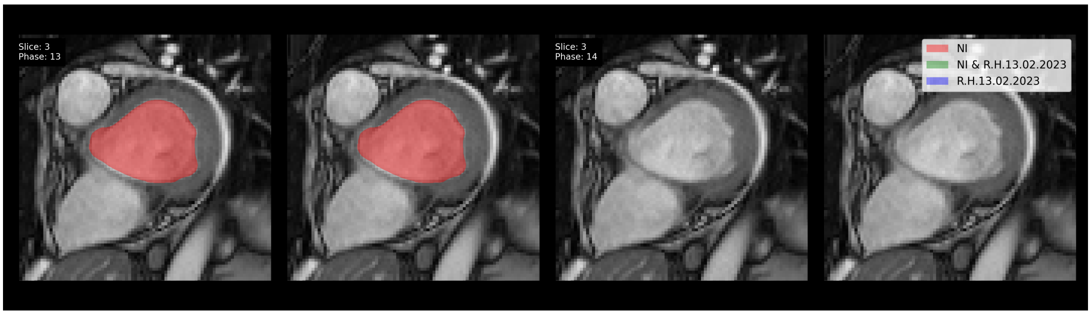

References the above statistical plot.

Title: Overestimation of LVEDV, outside tolerance range LVEDV_bland_altman

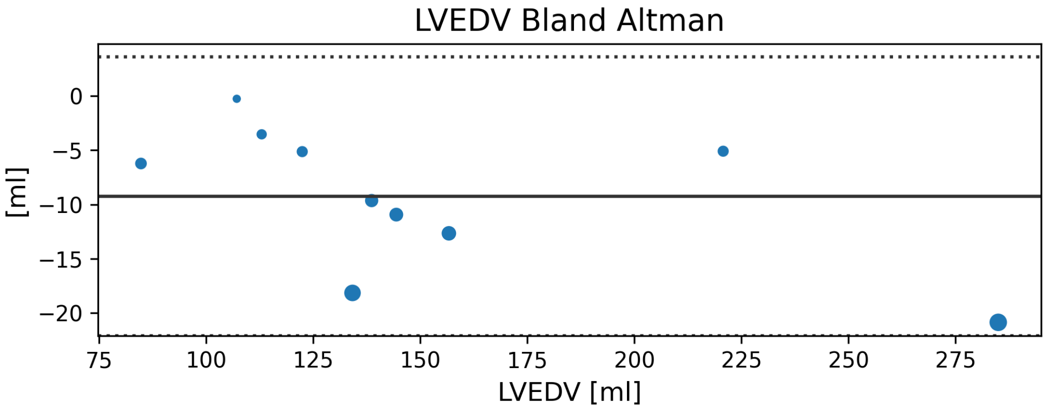

Investigating outliers.

Title: No missegmented basal! Trainee-Case-1_ category: SAX LVED, slice: 5 annotation comparison

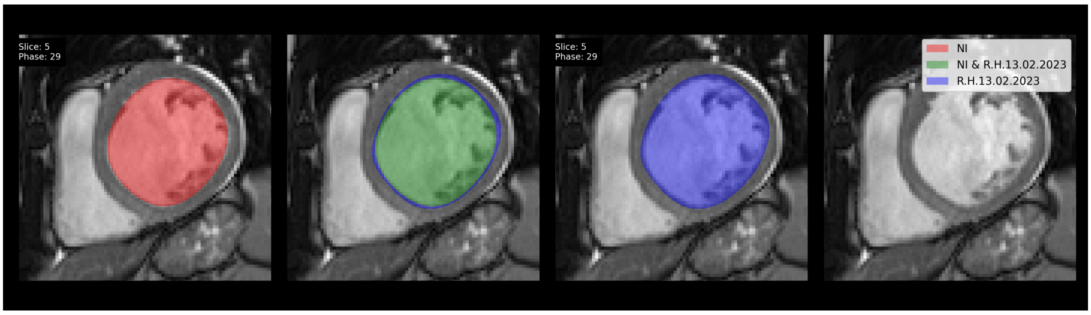

Instead, a small systematic oversegmentation adds up to a large volume difference. approx. -2.5ml per slice.

Title: LVSV outside tolerance range LVSV_bland_altman

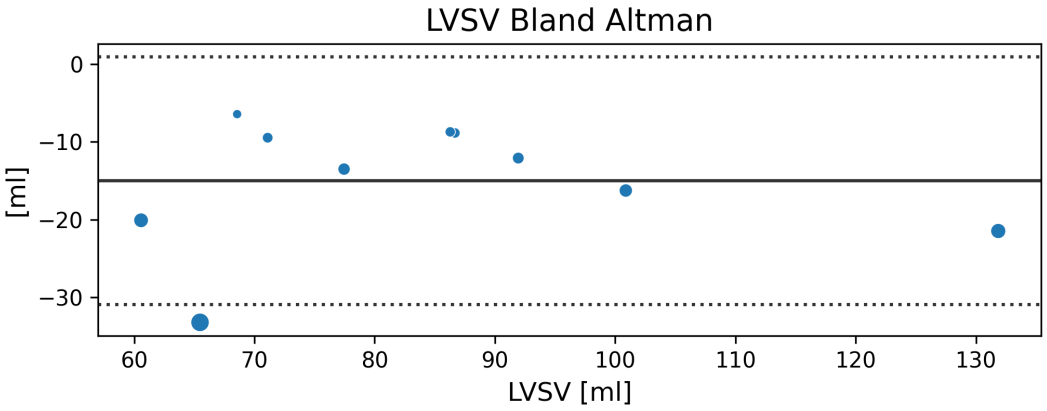

Explained by overlooked basals in LVES and oversegmented slices in LVED.

Title: Systematic underestimation by Trainee RVESV_paired_boxplot

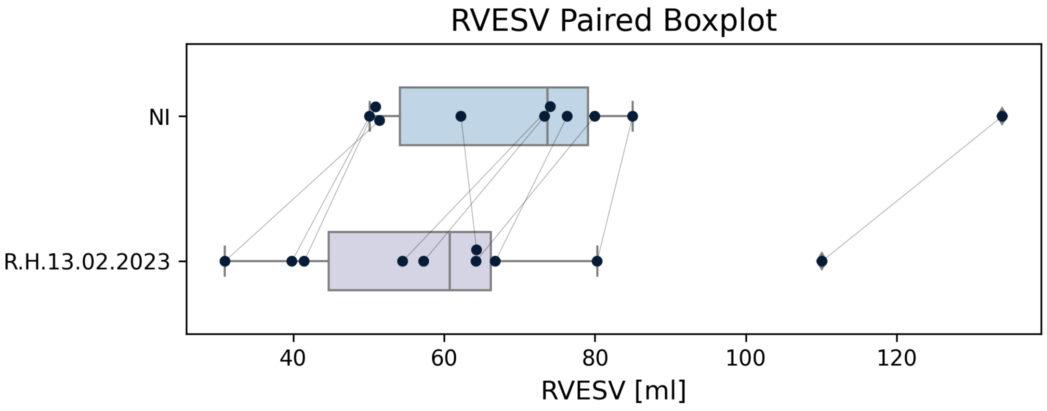

Investigating outliers.

Title: Small differences adding up Trainee-Case-5_ category: SAX RVES, slice: 9 annotation comparison

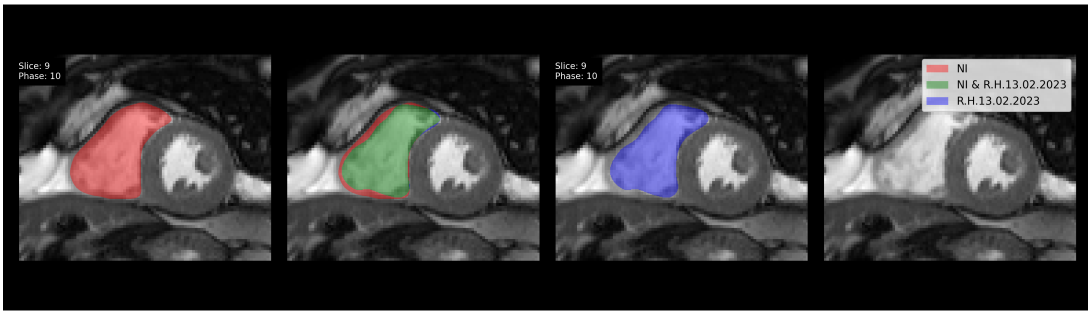

Trainee excluded too much RV papillary muscle from RV volume.

Title: RVEDV inside tolerance range RVEDV_bland_altman

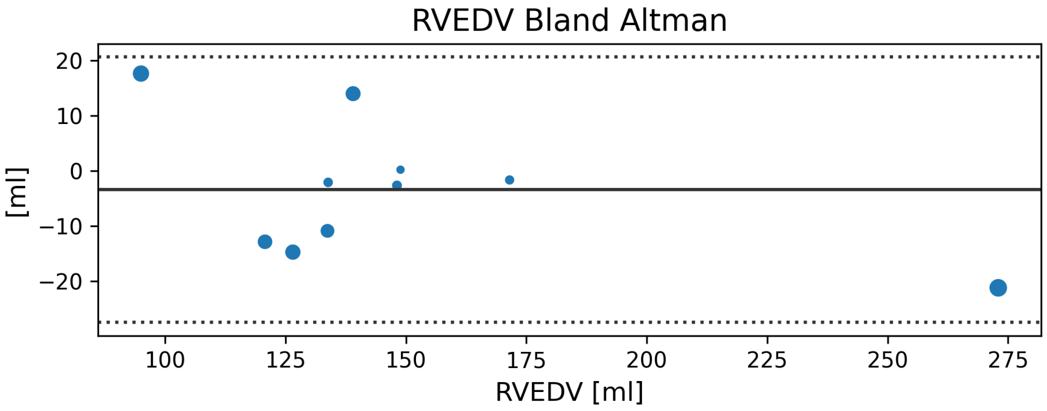

No further investigation.

Title: RVSV outside tol range RVSV_bland_altman

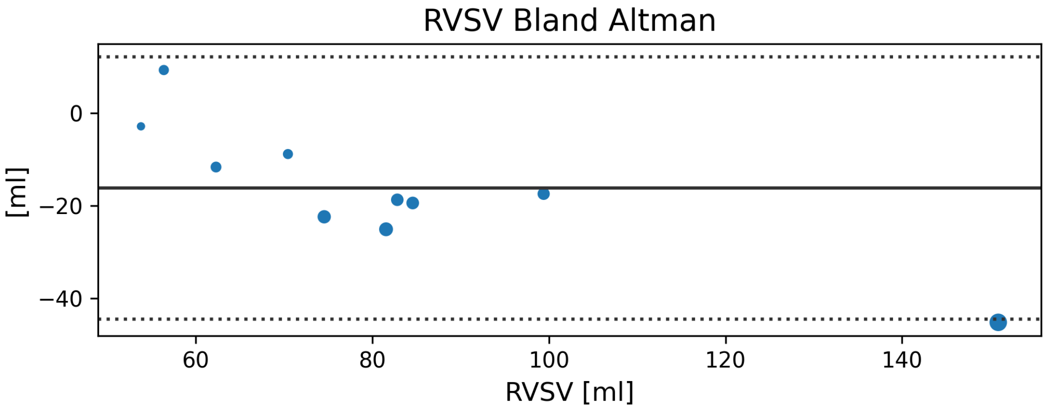

Investigating outlier.

Title: Oversegmentation of RV Basal Trainee-Case-5_ category: SAX RVED, slice: 3 annotation comparison

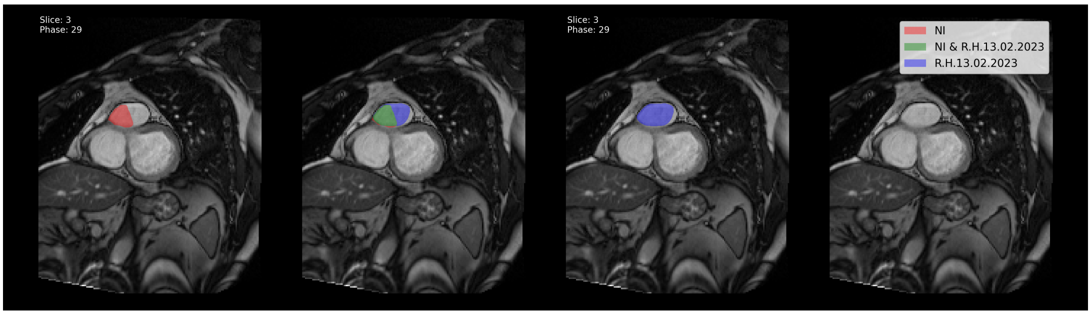

Following image belongs to same case, with higher impact.

Title: Misinterpretation of basal RV Trainee-Case-5_ category: SAX RVED, slice: 4 annotation comparison

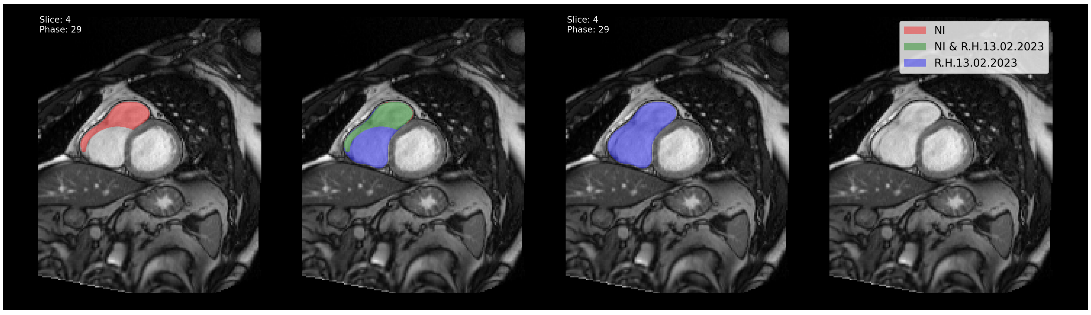

Large volume difference: -18.2 ml

Title: LVM outside tolerance range LVM_paired_boxplot

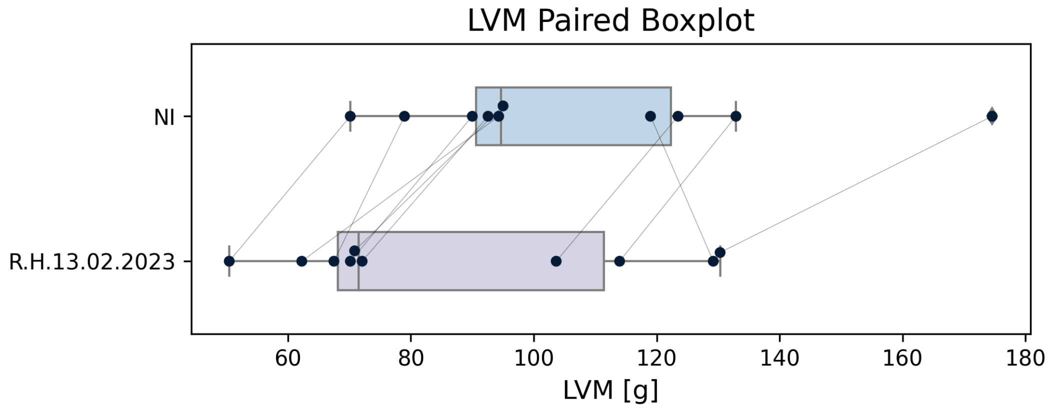

Systematic undersegmentation of LVM, investigating outliers.

Title: Thin myocardium Trainee-Case-2_ category: SAX LVED, slice: 7 annotation comparison

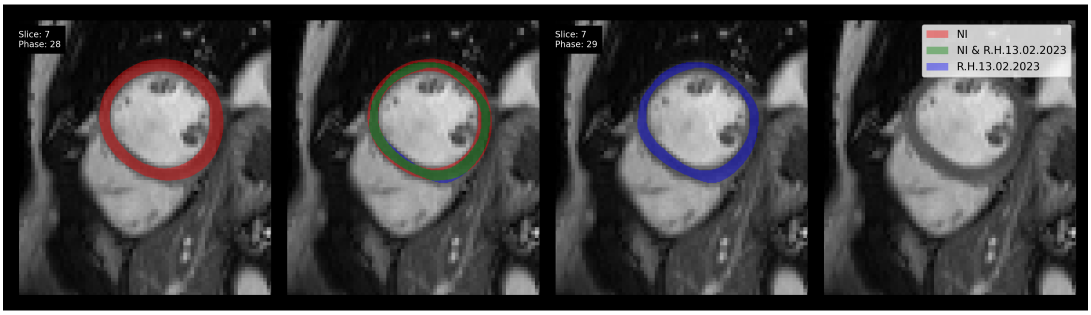

These thinner segmentations add up, approx. 3ml per slice.

Clinical Parameter Means and Tolerance Ranges

Clinical Result (mean±std)	NI	R.H.13.02.2023	Diff(NI, R.H.13.02.2023)	(Mean Diff±CI), ±Tol range
GLOBAL_T1 [ms]	1018.8 (50.0)	1023.3 (52.0)	-0.5 (8.8)	(-5.9, 5.0), ±24.5

Table. 1 This table shows the clinical parameter names in the first column. The other columns show statistics concerning the parameters. The first and second readers' means (stds) are shown in the second and third column, respectively. The mean and std of the differences between both readers is presented in the fourth column. The mean difference of both readers ± 95% confidence intervals are shown in parentheses with ±tolerance ranges thereafter. This provides information on whether the 95% estimate of the mean difference between both readers is within an acceptable limit.

Overview Assessment

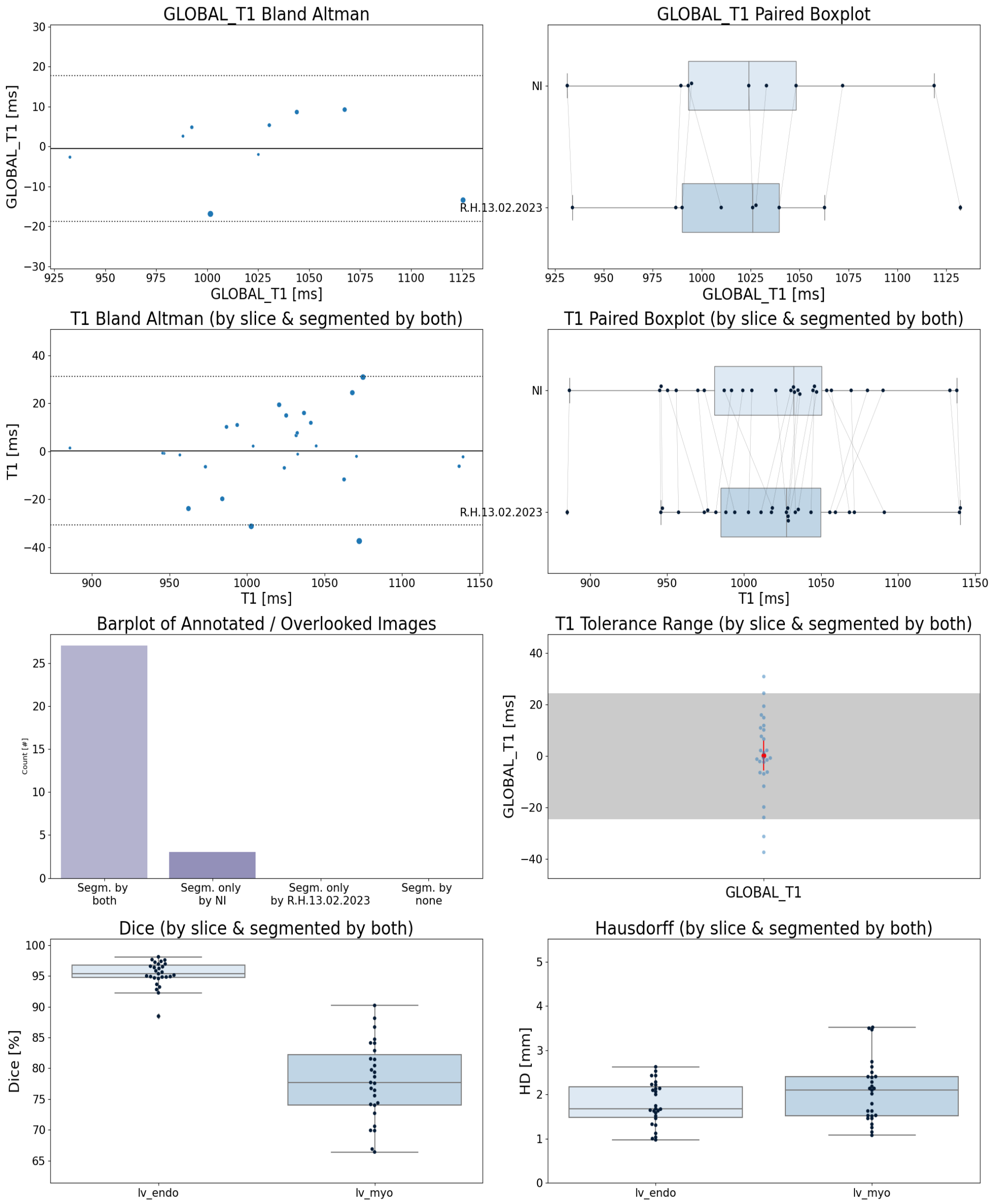

Fig. 1 Overview of Mapping values and Contour Metrics: Bland-Altman plots for Mapping values as points for all cases / all slices (first /second row). Point size shows magnitude of difference, the solid line marks mean difference between readers, the dashed lines mark mean differences ±1.96 standard deviations. Paired Boxplots show Mapping values as assessed by the first reader (on top) and the second reader below for all cases / all slices (first / second row). Lines connect same cases / slices to one another. Row four contains a histogram and a tolerance range plot. The histogram shows nr of slices contoured by both readers / only first reader / only second reader or not by either. The tolerance range is shown for all slices segmented by both readers (excluding "overlooked" slices). The gray bars represent ±tolerance range. The ±95% confidence interval is plotted as an errorbar in red around the average difference. The case value differences are plotted in blue. In the fourth row (left) dice values are plotted per contour type. On the right Hausdorff distance values are plotted per contour type. Legend: Dice: Dice similarity coefficient, HD: Hausdorff distance

Reference Point Differences

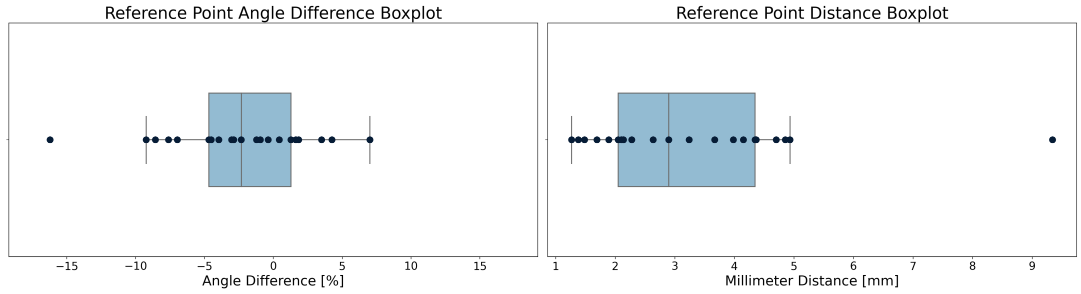

Fig. 2 Reference Point Difference Plots: On the left the angle differences between readers per slice are plotted as a scatter plot on top of a boxplot. The angles are defined as the angle between the line between the endocardial median point and the reference point. On the right the distances between reference points selected by both readers are plotted as a scatter plot on top of a boxplot. Legend: mm: Millimeter

Avg Differences AHA Model

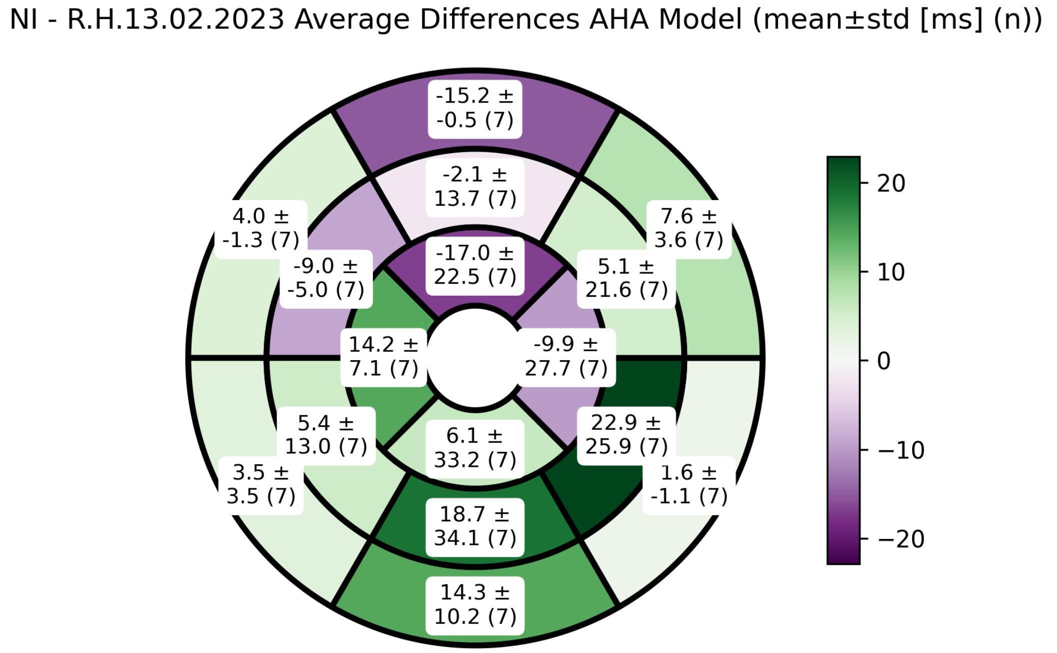

Fig. 5 Average Differences AHA Model: The AHA model is plotted for 16 segments reflecting the basal (6 outer segments), midventricular (6 middle segments) and apical (4 inner segments). Each segment contains a label with the mean±standard deviation (n). The mean and standard deviation pertain to the pixel value differences per segmet between the two readers. In parentheses the number of cases that provided values to this segment by both readers is shown. Legend: AHA: American Heart Association

Qualitative Figures added during Manual Inspection

The following PDF pages reference figures, which were manually selected by the investigor and added to this report manually. Every figure has a title and comments that the investigator typed for elaboration.

Title: Inside tolerance range, good agreement GLOBAL_T1_paired_boxplot

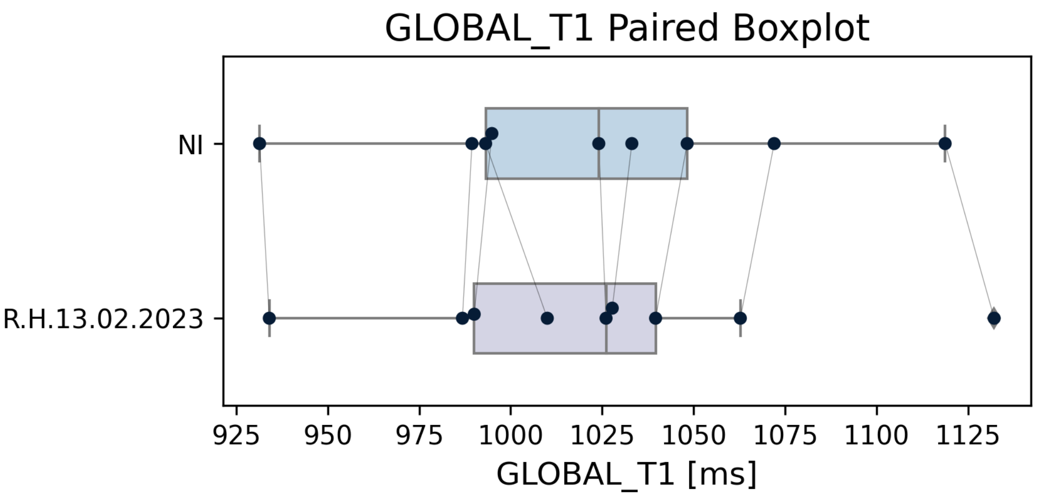

Title: Good contours Case: Trainee-Case-4_, slice: 1

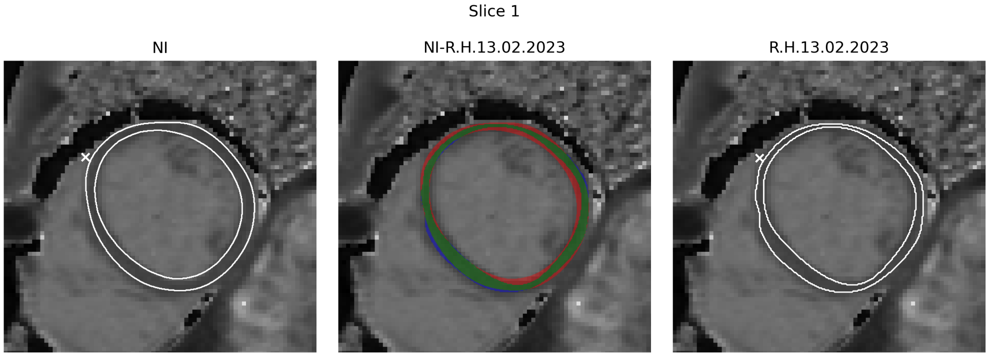

R.H. slighly more conservative contours

Title: Apex as well Case: Trainee-Case-4_, slice: 2

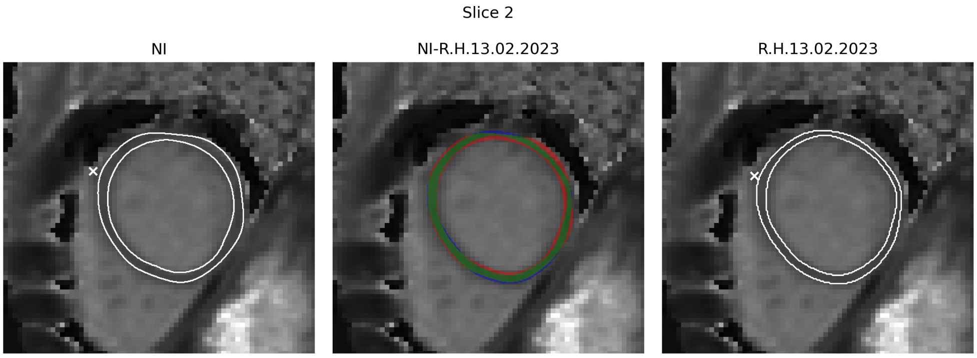

Title: Possible disagreement with NI? Case: Trainee-Case-1_, slice: 1

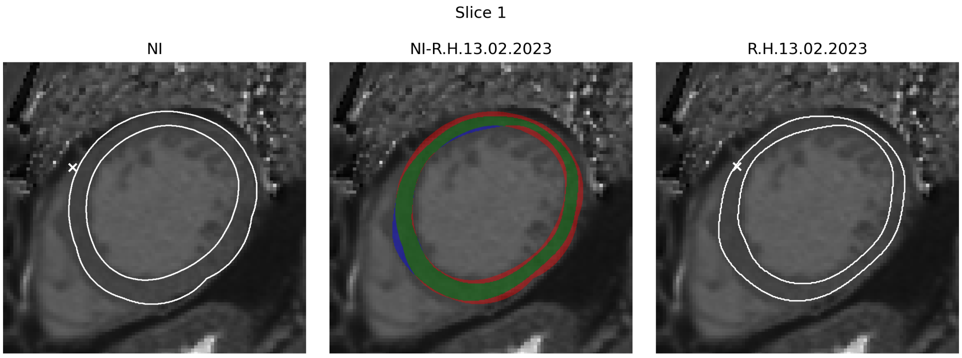

RH has more conservative contours, they seem appropriate: for NI there are low T1 values outside myocardium in inferior, inferolateral and anterior segments?

Title: These segments also have... Case: Trainee-Case-1_, slice: 1

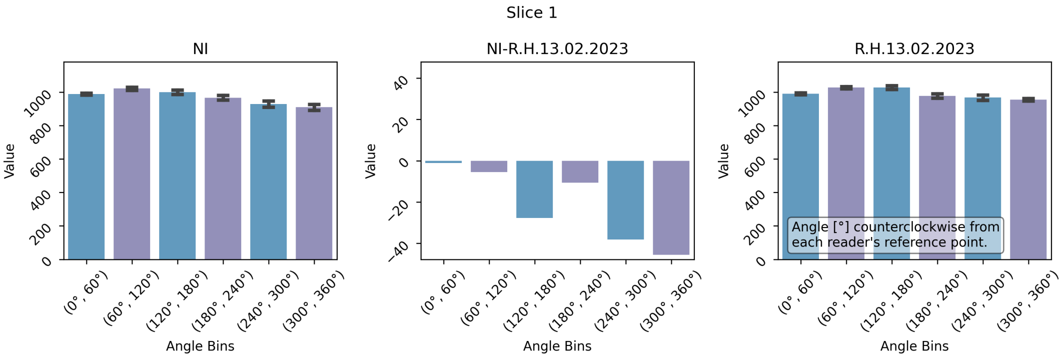

siginificantly lower values for the NI.

Clinical Parameter Means and Tolerance Ranges

Clinical Result (mean±std)	NI	R.H.13.02.2023	Diff(NI, R.H.13.02.2023)	(Mean Diff±CI), ±Tol range
GLOBAL_T2 [ms]	49.6 (1.6)	49.2 (1.9)	0.4 (0.6)	(-0.0, 0.7), ±3.2

Table. 1 This table shows the clinical parameter names in the first column. The other columns show statistics concerning the parameters. The first and second readers' means (stds) are shown in the second and third column, respectively. The mean and std of the differences between both readers is presented in the fourth column. The mean difference of both readers ± 95% confidence intervals are shown in parentheses with ±tolerance ranges thereafter. This provides information on whether the 95% estimate of the mean difference between both readers is within an acceptable limit.

Overview Assessment

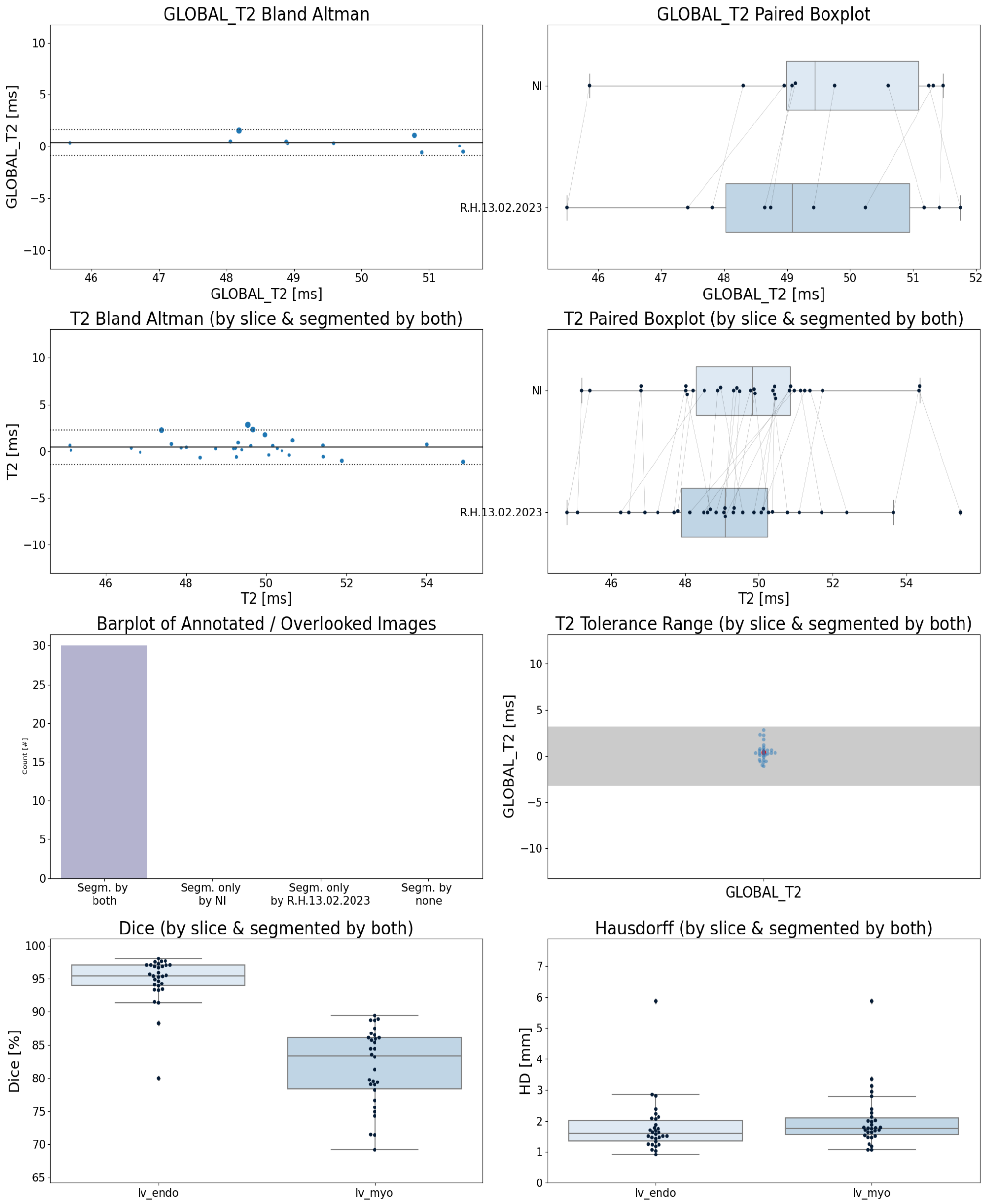

Fig. 1 Overview of Mapping values and Contour Metrics: Bland-Altman plots for Mapping values as points for all cases / all slices (first /second row). Point size shows magnitude of difference, the solid line marks mean difference between readers, the dashed lines mark mean differences ±1.96 standard deviations. Paired Boxplots show Mapping values as assessed by the first reader (on top) and the second reader below for all cases / all slices (first / second row). Lines connect same cases / slices to one another. Row four contains a histogram and a tolerance range plot. The histogram shows nr of slices contoured by both readers / only first reader / only second reader or not by either. The tolerance range is shown for all slices segmented by both readers (excluding "overlooked" slices). The gray bars represent ±tolerance range. The ±95% confidence interval is plotted as an errorbar in red around the average difference. The case value differences are plotted in blue. In the fourth row (left) dice values are plotted per contour type. On the right Hausdorff distance values are plotted per contour type. Legend: Dice: Dice similarity coefficient, HD: Hausdorff distance

Reference Point Differences

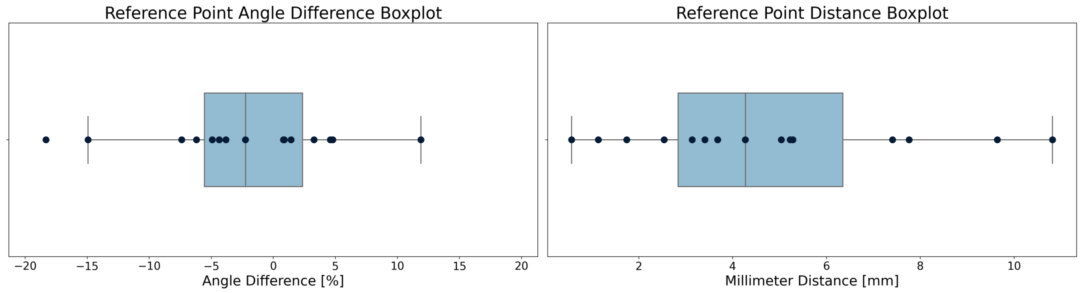

Fig. 2 Reference Point Difference Plots: On the left the angle between readers per slice are plotted as a scatter plot on top of a boxplot. The angles are defined between the line spanned by the endocardial median and the reference point. On the right the reference point distances are plotted as a scatter plot on top of a boxplot. Legend: mm: Millimeter

Avg Differences AHA Model

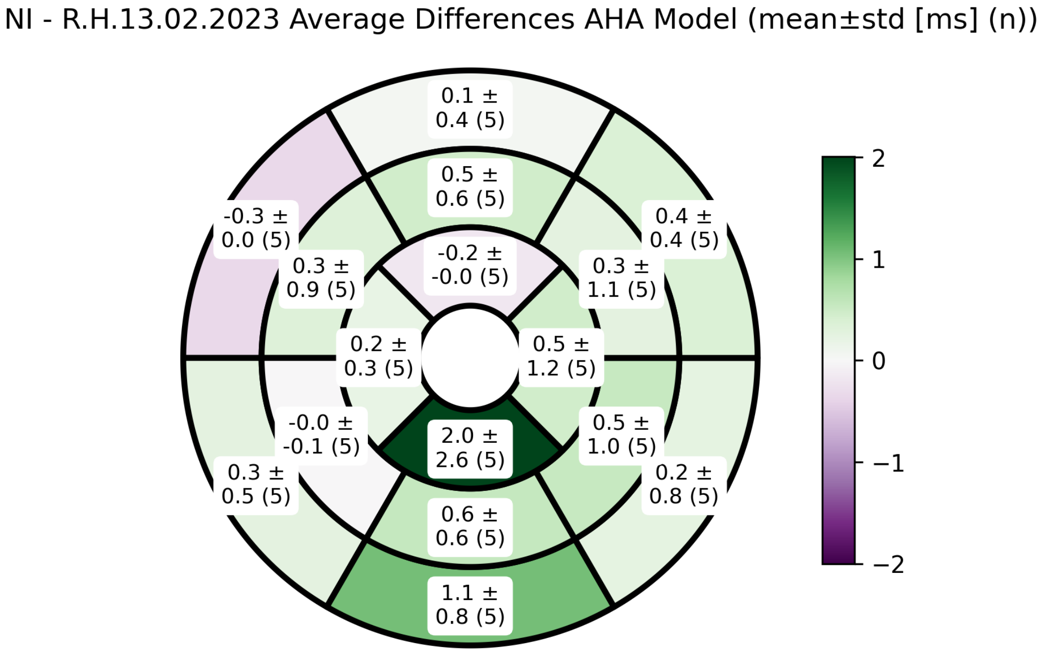

Fig. 5 Average Differences AHA Model: The AHA model is plotted for 16 segments reflecting the basal (6 outer segments), midventricular (6 middle segments) and apical (4 inner segments). Each segment contains a label with the mean±standard deviation (n). The mean and standard deviation pertain to the pixel value differences per segmet between the two readers. In parentheses the number of cases that provided values to this segment by both readers is shown. Legend: AHA: American Heart Association

Qualitative Figures added during Manual Inspection

The following PDF pages reference figures, which were manually selected by the investigor and added to this report manually. Every figure has a title and comments that the investigator typed for elaboration.

Title: Good agreement, inside tolerance range GLOBAL_T2_bland_altman

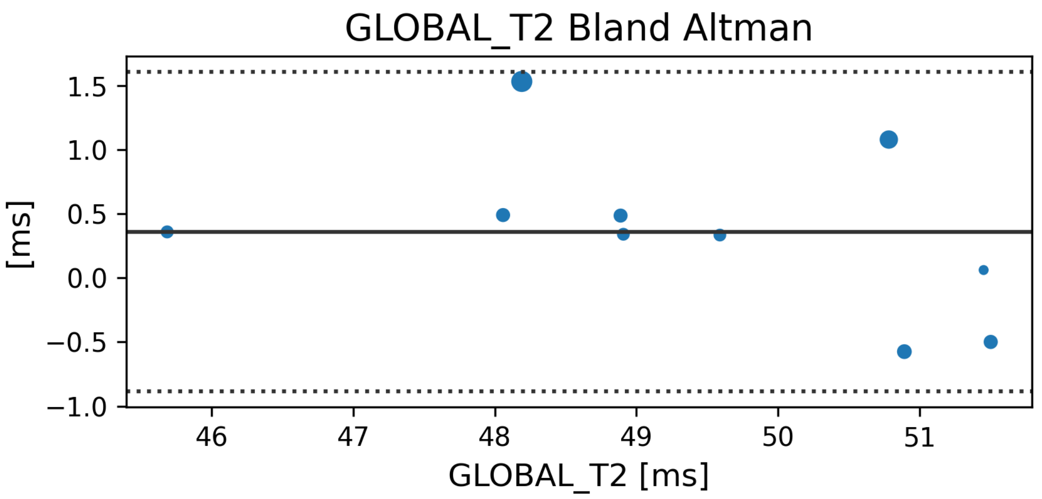

Investigating upper outlier.

Title: Overlooked reference point Case: Trainee-Case-9_, slice: 1

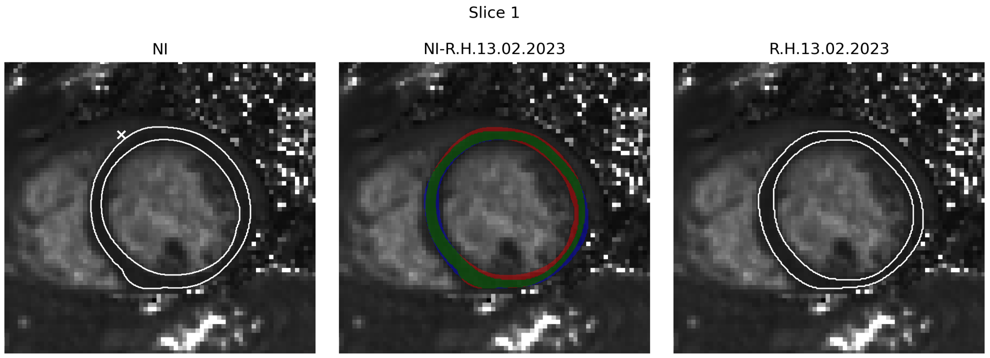

Title: Another overlooked reference point Case: Trainee-Case-8_, slice: 0

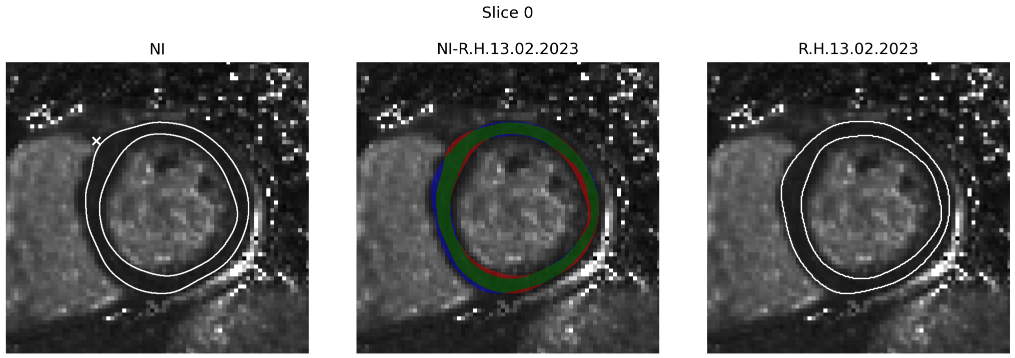

Otherwise good values

Title: Here's a reference point! Trainee-Case-1_ category: SAX T2, slice: 2 annotation comparison

Category: SAX T2, slice: 2

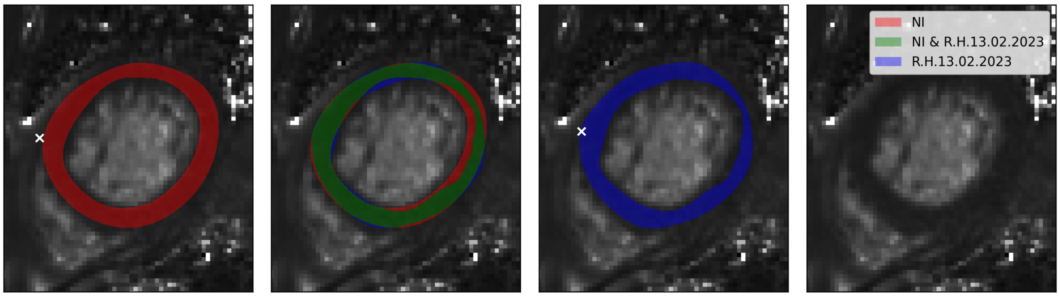

Not systematic, just overlooked.

Clinical Parameter Means and Tolerance Ranges

Clinical Result (mean±std)	NI	R.H.13.02.2023	Diff(NI, R.H.13.02.2023)	(Mean Diff±CI), ±Tol range
4CV_RAEDAREA [cm^2]	23.5 (5.5)	22.5 (4.3)	1.1 (2.2)	(-0.3, 2.4), ±1.0
4CV_RAEDV [ml]	82.2 (29.3)	76.2 (20.4)	5.9 (14.5)	(-3.1, 14.9), ±nan
4CV_LAEDAREA [cm^2]	20.4 (4.3)	20.7 (4.9)	-0.3 (2.4)	(-1.8, 1.1), ±2.1
4CV_LAEDV [ml]	59.9 (21.2)	63.0 (22.4)	-3.1 (7.4)	(-7.7, 1.5), ±nan
2CV_LAEDAREA [cm^2]	19.8 (3.7)	18.7 (6.9)	1.1 (3.7)	(-1.2, 3.4), ±2.0
2CV_LAEDV [ml]	60.6 (15.3)	66.2 (12.9)	-1.9 (3.6)	(-4.1, 0.4), ±nan
BIPLANE_LAEDV [ml]	63.8 (16.8)	69.0 (16.5)	-2.1 (3.3)	(-4.1, -0.1), ±nan
LAEDP_4CV [#]	13.5 (2.1)	12.7 (2.5)	1.4 (1.0)	(0.8, 2.0), ±nan
LAEDP_2CV [#]	13.5 (2.1)	12.7 (2.7)	1.4 (1.1)	(0.8, 2.1), ±nan
RAEDP_4CV [#]	13.4 (1.8)	12.7 (2.5)	1.3 (1.1)	(0.6, 2.0), ±nan

Table. 1 This table shows the clinical parameter names in the first column. The other columns show statistics concerning the parameters. The first and second readers' means (stds) are shown in the second and third column, respectively. The mean and std of the differences between both readers is presented in the fourth column. The mean difference of both readers ± 95% confidence intervals are shown in parentheses with ±tolerance ranges thereafter. This provides information on whether the 95% estimate of the mean difference between both readers is within an acceptable limit.

Atrial Area Differences

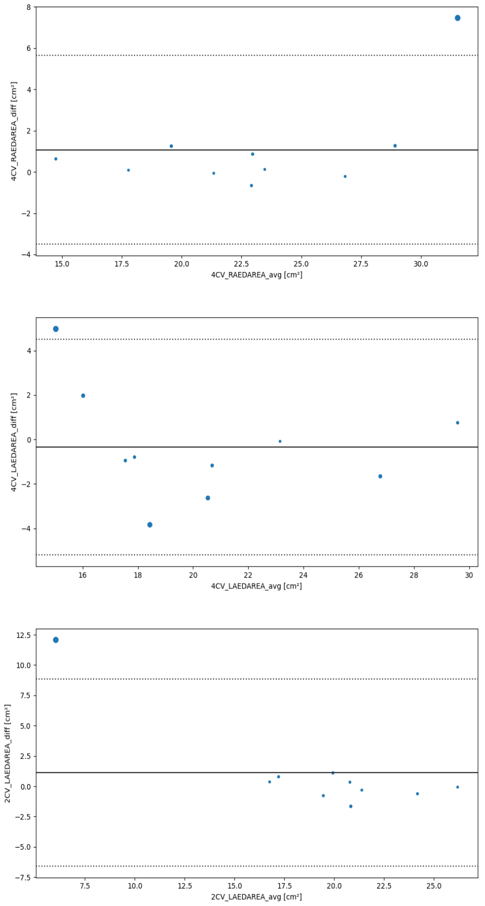

Fig. 1 Area Differences for LA & RA: The first row presents Bland-Altman plots for the 4CV RA areas in ES and ED. The second row shows BA plots for the 4CV LA areas. The third row shows 2CV LA areas. The last row contains Dice value boxplots per contour on the left and Hausdorff distance boxplots on the right. Legend: ES: End-systole, ED: End-diastole, CV: Chamber View, LA: Left Atrium, RA: Right Atrium, Dice: Dice similarity coefficient

Confidence Intervals and Tolerance Ranges

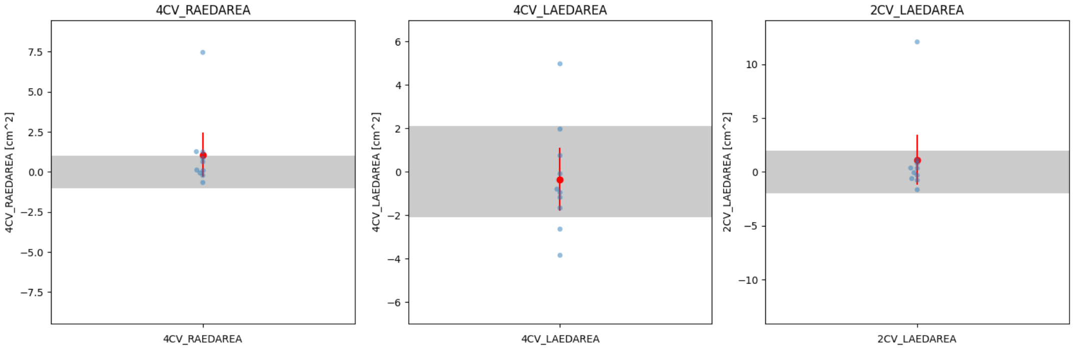

Fig. 2 Confidence Intervals and Tolerance Ranges of Atria Areas: Each subfigure references an atrial area, from left to right, 4CV RA, 4CV LA, 2CV LA. Tolerance intervals are shown as gray bars and represent ±1.96 standard deviation of an expert intrareader deviation. The 95% confidence intervals of the mean area difference is represented as an error bar in red. Individual area differences per contour are plotted in blue. Legend: CV: Chamber view, RA: Right Atrium, LA: Left Atrium

Qualitative Figures added during Manual Inspection

The following PDF pages reference figures, which were manually selected by the investigor and added to this report manually. Every figure has a title and comments that the investigator typed for elaboration.

Title: Good estimation of RAED 4CV_RAEDAREA_paired_boxplot

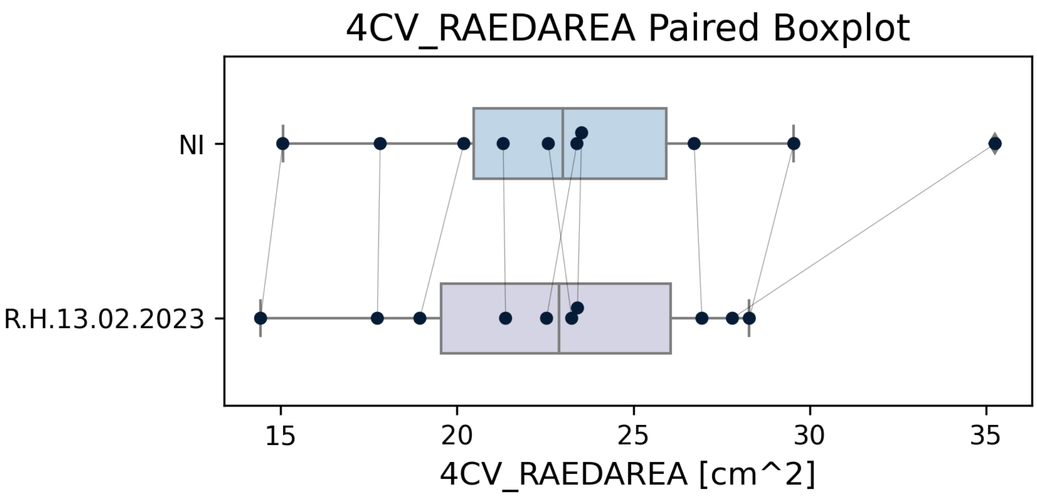

However, one significant outlier. Investigating...

Title: Trainee mistook cardiac structure Trainee-Case-5_ category: LAX 4CV RAED, slice: 0 annotation comparison

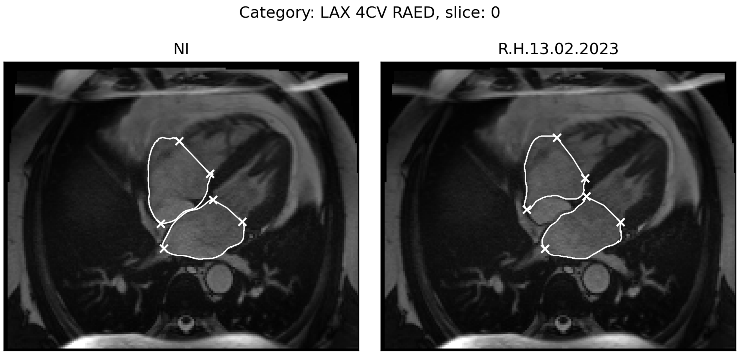

Possible considered Aorta in 4CV? Lead to large area difference.

Title: Here, in color. Trainee-Case-5_ category: LAX 4CV RAED, slice: 0 annotation comparison

Category: LAX 4CV RAED, slice: 0

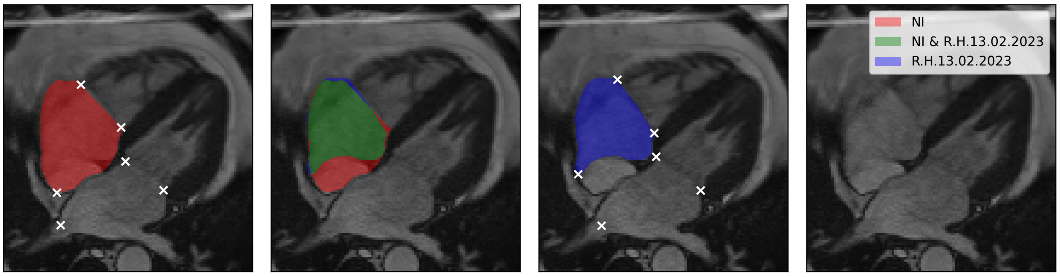

Title: Good average, inside tolerance range 4CV_LAEDAREA_paired_boxplot

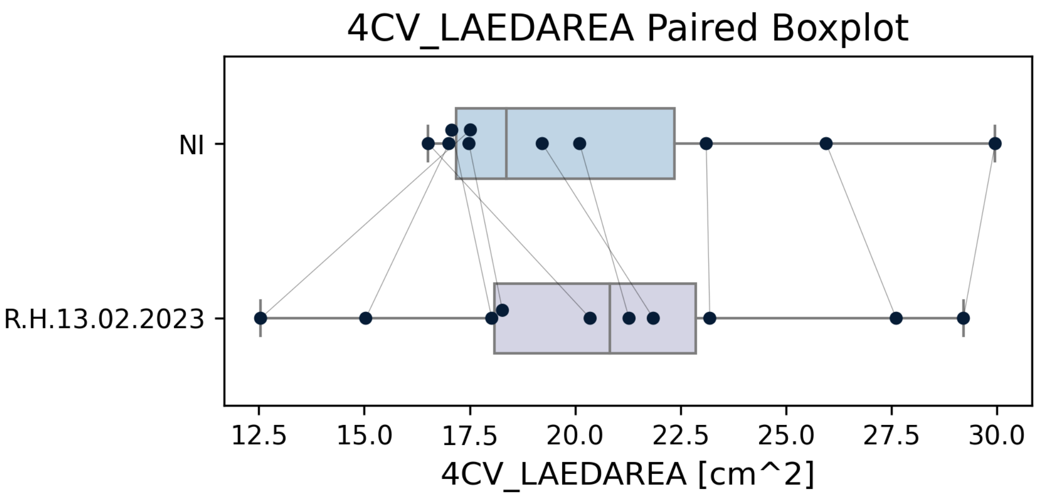

However, strong deviations in either direction. Investigating outliers.

Title: Trainee has more conservative ... Trainee-Case-1_ category: LAX 4CV LAED, slice: 0 annotation comparison

Category: LAX 4CV LAED, slice: 0

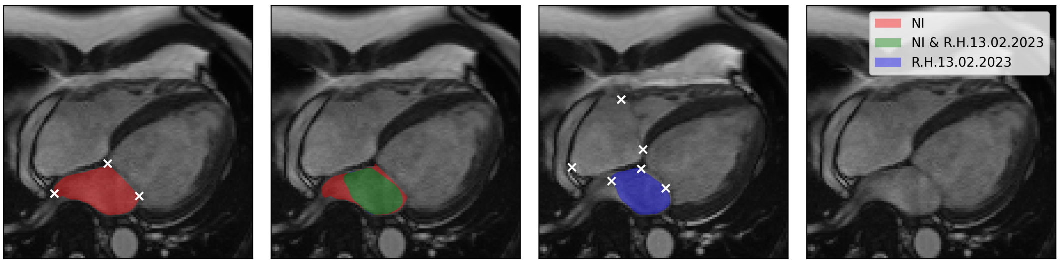

contours for the left atrium (-1.3cm^2)

Title: Second outlier is ... Trainee-Case-4_ category: LAX 4CV LAED, slice: 0 annotation comparison

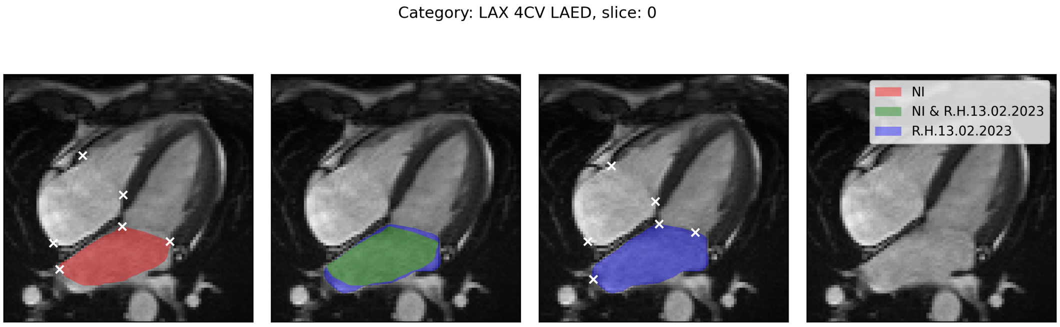

just a large atrium, with difficult/blurry edges.

Title: 2 CV is excellent, except for one... 2CV_LAEDAREA_paired_boxplot

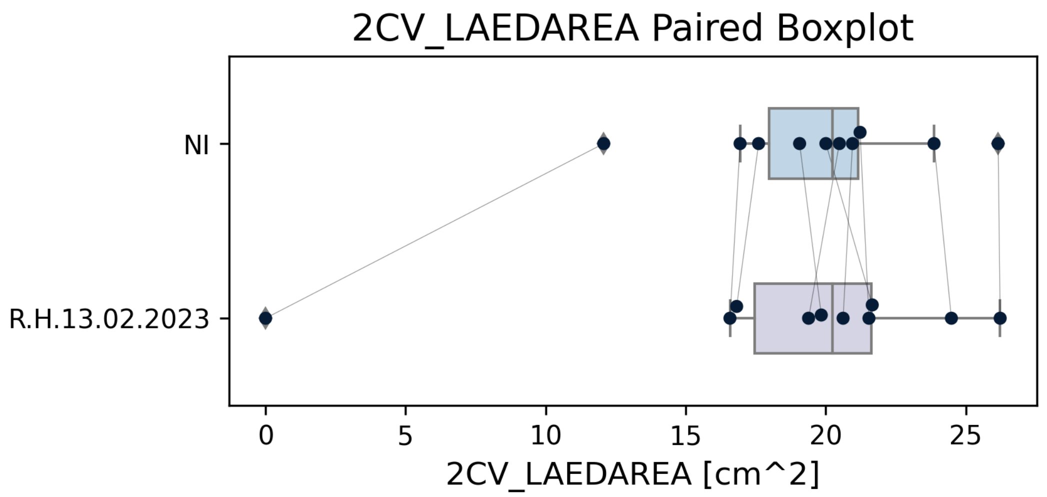

overlooked?

Title: Overlooked by trainee. Trainee-Case-4_ category: LAX 2CV LAED, slice: 0 annotation comparison

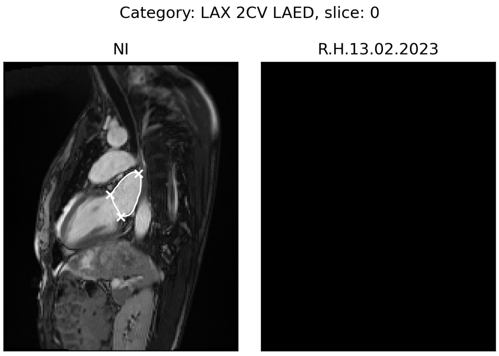

Supplement: Supplementary file 7 — Supplementary Material 7 [file 12911_2025_3271_MOESM7_ESM.pdf]
